# Integrated nutrition-sensitive interventions are associated with improvements in household food security and dietary diversity among vulnerable women in the host communities of Cox’s Bazar, Bangladesh: Findings from a pre-post comparison study

**DOI:** 10.1371/journal.pone.0362124

**Published:** 2026-11-05

**Authors:** Munia Afroz, Sakib Rahman, Fahmida Akter, Md. Mokbul Hossain, Mahbub A.H.M. Latif, Owen White Nkhoma, Jecinter Akinyi Oketch, Raihana Bhuiyan, Kiross Tefera Abebe, Deepika Mehrish Sharma, Farhana Sharmin, Mohammad Zahidul Manir, Md. Lalan Miah, Anjuman Tahmina Ferdous, Rina Rani Paul, Malay Kanti Mridha

**Affiliations:** 1 Centre for Non-communicable Diseases and Nutrition, BRAC James P Grant School of Public Health, BRAC University, Dhaka, Bangladesh; 2 Institute of Applied Statistics and Data Science (IASDS), University of Dhaka, Dhaka, Bangladesh; 3 UNICEF, Cox’s Bazar Field Office, Bangladesh; 4 UNICEF, Bangladesh; Jashore University of Science and Technology (JUST), BANGLADESH

## Abstract

**Introduction:**

Nutrition-sensitive interventions aim to improve or at least safeguard the underlying causes of malnutrition, especially among the most vulnerable populations. Integrated nutrition-sensitive interventions are effective strategies for improving household food security and dietary diversity in low-income settings. In 2021, the European Union (EU) and UNICEF, in collaboration with the Government of Bangladesh and partners, implemented nutrition-sensitive interventions combining two income-generating activities (IGAs): backyard poultry rearing and tailoring with asset transfers, direct cash support, and nutrition education for vulnerable women in the host communities of Cox’s Bazar, Bangladesh. We evaluated the association of these interventions with household food security and dietary diversity among vulnerable women.

**Materials and methods:**

We analyzed data from two rounds (2022 and 2024) of repeated cross-sectional surveys involving 1,255 vulnerable women (638 in 2022 and 617 in 2024) across four upazilas in Cox’s Bazar, Bangladesh. Covariates were initially imbalanced across groups because the interventions were not randomly assigned; therefore, we employed propensity score weighting to balance the covariates between the two groups. We used modified Poisson regression and binomial regression models to estimate the association between exposure and outcomes from the pseudo-population obtained by propensity score weighting. All the inferences in this manuscript were based on a 5% significance level.

**Results:**

In 2024, vulnerable women had a 53% higher likelihood of household food security (RR = 1.53, 95% CI: 1.27–1.85, p < 0.001) than in 2022. Similarly, dietary diversity of vulnerable women increased markedly, showing over two and a half-fold higher likelihood of adequate dietary diversity in 2024 (RR = 2.78, 95% CI: 2.36–3.27, p < 0.001). The E-values corresponding to the associations of food security and dietary diversity with survey year were 2.44 and 5.00, respectively, on the risk ratio scale, which shows that the findings were less sensitive to unmeasured confounders.

**Conclusion:**

Household food security and dietary diversity substantially increased over time among vulnerable women in the study areas. As the study was a two-repeated cross-sectional survey, residual confounding and other time-varying contextual factors cannot be excluded, so the observed differences should not be interpreted as definitive causal effects of the intervention. Future controlled or longitudinal evaluations are needed to establish the causal effectiveness and long-term sustainability of integrated nutrition-sensitive interventions.

## Introduction

The nutrition-sensitive term is defined as the interventions or development efforts that aim to improve or at least avoid harm to the underlying and immediate determinants of nutrition, such as adequate food access, health services, care practices, and a healthy environment, particularly for the most nutritionally vulnerable individuals and populations [1]. Integrated nutrition-specific and nutrition-sensitive interventions are essential to achieve a long-term and sustainable solution for undernutrition [2,3]. While nutrition-specific interventions directly influence nutritional outcomes, nutrition-sensitive interventions modify the underlying factors of malnutrition [1]. Nutrition-sensitive interventions can target local food production, address food insecurity, and protect vulnerable populations from adverse situations [4,5]. Nutrition-sensitive interventions, such as backyard poultry rearing, are recognized as an effective approach for improving the nutritional status of people living in low-income settings by generating income, empowering women, and ensuring access to high-quality foods, such as eggs and meat [6,7]. Tailoring is another nutrition-sensitive approach used in community-based settings to improve the nutrition of women [8].

Cox's Bazar, a vulnerable coastal region in Bangladesh, is home to approximately 2.82 million people [9]. It is recognized as one of the most disaster-prone areas and is frequently exposed to strong winds, cyclones, and coastal flooding [10,11]. These natural calamities have a severe impact on the livelihoods of the people in host communities [11]. Furthermore, the large influx of Rohingya refugees in 2017 increased the demand for resources and exerted huge pressure on their livelihoods. In our context, the host community refers to the local Bangladeshi citizens who lived, especially in the areas of Teknaf and Ukhiya, before and after the Rohingya influx [12]. Host communities in Cox’s Bazar primarily depend on natural resources for their livelihoods; however, continuous deforestation and the degradation of land and vegetation have created challenging situations for these communities [13]. A study conducted by Karim and Zhang, aimed to explore factors leading to vegetative loss in Ukhiya and Teknaf and showed that in 2017–2019, massive vegetation land loss (79.6%) occurred in host communities across 34 refugee camps in Cox’s Bazar [14]. Vegetative land loss disrupted crop production in the nearby areas of the camps, and these changes had a lasting effect on the food security and dietary diversity of host communities [12].

Food security refers to a condition characterized by constant physical, social, and economic access to sufficient quantities of safe and nutritious foods that meet an individual’s dietary needs and preferences, are necessary for normal developmental processes and growth, and thereby enable an active and healthy lifestyle [15]. Food security improves through household-level food availability, access, utilization, and stability [1]. A nationally representative survey conducted in 2018 and 2023 indicated a decline in food security in Bangladesh over the following years [16]. Notably, Cox’s Bazar was identified by the International Food Policy Research Institute (IFPRI) as the third most food-insecure district in Bangladesh, with a food insecurity score of 4.6 [17]. Moreover, food security is significantly associated with dietary diversity [18–20]. Household access to a variety of foods can be assessed using dietary diversity, a qualitative measure of food consumption that serves as an indicator of nutrient adequacy [19]. The principle of dietary diversity highlights that diversified foods are essential for maintaining an individual’s optimal health [21]. In most low-income countries, women's diets are monotonous, primarily cereal-based, and often consist of low-energy meals [22,23]. The nationally representative Food Security and Nutrition Surveillance-National Nutrition Services (FSNS-NNS) survey in Bangladesh reported a decline in mean dietary diversity scores among women aged 10–49 years, from 4.4 in 2011 to 4.1 in 2015 [24]. Additionally, a recent study conducted in St. Martin's Island found that only 40.3% of women met the minimum dietary diversity [25]. Studies have shown that nutrition-sensitive interventions increase food availability and accessibility at the household level, redirecting the increase in the consumption of high-nutrient-content foods, and food purchasing power also increases through income generation [26]. Faber et al. revealed that food-insecure household members consumed less diversified foods than food-secure households [27]. The vulnerability of the host community in Cox’s Bazar stems from economic fragility and climatic shocks that jeopardize the food-purchasing power and food security of host communities [28,29]. Accordingly, poverty and limited livelihood opportunities often hinder households from accessing diverse and nutritious diets [30].

In response to the nutritional challenges faced by host communities in Cox’s Bazar, the European Union (EU) and the United Nations Children's Fund (UNICEF), in collaboration with the Government of Bangladesh and other partners, implemented an integrated project in 2021 focusing on nutrition-specific and nutrition-sensitive services. The BRAC James P Grant School of Public Health (BRAC JPGSPH) documented the process and evaluated the outcomes by pre-post comparison of the project. Nutrition-sensitive interventions included two income-generating activities (IGA), backyard poultry rearing and tailoring, aimed at promoting household-level food accessibility, dietary diversification, and improving livelihoods for vulnerable women. In addition, IGA beneficiaries were also linked to existing nutrition-specific services at community clinics and *upazila* health complexes.

These integrated interventions aimed to improve household food security and dietary diversity among vulnerable women in the host communities of Cox’s Bazar. Given the targeted nature of the intervention, it was essential to assess the association of these interventions with food security and dietary diversity. Moreover, there is limited literature on the association of such integrated interventions among vulnerable women in similar settings. Therefore, in this study, we aimed to assess the association of integrated nutrition-sensitive interventions on household food security and dietary diversity among vulnerable women residing in the host communities of Cox’s Bazar, Bangladesh. We believe that our study will provide evidence for scaling up nutrition-sensitive interventions for vulnerable women living in low- and middle-income countries.

## Materials and methods

### Intervention description

From 2021 to 2024, the EU and UNICEF, in partnership with other partners, including the Government of Bangladesh, implemented integrated nutrition-sensitive interventions to enhance food security, dietary diversity, and economic resilience among vulnerable women-headed households in host communities in Cox’s Bazar. These interventions included two IGA (backyard poultry rearing and tailoring) activities along with IGA-related asset transfers, direct cash support, and provision of nutrition education and linking nutrition-specific services (such as linking beneficiaries to nearest community clinics or health facilities for growth monitoring and promotion (GMP) of under-five children, vitamin A supplementation, and deworming and iron folic acid (IFA) supplementation of pregnant women).

The intervention focused on vulnerable women-headed households residing in Teknaf, Pekua, Moheshkhali, and Cox’s Bazar Sadar. The criteria for selecting beneficiaries included: the household must be women-headed, no stable income or reliance on irregular/casual labor (earning less than Bangladeshi Taka (BDT) 10,000 or 118.18 USD (conversion rate: 1 USD = 84.62 BDT, January, 2021) per month); monthly household expenditure not exceeding BDT 2,000 (23.64 USD); ability to read and write (only applicable to those selected for tailoring); and residency in the selected upazilas for more than six months, with the intention to remain for the duration of the intervention. These selection criteria were developed and agreed upon by the Government (Director General for the Department of Agriculture Extension) and UNICEF; for the IGA component, the Ministry of Women and Children Affairs (MOWCA) criteria were used. For beneficiary selection, an inception meeting was conducted to inform the various local administrative stakeholders, such as *Upazila Nirbahi* Officer (UNO), *Upazila* Chairman, Livestock Officer/department head, etc. It also facilitated the selection of unions (the lowest administrative unit of the local government of Bangladesh) within the relevant *upazilas* for implementation of intervention. Community consultation meetings were organized to share the goals and objectives of the IGA interventions along with the beneficiary selection criteria. This was followed by a door-to-door household survey conducted by the community nutrition workers for beneficiary listing. Senior members of the CARE Bangladesh team (Project Manager IGA, Program Coordinator, Senior Project Officer) and UNICEF staff conducted spot checks during the door-to-door survey for beneficiary listing. A preliminary list of potential beneficiaries was generated based on the survey findings. The final selection was made in consultation with UNICEF, and the approved list was shared with local authorities.

A total of 2,000 vulnerable women were selected for the study. Of them, 1,600 were enrolled in backyard poultry rearing income-generating activities (IGA) based on the predefined criteria. The remaining 400 women who met these criteria and had minimum literacy and numeracy skills were enrolled in the tailoring training.

With technical support from the Food and Agriculture Organization (FAO), a structured backyard poultry rearing training module was developed, and 10 CARE Bangladesh staff members were trained to deliver the training to the 1,600 women. The beneficiaries of the backyard poultry rearing intervention received *Sonali* chickens/poultry, poultry sheds, feed, and essential veterinary supplies after completing their two-day backyard poultry rearing training. Local government officials, including representatives from the District Livestock Office (DLO) and *Upazila* Livestock Office (ULO), played a crucial role in supporting the implementation process.

UNICEF also hired trainers from an organization named Alliance for Cooperation and Legal Aid in Bangladesh (ACLAB) to provide tailoring training to 400 women. The tailoring beneficiaries underwent a one-month tailoring skill development training, after which they received sewing machines and essential materials to establish small-scale tailoring businesses in their communities. UNICEF also facilitated cash transfers to 2000 beneficiaries through an electronic beneficiary payment system. Each beneficiary received BDT of 30,000 (USD 244.27, January 2021) in six installments over a six-month period. These supports, including cash/assets/training, were provided in different months and years according to upazilas, that is in the same upazila, all beneficiaries received support on the same day. Trained personnel from CARE Bangladesh and UNICEF staff provided ongoing supervision, mentorship, market linkage support, and financial monitoring through regular follow-up and group sessions to ensure the quality implementation of the interventions. Moreover, through the UNICEF HOPE system, cash was distributed among the beneficiaries. A summary of the interventions is provided in S1 Table.

A conceptual framework (Fig 1) was developed to illustrate the hypothesized pathways linking integrated interventions to intermediate and final outcomes.

**Fig 1:**
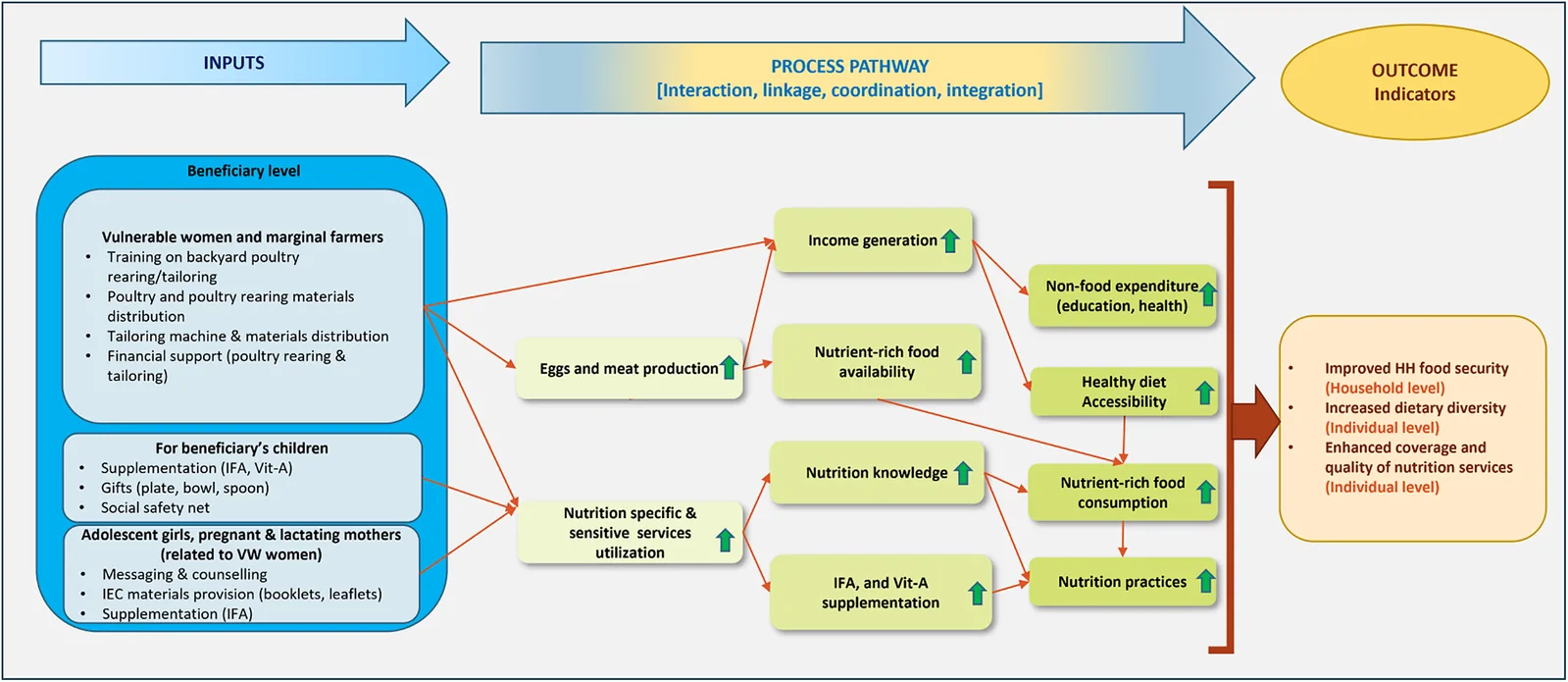
Conceptual framework of the integrated nutrition-sensitive intervention and its pathways to food security and dietary diversity.

### Study design and sites

We conducted repeated cross-sectional surveys as part of a larger mixed-method study for process documentation and pre-post comparison evaluation. The survey was conducted in two rounds: the first round of data collection took place from August 21, 2022, to March 21, 2023, and the second round from March 2 to June 30, 2024. The study was conducted in four upazilas of Cox’s Bazar District: Teknaf, Pekua, Moheshkhali, and Cox’s Bazar Sadar, where the nutrition-sensitive IGA interventions were implemented. These interventions were implemented in four unions in Pekua, two in Teknaf, two in Moheshkhali, and three in Cox’s Bazar Sadar. These locations were selected based on their remoteness and vulnerability, and to avoid duplication of activities implemented by other organizations.

### Sample size and sampling techniques

The sample size was calculated using the single-population proportion formula,


$$\begin{array}{c} n=Z^{\text{2}}p\;(\text{1}-p)/d^{\text{2}} \end{array}$$

assuming prevalence, p = 0.5; probability of type I error, α = 0.05; a 95% confidence level, z = 1.96; margin of error, d = 0.05; design effect = 1.5; and non-response rate = 0.05. A prevalence of 50% was used because no reliable prior estimate was available for the primary outcome in the study population, and this value provides the maximum sample size. The total sample was proportionally distributed across the four upazilas based on the number of eligible beneficiaries in each area. Participants in each survey round were selected using a simple random sampling method. Sampling was not stratified by intervention type. Based on these parameters, the required sample size was 607 vulnerable women; however, slightly more (first round n = 638, second round n = 617) respondents were interviewed in each round to ensure adequate representation. The two rounds were based on independent samples, and our study was not designed to follow the same individuals over time. Beneficiaries in each round were selected separately according to the study sampling framework.

CARE Bangladesh provided a list of 2,000 IGA beneficiaries comprising 1,600 backyard poultry rearing beneficiaries and 400 tailoring beneficiaries, from which we randomly selected participants for each round of the survey. In the first round (2022), we interviewed 638 of the 2,000 IGA beneficiaries (504 of the 1,600 from backyard poultry rearing and 134 of the 400 from tailoring); in the second round (2024), we interviewed 617 of the 2,000 IGA beneficiaries (490 out of 1,600 from backyard poultry rearing and 127 of the 400 from tailoring).

### Data collection and quality control

We collected data through face-to-face interviews using the same structured questionnaire in two separate rounds. We used the same list of beneficiaries for both rounds; however, participants were randomly selected independently for each round. A semi-structured questionnaire was used to collect data from vulnerable women. The questionnaire included several modules, including socioeconomic characteristics, dietary diversity, access to essential nutritional services, nutrition-related knowledge among vulnerable women, participation in nutrition-specific interventions or activities, and access to nutrition-sensitive information and services. The questionnaire was field-tested and modified, and the research assistants were retrained based on the field-tested findings. We collected data using tablet computers through the KoboToolbox. To maintain data quality, field supervisors observed 5% of the interviews and re-interviewed 5% of the randomly selected respondents. New interviews were conducted only when discrepancies exceeded 25%. In this study, discrepancies did not exceed 12%. We also ensured data quality through periodic analyses and any identified inconsistencies were reviewed and resolved through discussions with research assistants and respondents within 48 hours of data collection.

### Outcome variables

We examined two primary outcome variables: household food insecurity and women’s dietary diversity (DD), because they represent two complementary dimensions of food and nutrition security. Household food insecurity reflects access to food at the household level, whereas women’s dietary diversity reflects the quality and variety of food consumed at the individual level. Assessing both outcomes provides a more comprehensive understanding of the nutritional situation among vulnerable women. We used the Household Food Insecurity Access Scale (HFIAS) to assess the food security status of IGA beneficiary households over the past four weeks [31]. The HFIAS captures behavioral and psychological responses of households to food insecurity through nine occurrence questions, each with frequency options (rarely, sometimes, often), generating a total HFIAS score of 0–27. Based on the HFIAS prevalence indicator, we classified households into four levels: food-secure, mildly, moderately, and severely food-insecure. For the regression analysis, we recategorized them into two groups: 0 for moderately to severely food insecure as “food insecure”, and 1 for food secure to mildly food insecure as “food secure”, which are simpler and policy-relevant contrasts [32,33].

We used the Minimum Dietary Diversity for Women of reproductive age (MDD-W) to assess dietary diversity (DD) among vulnerable women [34,35]. We adopted the MDD-W questionnaire to collect dietary data from vulnerable women using a list-based recall method. The questionnaire included 24 food categories consumed during the previous 24 hours. The food categories were clustered into ten groups for analysis: (1) grains, white roots, tubers, and plantains; (2) pulses, beans, peas, and lentils; (3) nuts and seeds; (4) dairy; (5) meat, poultry, and fish; (6) eggs; (7) dark green leafy vegetables; (8) other vitamin A-rich fruits and vegetables; (9) other vegetables; and (10) other fruits [34,36]. A vulnerable woman was categorized as having inadequate DD if she consumed foods from less than five or fewer of these ten groups in the last 24 hours. “Dietary diversity” takes the value 0 for inadequate DD and 1 otherwise.

### Explanatory variables (covariates)

The covariates selected for the model included individual-level (age, education, marital status, and occupation) and household-level (income and wealth quintile) factors. Additionally, we incorporated an intervention-specific variable, such as the type of IGA assistance received (backyard poultry rearing or tailoring). Because of the small sample sizes in individual IGA categories, the type of IGA assistance variable was combined into broader categories and included in the models. Backyard poultry rearing and tailoring were combined into a single intervention category for analysis because both were implemented as home-based income-generating activities within the same program framework and included similar support components, including asset transfers, direct cash support, and nutrition education. The upazila (study area) was also used as an explanatory variable to account for geographical factors that might be associated with the program. These covariates were selected based on the program implementation manual, relevant literature [37,38], and contextual factors. Table 1 presents descriptions of the variables and their measurement methods.

**Table 1:** Variable description and measurement.

Variable	Type	Measurement
**Dependent variable**		
Household food security	Dummy	0 if moderate to severe food insecure, 1 secure to mild food insecure
Dietary diversity	Dummy	0 if inadequate dietary diversity (<5 food groups intake), 1 adequate dietary diversity (≥5 food groups intake)
**Explanatory variables**		
Upazila / study area	Categorical	Four categories: Moheshkhali, Pekua, Sadar, and Teknaf
Age of vulnerable women	Continuous	Age of vulnerable women in years
Marital status of vulnerable women	Categorical	Two categories: Married, and other (widowed/divorced/separated/never married)
Education of vulnerable women	Categorical	Two categories: No education and (≥1 year) of schooling
Occupation of vulnerable women	Categorical	Three categories: Day laborer, homemaker, and others (business, farmer)
Type of IGA assistance	Categorical	Two categories: Backyard poultry rearing, and tailoring
Household income	Continuous	Average monthly income (in BDT) of vulnerable women/household head
Wealth quintile	Categorical	Five categories: Poorest, poorer, middle, richer, and richest

### Statistical analysis

We analyzed the data using the statistical programming language R (R Core Team, 2024; version 4.4.1). Descriptive analysis included means and percentages to summarize the respondents’ socioeconomic characteristics. We also conducted a propensity score-based analysis to evaluate the association of the intervention by comparing the prevalence of food insecurity and dietary diversity, using two rounds of data collected in 2022 and 2024. The time variable served as a proxy for nutrition-sensitive IGA interventions provided over the years 2022–2024. The two rounds were conducted in different calendar periods, and the dataset did not include direct measures of season, month of data collection, local food prices, or other contextual indicators. Therefore, temporal confounding from seasonal food availability and broader economic changes could not be explicitly adjusted for in the models. Due to the limited sample size within each intervention group, we were unable to independently examine the distinct associations of backyard poultry rearing and tailoring interventions on food security and dietary diversity outcomes separately. Our analysis estimates changes in outcome prevalence and associated factors among program beneficiaries between the two survey rounds after adjusting for measured covariates, rather than an average treatment effect or a causal program effect.

### Propensity score weighting analysis

We considered food security and dietary diversity as responses. Both variables were defined as binary, with food insecurity and inadequate dietary diversity considered as reference categories. Age, marital status, education level, occupation, household income, type of IGA assistance, wealth quintile, and upazila/study area were considered as predictors in the propensity score model.

To obtain the estimate of the association between intervention (survey year) and outcome (dietary diversity or food insecurity), we considered both binomial and modified Poisson regression models [39]. The models were fitted with a pseudo-population, obtained with the inverse propensity score weights (IPSW), which was the reciprocal of the probability of receiving the treatment and control for the treatment (year 2024) and control (year 2022) group, respectively. The probability of receiving the treatment, also known as the propensity score, was estimated from a logistic regression model with treatment (survey year) as the outcome and measured covariates as the predictors. Because the distributions of covariates in the pseudo-population were balanced between the two treatment levels, the treatment factor was considered as the only predictor for the binomial and modified Poisson regression models.

To examine whether the estimated association between the intervention and outcomes is sensitive to unmeasured confounders, we reported E-value (in RR scale) and corresponding 95% confidence intervals for all IPSW-based estimates. A large E-value indicates that the estimated associations are robust against unmeasured confounders [39].

### Ethical considerations

Ethical approval for the study was obtained from the Institutional Review Board (IRB) of the BRAC James P Grant School of Public Health, BRAC University, Dhaka, Bangladesh (reference number: IRB-12 June ‘22−020). Written informed consent was obtained from the participants and household heads of the host community.

## Results

### Households’ food security status

A significant improvement in food security status was observed among vulnerable women between 2022 and 2024 (Fig 2). The prevalence of household food security among vulnerable women showed a substantial increase, rising from 9% in 2022 to 34% in 2024. The proportion of households with mild food insecurity remained the same at 15%. The prevalence of moderate food insecurity decreased by four percentage points. Notably, the proportion of households with severe food insecurity declined from 58% in 2022 to 37% in 2024.

**Fig 2:**
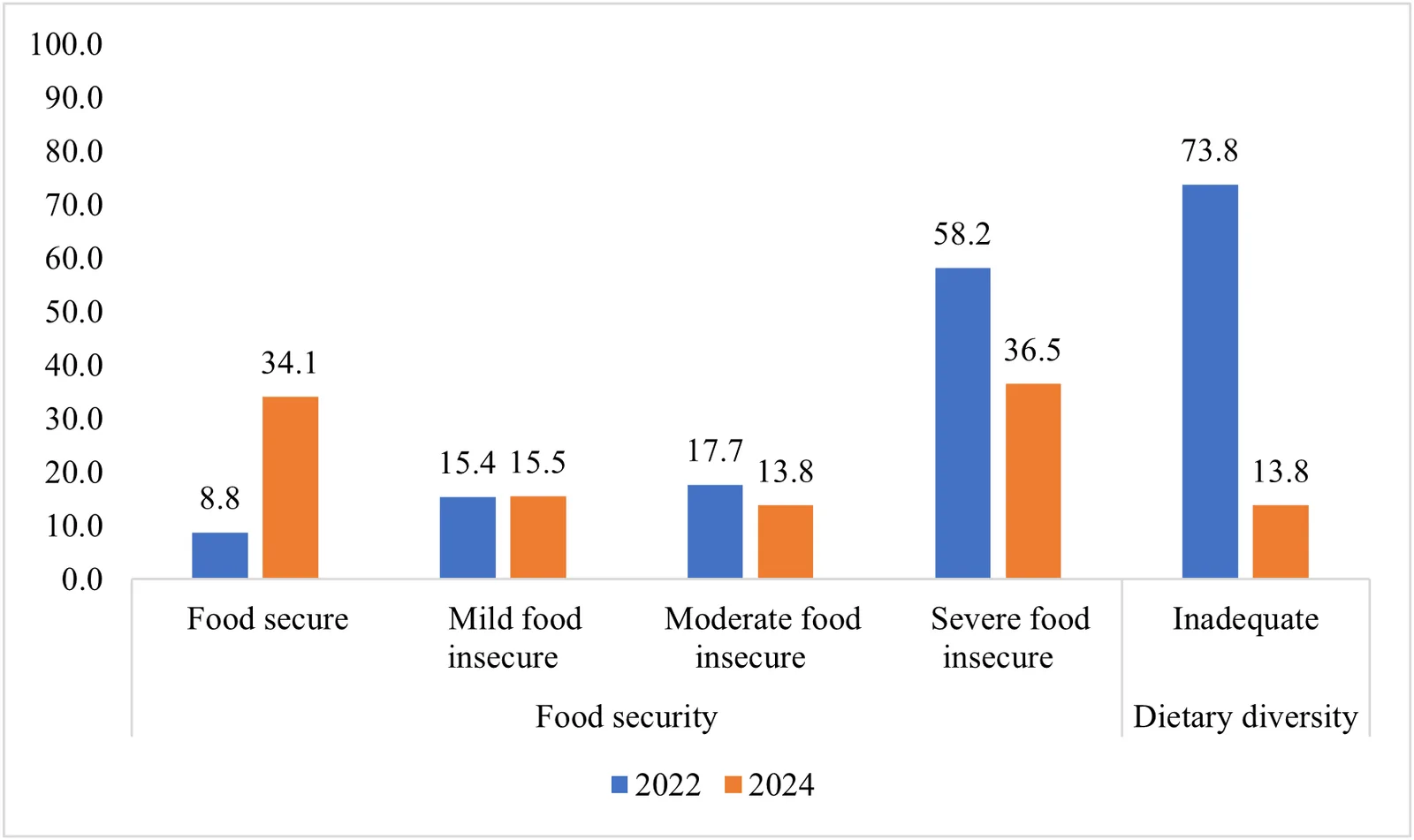
Observed changes in percentage household food security status and dietary diversity of vulnerable women in 2022 and 2024.

### Vulnerable women’s dietary diversity

Fig 3 also shows changes in the dietary diversity of vulnerable women between 2022 and 2024. The prevalence of adequate DD (≥5 food groups) in vulnerable women increased by 60 percentage points from 2022 to 2024. Conversely, inadequate DD was more prevalent in 2022 (74%) but decreased sharply to 14% in 2024.

**Fig 3:**
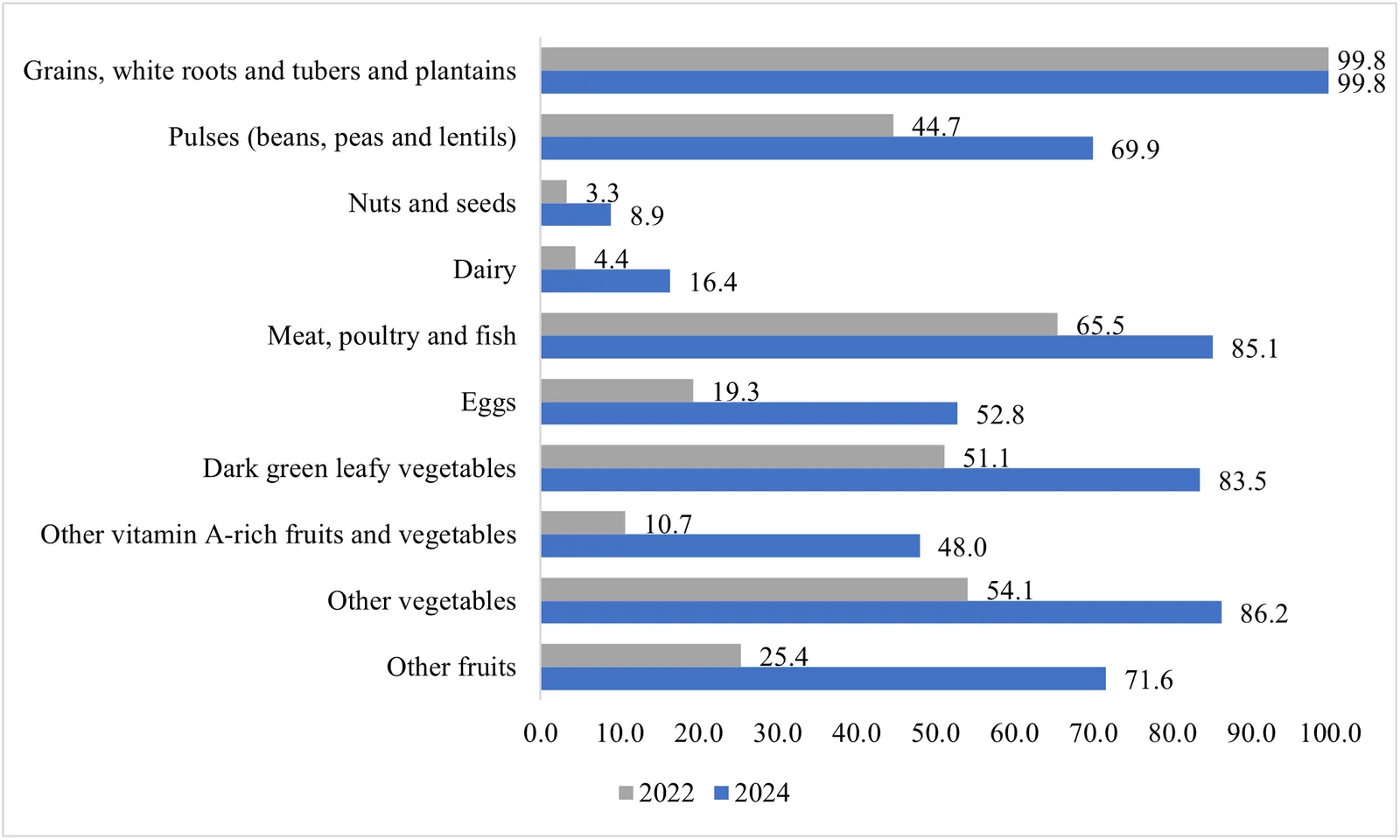
Comparison of 10 food group consumption among vulnerable women in 2022 and 2024. **Note:** Percentages are unweighted; values are presented as proportions of the samples.

### Consumption of specific food groups among vulnerable women

Fig 3 illustrates the percentage of vulnerable women who consumed 10 food groups over a 24-hour recall period in 2022 and 2024. A notable improvement in consumption was observed over the time across almost all food groups except one group (grains, white roots and tubers, and plantains). The highest change was observed in the consumption of other fruits groups, with a 46.2% points increase in 2024 compared to 2022. Significant percentage point increases were also noted for other vitamin A-rich fruits and vegetables (+37.3%), eggs (+33.5%), dark green-leafy vegetables (+32.4%), and other vegetables (+32.1%) groups in 2024.

### Checking the covariate balance between the two rounds of survey

Table 2 compares the distribution of different covariates between the two independent groups surveyed in 2022 and 2024, both before and after inverse propensity score weighting (i.e., in pseudo-population). Before assigning weight, notable differences were observed in marital status, education, occupation, and household income. However, after assigning weight, balance improved, with standardized differences reduced across most variables.

**Table 2:** Weighted differences in outcome prevalence (in %) between 2022 and 2024.

Variables	Unweighted		Weighted	
	2022	2024	2022	2024
**Age of vulnerable women (mean)**	41.6	41.8	41.7	41.8
**Marital status of vulnerable women (married)**	8.9	12.6	10.8	11.6
**Education level of vulnerable women (≥1 years)**	27.9	32.7	30.8	30.9
**Household income (mean)**	6,152.7	9,554.3	8,369.5	7,901.5
**Type of IGA assistance (backyard poultry rearing)**	79.0	79.4	77.3	78.9
**Occupation of vulnerable women**				
Day labor	27.3	26.1	24.3	25.5
Homemaker	33.1	18.2	25.3	25.7
Others (business, farmer)	39.7	55.8	50.4	48.8
**Wealth quintile**				
First quintile	20.1	20.1	21.6	20.5
Second quintile	20.1	19.9	19.1	19.5
Third quintile	19.9	20.1	18.3	19.9
Fourth quintile	20.1	19.9	19.0	19.8
Fifth quintile	19.9	19.9	22.0	20.3
**Upazila**				
Moheshkhali	35.4	34.8	31.5	30.9
Pekua	29.5	30.3	32.0	31.5
Cox’s Bazar Sadar	15.7	14.9	15.3	16.5
Teknaf	19.4	19.9	21.2	21.0

The difference in the proportion of married individuals between the two time periods was approximately 4% before assigning weights, which decreased to less than 1% after assigning weight assignment. The low percentage of married women in the sample is consistent with the intervention’s eligibility criteria, which focused on economically vulnerable women from households with no regular income or reliance on irregular or casual labor. The percentage of vulnerable women with one or more years of education was higher in 2024 (32.7%) than in 2022 (27.9%). After assigning weights, this difference was reduced (30.8% vs. 30.9%, respectively). Among the three different types of occupations, the percentage of day laborers remained similar, but homemakers and others (business or farming) changed after weighting. The proportion of women engaged in business or farming was higher (55.8% vs. 39.7%) in 2024 than in 2022 but became more balanced (50.4% vs. 48.8%) after weighting the data. Before assigning weights, the mean income was notably higher in 2024 (9,554.3 BDT) than in 2022 (6152.7 BDT), and after weighting, it reduced from 8,369.5 BDT to 7,901.5 BDT. In contrast, the age of vulnerable women, type of IGA assistance, and household wealth quintile remained balanced before and after weighting.

Fig 4 (love plot) shows the standardized mean differences (SMDs) for comparing covariate balance between two treatment groups. The red points indicate the SMDs in covariate balance before and after inverse probability weighting. After inverse probability weighting, all absolute standardized mean differences were below 0.10, indicating adequate balance across the measured covariates. This suggests that the weighting procedure successfully reduced baseline covariate differences and improved comparability between groups before estimating the associations on food security and dietary diversity.

**Fig 4:**
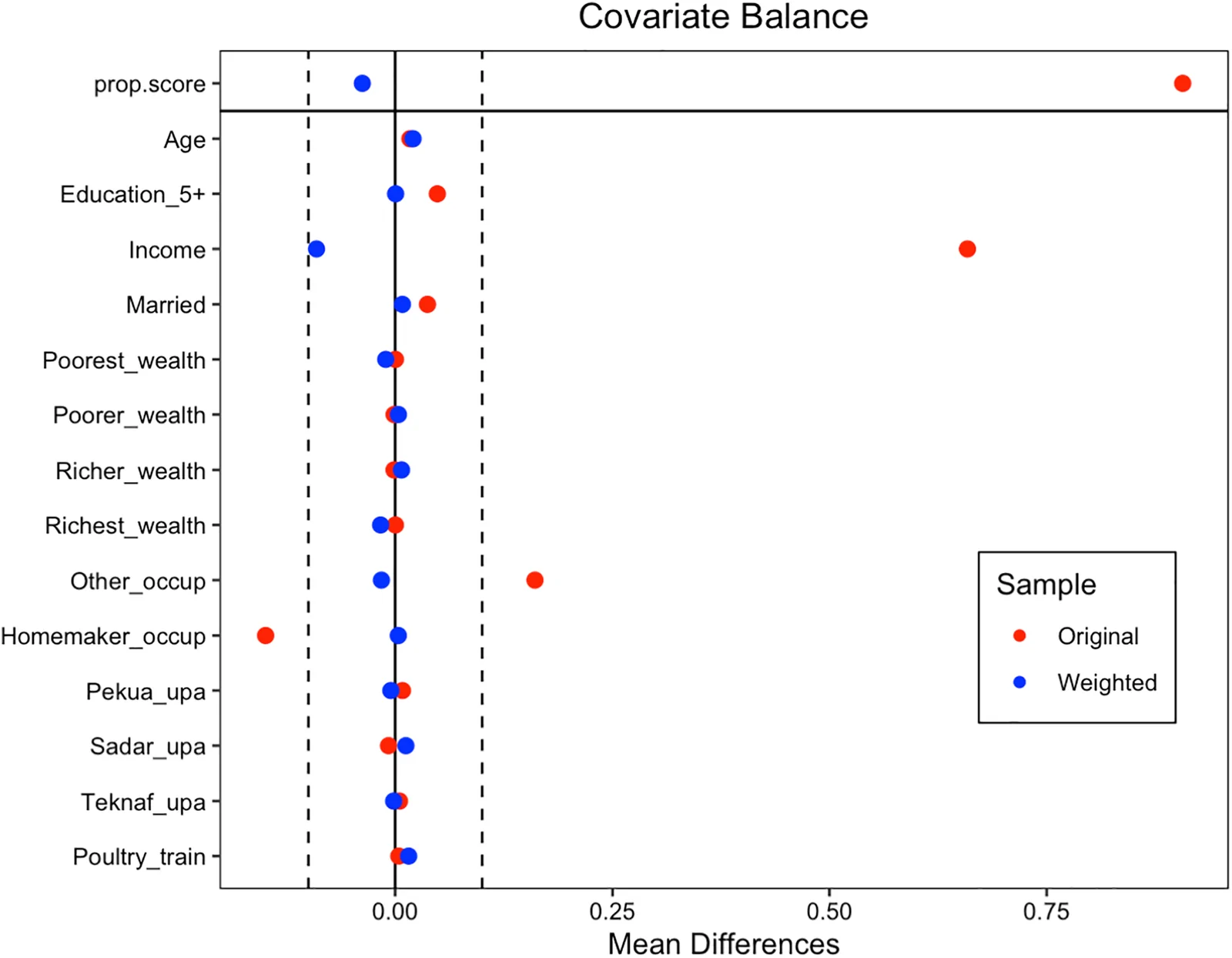
Standardized Mean Differences of Covariates Comparison.

Table 3 presents the distribution of propensity scores between the two groups in 2022 and 2024, which shows that the distributions of propensity scores overlap well over the survey years 2022 and 2024. This shows that for a given propensity score, there is a non-zero probability of receiving both levels of intervention, although they are different in lower and higher PS values.

**Table 3:** Comparison between two groups (2022 vs 2024) by the PS-score distributions.

	Survey round	
PS-categories	2022 [n (%)]	2024 [n (%)]
[0.0,0.2]	78 (12.2)	13 (2.1)
[0.2,0.4]	252 (39.5)	109 (17.7)
[0.4,0.6]	207 (32.4)	209 (33.9)
[0.6,0.8]	81 (12.7)	199 (32.3)
[0.8,1.0]	20 (3.1)	87 (14.1)

### Estimate of average differences between survey rounds

Table 4 presents a comparison of the prevalence of food security and dietary diversity between 2024 and 2022, after adjusting for baseline covariates. The adjusted associations were estimated using modified Poisson and binomial regression models with and without the weighted data. The results showed a significant difference in outcome prevalence between the 2022 and 2024 survey rounds after propensity score weighting. According to the IPSW-Poisson approach, in 2024, the vulnerable women had a 53% higher (RR: 1.53, 95% CI: 1.27–1.85, p < 0.001) likelihood of household food security (mildly insecure to food-secure) compared to 2022. The unweighted modified Poisson regression model (RR: 2.05, 95% CI: 1.75–2.40, p < 0.001) showed a stronger association. It is pertinent to note that the estimate from the unweighted modified Poisson regression model reflect the conditional associations, whereas those obtained from the IPSW reflects marginal associations. The weighted binomial model estimated a 0.16 higher probability of household food security in 2024 compared to 2022 (95% CI: 0.09–0.22, p < 0.001), corresponding to a 16-percentage-point difference. In the unweighted binomial model, the estimated increase was larger at 0.25, indicating a 25% improvement in food security (95% CI: 0.20–0.30, p < 0.001). The E-value for this model was 2.44, which states that an unmeasured confounder would need to be strongly associated with the response and the intervention to nullify the observed measure of association, which was 1.53 in RR scale.

**Table 4:** Estimate of the adjusted association of food security and dietary diversity with survey years, using modified Poisson and binomial regression models, and corresponding IPSW methods.

Outcome	Method (parameter)	E-value (95% CI)	Estimate	95% CI	p-value
Food security	IPSW-Poisson (RR)	2.44 (1.86, 3.11)	1.53	1.27, 1.85	**<0.001**
	Poisson regression (RR)		2.05	1.75, 2.40	**<0.001**
	IPSW-Binomial (RD)	2.44 (1.71, 3.32)	0.16	0.09, 0.22	**<0.001**
	Binomial (RD)		0.25	0.20, 0.30	**<0.001**
Dietary diversity	IPSW-Poisson (RR)	5.00 (4.15, 5.99)	2.78	2.36, 3.27	**<0.001**
	Poisson regression (RR)		3.29	2.88, 3.77	**<0.001**
	IPSW-Binomial (RD)	5.00 (4.07, 6.11)	0.53	0.47, 0.59	**<0.001**
	Binomial (RD)		0.60	0.56, 0.64	**<0.001**

The dietary diversity of vulnerable women has increased from 2022 to 2024. In the IPSW-Poisson approach, the likelihood of increased dietary diversity was slightly two and a half times higher (RR: 2.78, 95% CI: 2.36–3.27, p < 0.001) in 2024 than in 2022. The unweighted modified Poisson regression model (RR: 3.29, 95% CI: 2.88–3.77, p < 0.001) showed a stronger association with dietary diversity. The weighted binomial model showed a 0.53 increase in the probability of achieving dietary diversity, equivalent to a 53 percentage-point improvement after weighting adjustment (95% CI: 0.47–0.59, p < 0.001). The unweighted binomial model estimated a slightly higher increase of 0.60, indicating a 60 percentage-point improvement in dietary diversity (95% CI: 0.56–0.64, p < 0.001). For this model, the E-value is 5.00, which states that the observed estimate of association 2.78 is robust against unmeasured confounders.

## Discussion

In this study, we found that integrated nutrition-sensitive interventions (backyard poultry rearing and tailoring) combined with cash transfers and nutrition education were associated with significant improvements in household food security and dietary diversity among vulnerable women in the host community of Cox’s Bazar, Bangladesh. Despite signs of a decline in food security in recent years in Bangladesh [40], we found improvements in the households of participants who received backyard poultry rearing or tailoring interventions over time. Between 2018 and 2023, the South Asian Network on Economic Modeling (SANEM) conducted two nationally representative surveys using the Food Insecurity Experience Scale (FIES) following FAO's 2022 guidelines, which showed a deterioration in food security among both poor and non-poor households [40]. Moreover, in Bangladesh, there has been a small change in household dietary diversity in recent years [41]. While multiple studies in Bangladesh reported persistently low dietary diversity among women [42–44], we observed a significant improvement in adequate dietary diversity among vulnerable women, from 26% in 2022 to 86% in 2024. In 2023, Bangladesh was listed among the ten largest countries most severely impacted by the food crisis [45]. Therefore, these positive changes in our outcomes can be considered evidence for scaling up nutrition-sensitive IGA interventions.

After receiving the intervention, more than three-fourths of the vulnerable women consumed five or more food groups on average in the 24-hour period preceding the interview day. Vulnerable women received poultry (*Sonali* chickens) or tailoring training, along with direct asset transfers and cash support, enabling them to engage in IGAs and improve their financial capacity, thereby enhancing their access to food. Households with greater purchasing power may afford a more diverse range of foods, leading to an association with improved dietary diversity and reduced food insecurity. Previous studies have also suggested that small-scale, nutrition-sensitive interventions, such as backyard poultry rearing, can serve as a source of regular household income, directly improving food access [46–48]. Tailoring can also result in income generation and enhanced purchasing power, ultimately improving access to diverse and nutritious food items.

Vulnerable women receiving backyard poultry-rearing support were able to produce nutrient-rich foods, such as eggs or meat, at the household level after receiving the intervention. This production enhances food availability and ensures better access to animal-source foods. Additionally, surplus production was sold, generating income that directed beneficiaries to purchase other nutrient-dense foods, further diversifying the household diet. Over time, this dual pathway through production and income generation has created more sustainable and stable access to diverse and nutritious diets. Tailoring can boost income, provide access to markets, and leverage social networks. These attributes can contribute to improvements in household food security and dietary diversity.

Access to nutritional education and counseling likely improves dietary practices by enhancing nutritional knowledge. Habtu et al. showed that nutrition-specific components of integrated interventions improved beneficiaries’ dietary diversity by addressing the immediate causes of undernutrition [49]. The FAO reported that a combination of livelihood training, such as tailoring with nutritional education, improved dietary diversity scores in Kenya [50]. In 2013, Ruel and Alderman mentioned that a combination of nutrition-specific and nutrition-sensitive interventions is required to improve maternal and child nutrition [51].

In our study, we used rigorous analytical methods, including modified Poisson regression and, inverse propensity score weighting, to measure the association between the intervention and the outcomes (food security and dietary diversity) separately. Consistent results from these methods provide a positive association between integrated nutrition-sensitive interventions and food security and dietary diversity. Multifaceted interventions (training in IGAs, such as backyard poultry rearing and tailoring, along with access to nutrition-specific services and education) might have worked through various interconnected pathways to improve household food security and the dietary diversity of vulnerable women. Our findings align with those of previous studies, such as a review by Ruel et al., who found that integrating multiple interventions, for example, a combination of agriculture and nutrition education interventions, leads to greater dietary improvements than standalone interventions [26].

Our study has several strengths too. We used a simple random sampling process to identify the study participants to reduce selection bias and strengthen the representativeness of the sample. Moreover, we applied inverse propensity score weighting as an alternative approach for this observational analysis. This method was used to improve balance in observed covariates between groups before estimating the association between intervention status and the outcomes. However, like other observational methods, it depends on measured covariates and does not completely eliminate the possibility of bias.

One limitation of our study is that it was based on two repeated cross-sectional survey rounds without random assignment or a concurrent control group; therefore, residual confounding and other time-varying contextual factors could not be excluded. Therefore, the observed changes were interpreted as associations rather than definitive causal effects of the intervention. Although propensity score weighting improved the balance of observed characteristics between beneficiaries in the two survey rounds, residual confounding from unmeasured time-varying factors may still remain. One limitation of this study is that the two survey rounds were conducted at different times, while key outcome measures were assessed using short recall periods, which may have been affected by recent conditions and contextual factors. Seasonal variation in food access, food prices, dietary patterns, climatic shocks, and fluctuations in local economic and agricultural conditions, government support, or the activities of other development actors may have influenced the observed differences. As these contextual variables were not available in the dataset, the observed differences could be interpreted as attributable solely to the intervention. Future research should focus on establishing causality. Although the analysis was adjusted for time effects, the intervention rollout was not uniform; therefore, exposure to the intervention was variable. Additionally, this study was nested within a broader process documentation and pre-post comparison; therefore, it did not fully adhere to a rigorous impact evaluation design. Finally, due to the limited sample size across intervention arms, we were unable to independently assess the differential associations of backyard poultry rearing and tailoring interventions on food security and dietary diversity outcomes.

Our findings support the need for multi-sectoral, integrated nutrition-sensitive interventions to achieve significant improvements in household food security and dietary diversity of vulnerable women. These improvements highlight the importance of localized, structured, well-targeted, and integrated interventions that address both the underlying determinants and immediate causes of food security and dietary diversity [52]. These results are consistent with previous evidence that integrated programs combining livelihood support and nutritional education are more effective than standalone interventions in achieving sustainable nutritional outcomes [26,53]. However, before scaling up integrated interventions, several factors should be considered, including effectiveness, relevance, feasibility, political support, local capacities, and cost-effectiveness [2,54]. These factors are essential for ensuring the long-term success and sustainability of such interventions. Based on the evidence from this study, it can be suggested that there is an urgent need to scale up these integrated interventions to improve food security and dietary diversity in vulnerable population groups in Bangladesh and other similar settings.

## Conclusion

In this repeated cross-sectional study, household food security and dietary diversity were substantially higher in 2024 than in 2022 among vulnerable women in the study areas. These differences remained after propensity score weighting, indicating a positive association between program implementation and the study outcomes. However, because the study was non-randomized and based on two cross-sectional survey rounds, residual confounding and other time-varying contextual factors cannot be excluded. Therefore, the observed differences should not be interpreted as definitive causal effects of the intervention. Future controlled or longitudinal evaluations are needed to establish the causal effectiveness and long-term sustainability of integrated nutrition-sensitive interventions.

## Supporting information

S1 Table Summary of the intervention. (DOCX)

S1 Data Dataset in CSV format. (CSV)

S1 File Questionnaire in English Language. (PDF)

S2 File Questionnaire in Bengali Language. (PDF)

S3 File STROBE Checklist. (DOC)

S4 File TIDieR Checklist. (DOCX)
