# Supplementary material for: Integrated nutrition-sensitive interventions are associated with improvements in household food security and dietary diversity among vulnerable women in the host communities of Cox’s Bazar, Bangladesh: Findings from a pre-post comparison study
Source: PLoS One. 2026 Nov 5;21(11):e0362124. doi: 10.1371/journal.pone.0362124 (PMC13739155; doi:10.1371/journal.pone.0362124)
Supplement: Supplementary file 1 [file pone.0362124.s003.pdf]

The Project is funded by the European Union

Module 1.1: Household Head Module

Section 1: General Information and Household Socio-Economic Status

Number	Variable	Question	Response		Code	Instructions
102	HS_102	Upazilla name and code	___________<br>|____|____|			
110	SE_110	What type of cooking fuel does your household mainly use?	1 = Electricity<br>2 = LPG<br>3 = Piped Natural Gas<br>4 = Kerosene<br>5 = Coal/Lignite<br>6 = Charcoal<br>7 = Wood<br>8 = Straw/grass/agri waste/leaves<br>9 = Animal dung<br>10 = Biogas<br>99 = Other (specify) ………………			If it is '1-4' Go to 111.
111		Do you currently have pets or birds in your household? If so, how many?				
			A.	B.		
			1 = Yes 2 = No	Number (if yes)		If the number is more than 95, code 95
	SE_111A	Cow/Buffalo	1 = Yes 2 = No	|____|____|		
	SE_111B	Sheep/Goat/Pig	1 = Yes 2 = No	|____|____|		
	SE_111C	Chicken/Duck/Swan	1 = Yes 2 = No	|____|____|		
	SE_111D	Small animals/birds (e.g. rabbits, pigeons, etc.)	1 = Yes 2 = No	|____|____|		
	SE_111E	Are there other animals?				
	SE_111ET	Other (specify) ———	1 = Yes 2 = No	|____|____|		
111	SE_111F	Does your household have fishpond?	1 = Yes 2 = No			
111	SE_111G	Does your household have homestead garden?	1 = Yes 2 = No			
112		Which of the following items are active in your kitchen?				
	SE_112A	Electricity connection	1 = Yes 2 = No			

Number	Variable	Question	Response	Code	Instructions
	SE_112B	Solar Power	1 = Yes 2 = No		
	SE_11C	Radio	1 = Yes 2 = No		
	SE_112CA	Television	1 = Yes 2 = No		
	SE_112D	Mobile Phone	1 = Yes 2 = No		
	SE_112DA	Telephone	1 = Yes 2 = No		
	SE_112D2	Smart Phone	1 = Yes 2 = No		
	SE_112DB	Computer/Laptop	1 = Yes 2 = No		
	SE_112DC	DVD/VCD Player	1 = Yes 2 = No		
	SE_112E	Fan	1 = Yes 2 = No		
	SE_112F	Cot/Chowki	1 = Yes 2 = No		
	SE_112G	Almira/Warrob/Showcase	1 = Yes 2 = No		
	SE_112H	Refrigerator/Fridge	1 = Yes 2 = No		
	SE_112HA	Air Conditioner	1 = Yes 2 = No		
	SE_112I	Table/Chair	1 = Yes 2 = No		
	SE_112J	Clock/wall clock	1 = Yes 2 = No		
	SE_112K	Bicycle	1 = Yes 2 = No		
	SE_112L	Motorcycle/Scooter	1 = Yes 2 = No		
	SE_112LA	Autobike/Tempo/CNG	1 = Yes 2 = No		
	SE_112M	Animal Driven Cars	1 = Yes 2 = No		
	SE_112N	Car/Truck	1 = Yes 2 = No		
	SE_112O	Boat	1 = Yes 2 = No		
	SE_112P	Engine Boat	1 = Yes 2 = No		
	SE_112Q	Rickshaw/Van	1 = Yes 2 = No		
	SE_112R	Power Tiller	1 = Yes 2 = No		
	SE_112S	Shallow Machine/Water Pump	1 = Yes 2 = No		
	SE_112T	Plough	1 = Yes 2 = No		
	SE_112U	IPS/Electric Generator	1 = Yes 2 = No		
	SE_112V	Fishing net	1 = Yes 2 = No		
	SE_112W	Sewing Machine	1 = Yes 2 = No		
	SE_112X	Microwave/Electric Oven	1 = Yes 2 = No		
113	SE_113	What main material is used to build the exterior wall of your main living house? (Observe and record)	**The Normal Walls:**<br>1 = No walls<br>2 = Cane, sugarcane leaves/palm leaves/tree trunks/bamboo<br>3 = Dirt<br>**Early Stage Walls:**<br>4 = Bamboo with mud<br>5 = Stone with mud<br>6 = Plywood<br>7 = Cardboard/Card Board/Polyethylene<br>**Full Walls:**<br>8 = Tin<br>9 = Brick-cement<br>10 = Stone & Lime / Cement<br>11 = Wooden plank/ blade<br>12 = Brick<br>13 = Cement block<br>99 = Other (specify)...............		Observe and record

The Project is funded by the European Union

Number	Variable	Question	Response	Code	Instructions
114	SE_114	What materials are used to build the roof of your main living house?<br>(Observe and record)	**Normal Roof:**<br>1= No roof<br>2 = Straw / Palm Leaf / Round Leaf etc.<br>**Raw Roof:**<br>3= Bamboo and soil<br>4= Wooden plank<br>5= Pitch Board/ Polyethylene/Card Board<br><br>**Full Roof:**<br>6= Tin<br>7= Wood<br>8= Ceramic Tiles/Tile<br>9= Brick-Cement<br>10= Wooden blade<br>99= Other (specify)		Observe and record
115	SE_115	What materials are used to build the floor of your main living house?<br>(Observe and record)	**Raw flooring:**<br>1= Soil/Sand<br>**Elementary flooring:**<br>2= Wooden plank<br>3= Palm tree/Bamboo<br>**Full floor:**<br>4= Ceramic Tiles<br>5= Brick-cement<br>6= Mosaic<br>99= Other (specify)...............		Observe and record
116	SE_116	How many rooms for living in your household? (including rooms for home maid)	|______|______|		
122	SE_122	What is your average monthly household income? (Considerign the past 12 months)	|____|____|____|____|____ |____|____| Taka<br>88 = Refusal to inform		

Section 2: Household Water Supply, Sanitation and Sanitation

Number	Pointer	Question	Response	Code	Instructions
201	WH_201	What is the main source of drinking water for members of this household?	**Pipe water**<br>1 = Piped to dwelling<br>2 = Piped to yard/plot<br>3 = Public Tap<br>**Tubewell or pitpipe**<br>4 = Shared<br>5= Household<br>**Well water**<br>6 = Covered/Protected<br>7 = Uncovered/not protected<br>8 = Rain water<br>**Surface water**		

Number	Pointer	Question	Response	Code	Instructions
			9=Pond/River/Canal/Haor/Irrigation Channel<br>10 = Water tank<br>**Fountain water**<br>11 = Protected<br>12 = Unprotected<br>13 = Water tank truck<br>14 = Water tank van/small car<br>15 = Bottled water<br>99 = Other (specify)..........		
205	WH_205	What kind of toilet facility does this household use? (Check One)	1 = Connected to the sewerage line<br>2 = Toilet with septic tank<br>3 = Ring slab with water seal<br>4 = Ringslab without water seal<br>5 = Pit latrine with slab<br>6 = Pit latrine without slab<br>7 = Hanging toilet<br>8 = No toilet (road/riverside/open field/wherever/bush-forest)<br>99 = Other (specify).............................		Go to 207 if '8'.
206	WH_206	Is your toilet shared or a public toilet? (Regardless of where the toilet is located, the question has to be asked.)<br>Combined toilets: Toilets owned by a certain number of users.<br>Public toilets: Toilets that do not specify the number of users.)	1 = Own<br>2 = Jointly<br>3 = Public toilets<br>77 = Don't know		

Section 3A Food Safety and Adaptation Strategies

Now, I'm going to take some more time from you to ask you some questions about the food safety of your food. I would ask you to think back to the previous 4 weeks about Rarelyand Rarelyyou experienced food insecurity in your diet. Note that by Khana, I mean those members who usually share food from the same pot. If you have any questions, please stop and ask me.

Number	Sachak	Question	Response	Code	Instructions
301	FS_301	In the past four weeks, did you ever worry that your family would not have enough food due to lack of resources or money to buy food?	0 = Never<br>1 = Rarely (once or twice)<br>2 = Sometimes (3 to 10 times)<br>3 = Often (more than 10 times)		
302	FS_302	In the past four weeks, were you or any family members ever not able to eat the kinds of foods you preferred because of	0 = Never<br>1 = Rarely (once or twice)<br>2 = Sometimes (3 to 10 times)<br>3 = Often (more than 10 times)		

Number	Sachak	Question	Response	Code	Instructions
		a lack of resources or money to buy food?			
303	FS_303	In the last 4 weeks, Did you or any member of your household eat only rice due to lack of food (with salt, onions, chili etc.)	0 = Never<br>1 = Rarely (once or twice) if yes<br>2 = Sometimes (3 to 10 times)<br>3 = Often (more than 10 times)		
304	FS_304	In the last 4 weeks did you or any member of your family Had to eat any food that usually did not eat (such as wild/non food/ instead of rice any other food that usually does not eat) ?	0 = Never<br>1 = Rarely (once or twice)<br>2 = Sometimes (3 to 10 times)<br>3 = Often (more than 10 times)		
305	FS_305	In the past four weeks, did you or any household member ever have to eat a smaller meal at breakfast, lunch, or dinner than you felt you needed because there was not enough food for your family? not enough food or money to buy food?	0 = Never<br>1 = Rarely (once or twice<br>2 = Sometimes (3 to 10 times)<br>3 = Often (more than 10 times)		
306	FS_306	In the past four weeks, did you or any other household member ever have to eat fewer than three meals in a day because there were not enough resources or money to purchase food?	0 = Never<br>1 = Rarely (once or twice)<br>2 = Sometimes (3 to 10 times)<br>3 = Often (more than 10 times)		
307	FS_307	In the past four weeks, did you or any household member ever go to sleep at night hungry because there was not enough money to purchase food?	0 = Never<br>1 = Rarely (once or twice)<br>2 = Sometimes (3 to 10 times)<br>3 = Often (more than 10 times)		
308	FS_308	In the past four weeks, did you or any household member ever go to sleep at night hungry because there was not enough money to purchase food?	0 = Never<br>1 = Rarely (once or twice)<br>2 = Sometimes (3 to 10 times)<br>3 = Often (more than 10 times)		
309	FS_309	In the past four weeks, did you or any household member ever go a whole day and night without eating anything because there was not enough money to purchase food?	0 = Never<br>1 = Rarely (once or twice)<br>2 = Sometimes (3 to 10 times)<br>3 = Often (more than 10 times)		If '0', go to 310.

Module 1.6: Vulnerable Women module

Section 1: General Information

No	Variable	Question	Response Options	Code	Instructions
102	VGI _102A	Age of vulnerable women	A. |____|____| year		
103	VGI_103	What is your (vulnerable women) marital status?	1 = Never married<br>2 = Currently married<br>3 = Separated;<br>4 = Divorced<br>5 = Widowed;<br>88 = Refused		
104	VGI_104	In total, how many years have you spent at school and in full- time study (excluding pre-school)?<br><br>***[Instruction:** Write down in total, the years the respondent spent in school for formal education (Is completed years) [Excluding pre-school and informal education). Write down "00" if no formal education.]*	|__|__| Years<br><br>• Primary/Ebtedayee=5<br>• Secondary/Dakhil=10<br>• Higher secondary/Diploma/Alim=12<br>• Graduate/Fazil=16<br>• Post graduate=18		
105	VGI_105	What is your main occupation in the last 12 month?<br><br>***Instruction:***<br>*If the respondent involved more than one profession since last 12 months, then consider the main occupation which he spent most of the time and write down the Response code.*	1 = Agricultural work (paddy)<br>2 = Agricultural work (without paddy)<br>3 = Agricultural day labor<br>4 = Unskilled day labor<br>5 = Skilled day labor<br>6= Rickshaw puller/ van/wheel barrow/*baby taxi*/ Boatman<br>7=Fisherman<br>8=Employee<br>9=Professionals<br>10=Businessman<br>11= Petty businessman<br>12= House maid<br>13= Agricultural work (Zoom farmer)<br>14= Do not earn<br>15=Poultry/dairy farmer<br>16=Handicrafts<br>17 =Agricultural work (Vegetable grower)<br>18=Fish farming<br>19 = Housewife<br>20= Student<br>21= Immigrant<br>22 = Tailoring<br>77 = Don't know<br>99 = Others(specify bellow)<br>…………………………………		

Section 2: Dietary diversity of the vulnerable women

<table>
<tr><th>No</th><th>Variable</th><th colspan="2">Question</th><th>Yesterday during the day or night, did you consume the following food items? (A)</th><th>During the last 07 days how many days did you consume the following food items (B)</th><th>During the last 07 days how many times did you consume the following food items (C)</th></tr>
<tr><td colspan="7">Now I request you to describe the food that you have eaten at home and out of home during the day or night (yesterday morning 6.00 am to today morning 6.00 am) yesterday. Please tell all types of food, drinks that you have eaten with morning, noon and dinner, or have breakfast / light breakfast. Tell us by think if you ate any food while preparing food.
Get started with what you ate yesterday morning.
-What did you eat in the morning wake up? Did you eat anything more?
-What did you eat more in the morning? Did you eat anything more?
-What did you eat at noon? Did you eat anything more?
-What did you eat at afternoon? Did you eat anything more?
-What did you eat at evening? Did you eat anything more?
-What did you eat at dinner? Did you eat anything more?
Record first the Response of the questions of column A. collect the Response for column B/C after recording the Response of the questions of column A.</td></tr>
<tr><th></th><th></th><th>Food Category</th><th>Examples of foods</th><th>A</th><th>B</th><th>C</th></tr>
<tr><td>201</td><td>VWD_20 1 A/B/C</td><td>Starchy Staples/ Food made from grains</td><td>Rice, khichuri, Chira, muri, flattened rice, flour bread, wheat, maize, barley, oat, noodles, pasta, instant noodles</td><td>1 = Yes
2 = No</td><td>|____|____|
days</td><td>|____|____|
times</td></tr>
<tr><td>202</td><td>VWD_20 2 A/B/C</td><td>Starchy Staples/ (root, tubers, tissue)</td><td>Potatoes, sweet potatoes, kochu, wiggle, green banana, shalgam,, sagu, casava, shalook, arrowroot,</td><td>1 = Yes
2 = No</td><td>|____|____|
days</td><td>|____|____|
times</td></tr>
<tr><td>203</td><td>VWD_20 3 A/B/C</td><td>Lentils/Pulses</td><td>Dal, motor, bean seeds, peas, chickpeas, soybean, tofu, humus</td><td>1 = Yes
2 = No</td><td>|____|____|
days</td><td>|____|____|
times</td></tr>
<tr><td>204</td><td>VWD_20 4 A/B/C</td><td>Legumes and Nuts</td><td>Peanut, pistachios, kazu, almond or any kind of nuts, chia seeds, sesame seeds, flax seeds, sunflower seeds, sweet pumpkin seeds</td><td>1 = Yes
2 = No</td><td>|____|____|
days</td><td>|____|____|
times</td></tr>
<tr><td>205</td><td>VWD_20 5 A/B/C</td><td>Dark green leafy vegetables</td><td>All kinds of leafy vegetables (pui, kochu, kalmi, pumpkin, helencha, data, dekhi, jute, radish leaves)</td><td>1 = Yes
2 = No</td><td>|____|____|
days</td><td>|____|____|
times</td></tr>
<tr><td>206</td><td>VWD_20 6 A/B/C</td><td>Red/orange/yellow vegetables</td><td>Sweet pumpkin, carrot, orange or yellow fleshed sweet potato, lal shak, other</td><td>1 = Yes
2 = No</td><td>|____|____|
days</td><td>|____|____|
times</td></tr>
</table>

No	Variable	Question		Yesterday during the day or night, did you consume the following food items? (A)	During the last 07 days how many days did you consume the following food items (B)	During the last 07 days how many times did you consume the following food items (C)
			yellow/orange/red colored vegetables			
207	VWD_207 A/B/C	Red/orange/yellow fruits	Ripe mangoes, ripe papaya, *bangi/ futi*,, *deuwa* and other red/yellow/orange fruits	1 = Yes<br>2 = No	|____|____| days	|____|____| times
208	VWD_208 A/B/C	Vitamin C rich fruits	Guava, amla, lemon, Hog Plum, tomatoes orange, pomelo, grapes, pineapple, unripe mango, star fruit, plum. strawberry	1 = Yes<br>2 = No	|____|____| days	|____|____| times
209	VWD_209 A/B/C	Vitamin C rich vegetables	Unripe tomato, green chilli, cauliflower, cabbage, *kakrol*, *korolla*	1 = Yes<br>2 = No	|____|____| days	|____|____| times
210	VWD_210 A/B/C	Other vegetables or fruits	Beans, white gourd (*lau*), ladys finger, *potol*, *jhinga*, radish/mula, beet, bamboo shoots, cucumber, brinjal, lettuce, mushroom, zucchini, onion	1 = Yes<br>2 = No	|____|____| days	|____|____| times
211	VWD_211 A/B/C	Other fruits	Apple, avocado, black berry, cherry, ripe jackfruit	1 = Yes<br>2 = No	|____|____| days	|____|____| times
212	VWD_212 A/B/C	Eggs	Hen/duck, other birds,	1 = Yes<br>2 = No	|____|____| days	|____|____| times
213	VWD_213 A/B/C	Organ meat	*Gila* liver, stomach, heart, kidney	1 = Yes<br>2 = No	|____|____| days	|____|____| times
214	VWD_214 A/B/C	Meat	Beef, Pork, Veal, Lamb, Goat, Chicken, Duck or any other animal meat	1 = Yes<br>2 = No	|____|____| days	|____|____| times
215	VWD_215 A/B/C	Processed meat	Sausage, hotdog, Ham, Bacon, Salami, tinned meat	1 = Yes<br>2 = No	|____|____| days	|____|____| times
216	VWD_216 A/B/C	Small Fish	Small Fish Eaten Whole with Bones (i.e. Kachki, mola, dhela, chapila, batashi, small prawn, dried small fish)	1 = Yes<br>2 = No	|____|____| days	|____|____| times
217	VWD_217 A/B/C	Large fish/sea food	Large Whole Fish, Shellfish, Crab, octopus, squid, shark, shrimp, dried large fish, canned large fish	1 = Yes<br>2 = No	|____|____| days	|____|____| times
218	VWD_218	Dairy	Milk, cheese, yogurt or other milk products	1 = Yes<br>2 = No	|____|____| days	|____|____| times

No	Variable	Question		Yesterday during the day or night, did you consume the following food items? (A)	During the last 07 days how many days did you consume the following food items (B)	During the last 07 days how many times did you consume the following food items (C)
	A/B/C					
218	VWD_218 AA/AB/AC	Insects and other protein food	Fish egg, insects, shell etc	1 = Yes<br>2 = No	|____|____| days	|____|____| times
219	VWD_219 A/B/C	Edible oil	Ghee, butter, cream, *sor*, animal fat, margarine, mayonnaise, palm oil, vegetable oil,	1 = Yes<br>2 = No	|____|____| days	|____|____| times
220	VWD_220 A/B/C	**Savoury and fried snacks**	**Crisps and chips, fried dough or other fried snacks, *singara, samucha,* french fry, popcorn, *nimki***	1 = Yes<br>2 = No	|____|____| days	|____|____| times
221	VWD_221 A/B/C	**Sweet food**	**Sugary foods, such as chocolates, candies, cookies/sweet biscuits and cakes, sweet pastries or ice cream, any kind of sweets, honey, halwa, condensed milk, *tiler khaza***	1 = Yes<br>2 = No	|____|____| days	|____|____| times
222	VWD_222 A/B/C	Sweet drinks(except tea and coffee)	Soft drinks, juice, energy drinks, yogurt drinks, chocolate drinks, Horlicks, moltova	1 = Yes<br>2 = No	|____|____| days	|____|____| times
222	VWD_222 AA/AB/AC	Sweet drinks (Tea, coffee)	Sweetened tea or coffee	1 = Yes<br>2 = No	|____|____| days	|____|____| times
223	VWD_223 A/B/C	**Other beverages and foods**	**Unsweetened tea or coffee, Soup, clear broth, alcohol, pickles, any other food not listed above**	1 = Yes<br>2 = No	|____|____| days	|____|____| times
224	VWD_224 A/B/C	Condiments/spices	Spices, coriander leaves, sauce, garlic, ketchup, lemon juice, mint leaves, betel leaves, tobacco leaves, *jarda*	1 = Yes<br>2 = No	|____|____| days	|____|____| times

Section 6: Access to nutrition sensitive information and services
Income generating activities received from CARE, UNICEF or other elsewhere

The Project is funded by the European Union

Sl #		Questions	Response	Code	Instructions
603	VNSN_ 603	If yes, what kind of help/support or training you or your household member received?	1= Support for chicken rearing in the home backyard or training<br><br>2= Tailoring training<br><br>99= Other IGA assistance or training<br><br>____________		
