# Supplementary material for: Integrated nutrition-sensitive interventions are associated with improvements in household food security and dietary diversity among vulnerable women in the host communities of Cox’s Bazar, Bangladesh: Findings from a pre-post comparison study
Source: PLoS One. 2026 Nov 5;21(11):e0362124. doi: 10.1371/journal.pone.0362124 (PMC13739155; doi:10.1371/journal.pone.0362124)
Supplement: Supplementary file 2 [file pone.0362124.s004.pdf]

The Project is funded by the European Union

মডিউল 1.1: খানাপ্রধানের মডিউল

পরিচ্ছেদ 1: সাধারণ তথ্যাবলী এবং খানার আর্থ-সামাজিক অবস্থা

নম্বর	সূচক	প্রশ্ন	উত্তর		কোড	নির্দেশনা
102	HS_102	উপজেলার নাম ও কোড	____________ |___|___|			
110	SE_110	আপনার খানার রান্নার কাজে প্রধানত কি ধরনের জ্বালানী ব্যবহার করেন ?	1 = বিদ্যুৎ<br>2 = এলপিজি<br>3 = পাইপযুক্ত প্রাকৃতিক গ্যাস<br>4 = কেরোসিন<br>5 = কয়লা/লিগনাইট<br>6 = চারকোল<br>7 = কাঠ<br>8 = পাটখড়ি/খড়/নাড়া/পাতা<br>9 = গোবর<br>10 = বায়োগ্যাস<br>99 = অন্যান্য (নির্দিষ্ট করুন)			'1-4' হলে 111 এ যান।
111	আপনার খানায় বর্তমানে গৃহপালিত পশু বা পাখি আছে? যদি থাকে, তবে কতগুলো?					
			A.	B.		
			1= হ্যাঁ 2 = না	সংখ্যা (যদি হ্যাঁ হয়)		
	SE_111A	গরু/মহিষ	1= হ্যাঁ 2 = না	|___|___|		যদি সংখ্যাটি 95 এর বেশি হয় তাহলে 95 কোড করুন
	SE_111B	ভেড়া/ছাগল/শুকর	1= হ্যাঁ 2 = না	|___|___|		
	SE_111C	মুরগী/হাঁস/রাজহাঁস	1= হ্যাঁ 2 = না	|___|___|		
	SE_111D	ছোট পশু/পাখি (যেমন: খরগোশ, কবুতর ইত্যাদি)	1= হ্যাঁ 2 = না	|___|___|		
	SE_111E	অন্যান্য পশু পাখি আছে কি?				
	SE_111ET	অন্যান্য (নির্দিষ্ট করুন) ———	1= হ্যাঁ 2 = না	|___|___|		
112	আপনার খানায় নিম্নে বর্ণিত কোন কোন জিনিসগুলো সচল অবস্থায় আছে ?					
	SE_112A	বিদ্যুৎ সংযোগ	1= হ্যাঁ 2 = না			
	SE_112B	সৌর বিদ্যুৎ	1= হ্যাঁ 2 = না			
	SE_11C	রেডিও	1= হ্যাঁ 2 = না			
	SE_112CA	টেলিভিশন	1= হ্যাঁ 2 = না			
	SE_112D	মোবাইল ফোন	1= হ্যাঁ 2 = না			
	SE_112DA	টেলিফোন	1= হ্যাঁ 2 = না			
	SE_112D2	স্মার্ট ফোন	1= হ্যাঁ 2 = না			
	SE_112DB	কম্পিউটার/ল্যাপটপ	1= হ্যাঁ 2 = না			
	SE_112DC	ডিভিডি/ভিসিডি প্লেয়ার	1= হ্যাঁ 2 = না			
	SE_112E	ফ্যান	1= হ্যাঁ 2 = না			
	SE_112F	খাট/চৌকি	1= হ্যাঁ 2 = না			
	SE_112G	আলমীরা/ওয়াড্রব/শোকেস	1= হ্যাঁ 2 = না			
	SE_112H	রেফ্রিজারেটর/ফ্রিজ	1= হ্যাঁ 2 = না			
	SE_112HA	এয়ার কণ্ডিশনার	1= হ্যাঁ 2 = না			
	SE_112I	টেবিল/চেয়ার	1= হ্যাঁ 2 = না			

নম্বর	সূচক	প্রশ্ন	উত্তর	কোড	নির্দেশনা
	SE_112J	ঘড়ি/দেয়াল ঘড়ি	1= হ্যাঁ 2 = না		
	SE_112K	বাই-সাইকেল	1= হ্যাঁ 2 = না		
	SE_112L	মোটরসাইকেল/স্কুটার	1= হ্যাঁ 2 = না		
	SE_112LA	অটোবাইক/টেম্পু/সিএনজি	1= হ্যাঁ 2 = না		
	SE_112M	পশুচালিত গাড়ি	1= হ্যাঁ 2 = না		
	SE_112N	কার/ট্রাক	1= হ্যাঁ 2 = না		
	SE_112O	নৌকা	1= হ্যাঁ 2 = না		
	SE_112P	ইঞ্জিনচালিত নৌকা	1= হ্যাঁ 2 = না		
	SE_112Q	রিক্সা/ভ্যান	1= হ্যাঁ 2 = না		
	SE_112R	পাওয়ার টিলার	1= হ্যাঁ 2 = না		
	SE_112S	শ্যালো মেশিন/পানির পাম্প	1= হ্যাঁ 2 = না		
	SE_112T	লাঙ্গল	1= হ্যাঁ 2 = না		
	SE_112U	আইপিএস/বৈদ্যুতিক জেনারেটর	1= হ্যাঁ 2 = না		
	SE_112V	মাছ ধরার জাল	1= হ্যাঁ 2 = না		
	SE_112W	সেলাই মেশিন	1= হ্যাঁ 2 = না		
	SE_112X	মাইক্রোওভেন/ইলেকট্রিক ওভেন	1= হ্যাঁ 2 = না		
113	SE_113	আপনার প্রধান বসত ঘরের দেয়াল প্রধানত কি দিয়ে তৈরী?<br><br>(পর্যবেক্ষণ করে লিপিবদ্ধ করুন)	**স্বাভাবিক দেয়াল :**<br>1 = দেয়াল নেই<br>2 = বেত, ইক্ষু জাতীয় পাতা/তালপাতা/গাছের গুড়ি/বাঁশ<br>3 = কাদা মাটি<br>**প্রাথমিক পর্যায়ের দেয়াল:**<br>4 = বাঁশ ও মাটি<br>5 = পাথর ও মাটি<br>6 = প্লাইউড<br>7 = পিচ বোর্ড/কার্ড বোর্ড/পলিথিন<br>**পরিপূর্ণ দেয়াল:**<br>8 = টিন<br>9 = ইট-সিমেন্ট<br>10 = পাথর ও চুন/ সিমেন্ট<br>11 = কাঠের তক্তা/ ফলক<br>12 = ইট<br>13 = সিমেন্টের ব্লক<br>99 = অন্যান্য (নির্দিষ্ট করুন)		পর্যবেক্ষণ করে লিপিবদ্ধ করুন

নম্বর	সূচক	প্রশ্ন	উত্তর	কোড	নির্দেশনা
114	SE_114	আপনার প্রধান বসত ঘরের চাল বা ছাদ প্রধানত কি দিয়ে তৈরী?<br><br>(পর্যবেক্ষণ করে লিপিবদ্ধ করুন)	**স্বাভাবিক ছাদ :**<br>1= ছাদ নেই<br>2= খড়/তালপাতা/ গোল পাতা প্রভৃতি<br>**কাঁচাছাদ :**<br>3= বাঁশ ও মাটি<br>4= কাঠের তক্তা<br>5= পিচ বোর্ড/ পলিথিন/কার্ড বোর্ড<br>**পরিপূর্ণছাদ :**<br>6= টিন<br>7= কাঠ<br>8= সিরামিক টাইলস/টালি<br>9= ইট-সিমেন্ট<br>10= কাঠের ফলক<br>99= অন্যান্য (নির্দিষ্ট করুন)		পর্যবেক্ষণ করে লিপিবদ্ধ করুন
115	SE_115	আপনার প্রধান বসত ঘরের মেঝে প্রধানত কি দিয়ে তৈরী?<br><br>(পর্যবেক্ষণ করে লিপিবদ্ধ করুন)	**কাঁচামেঝে :**<br>1= মাটি/বালি<br>**প্রাথমিক পর্যায়েরমেঝে :**<br>2= কাঠের তক্তা<br>3= তাল গাছ/বাঁশ<br>**পরিপূর্ণমেঝে :**<br>4= সিরামিক টাইলস<br>5= ইট-সিমেন্ট<br>6= মোজাইক<br>99= অন্যান্য (নির্দিষ্ট করুন)..............		পর্যবেক্ষণ করে লিপিবদ্ধ করুন
116	SE_116	আপনার খানায় বসবাসের জন্য কয়টি কক্ষ আছে?(গৃহকর্মীদের কক্ষসহ)	|_____|_____|		
122	SE_122	আপনার পরিবারের গড় মাসিক আয় কত?	|__|__|__|__|__|__|__| টাকা<br>88 = জানাতে অসম্মতি		

পরিচ্ছেদ 2: খানার পানি সরবরাহ, পয়ঃনিস্কাশন ও পরিষ্কার-পরিচ্ছন্নতা

নম্বর	সূচক	প্রশ্ন	উত্তর	কোড	নির্দেশনা
201	WH_201	আপনারা প্রধানত কিসের পানি পান করেন?	**পাইপের পানি**<br>1 = ঘরের ভিতর পাইপ<br>2 = বাড়ীর উঠানে পাইপ<br>3 = পাবলিক ট্যাপ<br>**টিউবওয়েল অথবা গর্তপাইপ**<br>4 = যৌথ<br>5= একক<br>**কুয়ার পানি**<br>6 = ঢাকনাযুক্ত/সুরক্ষিত<br>7 = ঢাকনা ছাড়া/সুরক্ষিত নয়<br>8 = বৃষ্টির পানি		

The Project is funded by the European Union

নম্বর	সূচক	প্রশ্ন	উত্তর	কোড	নির্দেশনা
			**ভূপৃষ্ঠ পানি**<br>9 = পুকুর/নদী/খাল/হাওর/সেচ নালা<br>10 = পানির ট্যাংক<br>**ঝর্ণার পানি**<br>11 = সুরক্ষিত<br>12 = সুরক্ষিত নয়<br>13 = পানির ট্যাংকের ট্রাক<br>14 = পানির ট্যাংকের ভ্যান/ছোট গাড়ি<br>15 = বোতলজাত পানি<br>99 = অন্যান্য (নির্দিষ্ট করুন).........		
205	WH_205	আপনারা কি ধরনের পায়খানা ব্যবহার করেন?<br><br>(পর্যবেক্ষণ করে লিপিবদ্ধ করুন)	1 = স্যুয়ারেজ লাইনের সাথে যুক্ত<br>2 = স্যাপ্টিক ট্যাংকসহ পায়খানা<br>3 = ওয়াটার সিল সহ রিংস্লাব<br>4 = ওয়াটার সিল ছাড়া রিংস্লাব<br>5 = আবদ্ধ পিট<br>6 = খোলাপিট<br>7 = ঝুলন্ত পায়খানা<br>8 = পায়খানা নাই (রাস্তা/নদীর ধারে/খোলা মাঠে/যেখানে সেখানে/ঝোপ-জঙ্গল)<br>99 = অন্যান্য (নির্দিষ্ট করুন)............................		‘8’ হলে 207 এ যান।
206	WH_206	আপনারা কি নিজস্ব বা যৌথ বা পাবলিক টয়লেট ব্যবহার করেন?<br>(পায়খানাটি যেখানেই অবস্থিত হোক না কেন, প্রশ্নটি করতে হবে।<br>যৌথ টয়লেট: নির্দিষ্ট সংখ্যক ব্যবহারকারীর মালিকানাধীন টয়লেট।<br>পাবলিক টয়লেট: ব্যবহারকারীর সংখ্যা নির্দিষ্ট নয় এমন টয়লেট।)	1 = নিজের<br>2 = যৌথভাবে<br>3 = পাবলিক টয়লেট<br>77 = জানিনা		

পরিচ্ছেদ 3A খাদ্যনিরাপত্তা এবং মানিয়ে নেয়ার কৌশল

এখন, আমি আপনাকে আপনার খানার খাদ্য নিরাপত্তা বিষয়ক কিছু প্রশ্ন জিজ্ঞাসা করার জন্য আপনার আরও কিছু সময় নিব। আমি আপনাকে পূর্বের 4 সপ্তাহের কথা চিন্তা করে বলতে বলব যে, কতটা ঘনঘন এবং কিভাবে আপনার খানায় খাদ্যের নিরাপত্তাহীনতার অভিজ্ঞতা হয়েছে। মনে রাখবেন যে, আমি খানা বলতে বুঝাতে চাইছি ঐ সকল সদস্য যারা সাধারণত একই হাড়ি থেকে খাবার ভাগাভাগি করে খান। যদি আপনার কোন প্রশ্ন থাকে তাহলে আপনি আমায় থামিয়ে জিজ্ঞাসা করবেন।

নম্বর	সচক	প্রশ্ন	উত্তর	কোড	নির্দেশনা
301	FS_301	গত 4 সপ্তাহে আপনি আপনার খানায় পর্যাপ্ত খাদ্য নাই অথবা কিভাবে যোগাড় করবেন, এই দুশ্চিন্তায় ছিলেন কি?	0 = কখনো না<br>1 = হ্যাঁ হলে কতবার কদাচিৎ (একবার অথবা দুইবার)<br>2 = মাঝেমধ্যে (3 থেকে 10বার)<br>3 = প্রায়ই (10 বার এর অধিক)		
302	FS_302	গত 4 সপ্তাহে এমন হয়েছিলে কি, আপনি বা আপনার খানার কোন সদস্য খাবার অথবা টাকার অভাবের কারণে পছন্দের খাবার খেতে পারেনি?	0 = কখনো না<br>1 = হ্যাঁ হলে কতবার কদাচিৎ (একবার অথবা দুইবার)<br>2 = মাঝেমধ্যে (3 থেকে 10বার)<br>3 = প্রায়ই (10 বার এর অধিক)		

নম্বর	সচক	প্রশ্ন	উত্তর	কোড	নির্দেশনা
303	FS_303	গত 4 সপ্তাহে আপনি বা খানার কোনো সদস্যকে অভাবের কারণে শুধুমাত্র ভাত খেয়ে থাকতে হয়েছিল কি (লবণ, মরিচ ও পিঁয়াজ ইত্যাদি দিয়ে)?	0 = কখনো না<br>1 = হ্যাঁ হলে কতবার কদাচিৎ (একবার অথবা দুইবার)<br>2 = মাঝেমধ্যে (3 থেকে 10বার)<br>3 = প্রায়ই (10 বার এর অধিক)		
304	FS_304	গত 4 সপ্তাহে আপনি বা খানার কোনো সদস্যকে অভাবের কারণে এমন কোন খাবার খেতে হয়েছিল যা সাধারনত খান না (যেমনঃ বন্য বা অনাবাদী খাবার বা ভাতের পরিবর্তে অন্য কোন খাবার, যা সাধারনত খান না)?	0 = কখনো না<br>1 = হ্যাঁ হলে কতবার কদাচিৎ (একবার অথবা দুইবার)<br>2 = মাঝেমধ্যে (3 থেকে 10বার)<br>3 = প্রায়ই (10 বার এর অধিক)		
305	FS_305	খানায় পর্যাপ্ত খাবার অথবা খাবার কেনার জন্য টাকা না থাকার কারণে গত 4 সপ্তাহে আপনি বা আপনার খানার কোনো সদস্যকে কি কোন বেলা (যেমন: সকাল, দুপুর অথবা রাতের খাবার) প্রয়োজনের তুলনায় কম খেতে হয়েছিল?	0 = কখনো না<br>1 = হ্যাঁ হলে কতবার কদাচিৎ (একবার অথবা দুইবার)<br>2 = মাঝেমধ্যে (3 থেকে 10 বার)<br>3 = প্রায়ই (10 বার এর অধিক)		
306	FS_306	গত 4 সপ্তাহে আপনি বা আপনার খানার কোনো সদস্যকে খাবারের অভাব অথবা খাবার কেনার টাকা না থাকার কারণে কি কোন বেলা না খেয়ে থাকতে হয়েছিল?	0 = কখনো না<br>1 = হ্যাঁ হলে কতবার কদাচিৎ (একবার অথবা দুইবার)<br>2 = মাঝেমধ্যে (3 থেকে 10 বার)<br>3 = প্রায়ই (10 বার এর অধিক)		
307	FS_307	গত 4 সপ্তাহে কখনও কি আপনার ঘরের খাবার শেষ হয়ে গিয়েছিল এবং ঐ দিন খাবার কেনার জন্য কোনো টাকাও ছিল না?	0 = কখনো না<br>1 = হ্যাঁ হলে কতবার কদাচিৎ (একবার অথবা দুইবার)<br>2 = মাঝেমধ্যে (3 থেকে 10 বার)<br>3 = প্রায়ই (10 বার এর অধিক)		
308	FS_308	গত 4 সপ্তাহে কি কখনও এমন হয়েছিল যে, আপনাকে অথবা খানার কোনো সদস্যকে অভাবের কারণে ক্ষুধার্ত বা অভুক্ত অবস্থায় রাতে ঘুমাতে যেতে হয়েছিল?	0 = কখনো না<br>1 = হ্যাঁ হলে কতবার কদাচিৎ (একবার অথবা দুইবার)<br>2 = মাঝেমধ্যে (3 থেকে 10 বার)<br>3 = প্রায়ই (10 বার এর অধিক)		
309	FS_309	গত 4 সপ্তাহে কখনও এমন হয়েছিল যে আপনি বা খানার কোনো সদস্যকে খাবারের অভাবের কারণে না খেয়ে সারা দিন-রাত কাটাতে হয়েছিল?	0 = কখনো না<br>1 = হ্যাঁ হলে কতবার কদাচিৎ (একবার অথবা দুইবার)<br>2 = মাঝেমধ্যে (3 থেকে 10 বার)<br>3 = প্রায়ই (10 বার এর অধিক)		'0' হলে 310 এ যান।

মডিউল 1.6: সুবিধা বঞ্চিত মহিলার মডিউল

পরিচ্ছেদ 1: সাধারণ তথ্যাবলী

নম্বর	সূচক	প্রশ্ন	উত্তর	কোড	নির্দেশনা
102	VGI _102A	সুবিধা বঞ্চিত মহিলার বয়স	A.|____|___| বছর		
103	VGI_103	আপনার বৈবাহিক অবস্থা কি?	1 = অবিবাহিত<br>2 = বর্তমানে বিবাহিত<br>3 =পৃথক<br>4 = তালাকপ্রাপ্ত<br>5 = বিপত্নিক/বিধবা<br>88 = জানাতে অসম্মতি		
104	VGI_104	আপনি কত বছরের প্রাতিষ্ঠানিক শিক্ষা শেষ করেছেন?<br><br>(সম্পূর্ণ বছর লিখুন। প্রথম শ্রেণীর নিচে এবং উপানুষ্ঠানিক শিক্ষা অন্তর্ভুক্ত হবে না) *[নির্দেশনাঃ তথ্য প্রদানকারী সর্বমোট কত বছর প্রাতিষ্ঠানিক শিক্ষা সম্পন্ন করেছেন তা লিপিবদ্ধ করুন। প্রাতিষ্ঠানিক শিক্ষা না থাকলে 00 লিখুন।]*	|__|__| বছর<br>5 = প্রাথমিক শিক্ষা/এবতেদায়ী<br>10 = মাধ্যমিক/দাখিল<br>12 = উচ্চ মাধ্যমিক/ ডিপ্লোমা/ আলিম<br>16 = স্নাতক/ফাজিল<br>18 = স্নাতকোত্তর/ কামিল/ দাওরা		
105	VGI_105	গত 12 মাসে আপনার পেশা কি ছিল?<br><br>*[নির্দেশনাঃ যদি তথ্য প্রদানকারী গত 12 মাসে একাধিক পেশায় নিযুক্ত থাকেন তাহলে তিনি যে পেশাটিতে বেশী সময় ব্যয় করেছেন সেটিকে প্রধান পেশা হিসাবে বিবেচনা করুন এবং তা লিপিবদ্ধ করুন।]*	1 = কৃষিকাজ (ধান)<br>2= কৃষিকাজ (অন্যান্য)<br>3 = কৃষি দিনমজুর<br>4 = অদক্ষ দিনমজুর<br>5 = দক্ষ দিনমজুর<br>6 = রিক্সা/ভ্যান/ঠেলাগাড়ী/বেবীট্যাক্সি ড্রাইভার/নৌকার মাঝি<br>7 = জেলে<br>8 = চাকুরীজীবি<br>9 = পেশাজীবি<br>10 = ব্যবসায়ী<br>11 = ক্ষুদে ব্যবসায়ী<br>12 = গৃহপরিচারিকা<br>13 = কৃষিকাজ-জুমচাষি<br>14 = উপার্জন করে না<br>15 = হাঁস/মুরগি পালন/পশু পালন<br>16 = হস্তশিল্প<br>17 = কৃষিকাজ-শাক-সব্জি চাষি<br>18 = মৎস চাষ<br>19 = গৃহিনী<br>20 = ছাত্র/ছাত্রী<br>21 =প্রবাসী<br>22 = সেলাই কাজ<br>77 = জানিনা		

নম্বর	সূচক	প্রশ্ন	উত্তর	কোড	নির্দেশনা
			99 = অন্যান্য (নির্দিষ্ট করুন)		

পরিচ্ছেদ 2: সুবিধাভোগী সুবিধা বঞ্চিত মহিলার খাদ্য গ্রহণের বৈচিত্র্য

নম্বর	সূচক	প্রশ্ন		গতকাল দিনে বা রাতে আপনি নিম্নবর্ণিত খাবারগুলো খেয়েছেন কি? (A)	গত 7 দিনে আপনি নিম্নবর্ণিত খাবারগুলো কতদিন খেয়েছেন? (B)	গত 7 দিনে আপনি নিম্নবর্ণিত খাবারগুলো কতবার খেয়েছেন? (C)
এখন আমি আপনাকে গতকাল দিনে বা রাতে (গতকাল সকাল 6.00 টা থেকে আজ সকাল 6.00 টা পর্যন্ত) আপনি বাড়িতে ও বাড়ির বাইরে যা যা খেয়েছেন তা বর্ণনা করতে অনুরোধ করছি। দয়া করে সব ধরণের খাবার, পানীয় যা আপনি সকাল, দুপুর ও রাতের খাবারের সাথে খেয়েছেন বা নাস্তা বা হালকা নাস্তা করেছেন সে সম্পর্কে বলুন। আপনি খাবার তৈরীর সময় কোন খাবার খেয়ে থাকলে তাও মনে করে আমাদেরকে বলুন। আপনি গতকাল সকালে যা খেয়েছেন তা দিয়েই শুরু করুন।<br>-আপনি সকালে ঘুম থকে উঠে কি কি খেয়েছেন? আরো কিছু খেয়েছেন কি?<br>-সকালে আরো কি কি খেয়েছেন? আরো কিছু খেয়েছেন কি?<br>-দুপুরে আরো কি কি খেয়েছেন? আরো কিছু খেয়েছেন কি?<br>-বিকালে আরো কি কি খেয়েছেন? আরো কিছু খেয়েছেন কি?<br>-সন্ধ্যায় আরো কি কি খেয়েছেন? আরো কিছু খেয়েছেন কি?<br>-রাতে আরো কি কি খেয়েছেন? আরো কিছু খেয়েছেন কি?<br>**A কলামের প্রশ্নগুলোর উত্তর প্রথমে রেকর্ড করুন। A কলামের প্রশ্নগুলোর উত্তর রেকর্ড করা শেষ হলে B/C কলামের উত্তর নিন।**						
		খাবার ধরণ	খাবার নমুনা	A	B	C
201	VWD_201 A/B/C	শ্বেতসার জাতীয় (শস্য জাতীয় খাবার)	ভাত, খিচুড়ি, চিড়া, মুড়ি, আটার রুটি, গম, ভুট্টা, বার্লি, ওটস, নুডলস, পাস্তা, ইনষ্ট্যান্ট নুডলস	1 = হ্যাঁ 2 = না	|___|___| দিন	|___|___| বার
202	VWD_202 A/B/C	শ্বেতসার জাতীয় (মূল, কন্দ, কলা)	গোলআলু, মিষ্টিআলু, কচু, কচুমুখী, কাঁচকলা, শালগম, সাগু, কাসাভা, শালুক, এরারুট	1 = হ্যাঁ 2 = না	|___|___| দিন	|___|___| বার
203	VWD_203 A/B/C	ডাল ও ডালজাতীয় খাবার	ডাল, মটর, শিমের বীচি, বুট, ছোলা, সয়াবীন, টফু, হুমাস	1 = হ্যাঁ 2 = না	|___|___| দিন	|___|___| বার
204	VWD_204 A/B/C	বাদাম ও তৈলবীজ	চীনাবাদাম, পেস্তা, কাজু, কাঠবাদাম অথবা যেকোনো বাদাম, চিয়াসীড, তিল, তিসি, সূর্যমুখীবীজ, মিষ্টিকুমড়া বীজ	1 = হ্যাঁ 2 = না	|___|___| দিন	|___|___| বার
205	VWD_205 A/B/C	গাঢ় সবুজপাতা জাতীয় শাক	সকল ধরণের পাতা জাতীয় শাক (পুঁই, কচু, কলমি, কুমড়া শাক, হেলেঞ্চা শাক, ডাটা শাক, ঢেঁকি শাক, পাট শাক, মূলা শাক)	1 = হ্যাঁ 2 = না	|___|___| দিন	|___|___| বার
206	VWD_206 A/B/C	লাল/কমলা/হলুদ সবজি	মিষ্টিকুমড়া, গাজর, গাঢ় হলুদ বা কমলা মিষ্টি আলু, লাল শাক ও অন্যান্য লাল/কমলা/হলুদ রঙের সবজি	1 = হ্যাঁ 2 = না	|___|___| দিন	|___|___| বার
207	VWD_207 A/B/C	লাল/কমলা/হলুদ ফলমূল	পাকা আম, পাকা পেঁপে, ফুটি ও ডেউয়া অন্যান্য লাল/কমলা/হলুদ রঙের ফল	1 = হ্যাঁ 2 = না	|___|___| দিন	|___|___| বার

The Project is funded by the European Union

unicef

BRAC JAMES P GRANT SCHOOL OF PUBLIC HEALTH

নম্বর	সূচক	প্রশ্ন		গতকাল দিনে বা রাতে আপনি নিম্নবর্ণিত খাবারগুলো খেয়েছেন কি? (A)	গত 7 দিনে আপনি নিম্নবর্ণিত খাবারগুলো কতদিন খেয়েছেন? (B)	গত 7 দিনে আপনি নিম্নবর্ণিত খাবারগুলো কতবার খেয়েছেন? (C)
208	VWD_208 A/B/C	ভিটামিন সি-সমৃদ্ধ ফল	পেয়ারা, আমলকি, লেবু, কমলালেবু, মালটা, জাম্বুরা, আমড়া, আনারস, কাঁচা আম, টমেটো, কামরাঙা, বরই, জাম	1 = হ্যাঁ 2 = না	|____|____| দিন	|____|____| বার
209	VWD_209 A/B/C	ভিটামিন সি-সমৃদ্ধ সবজি	কাঁচা টমেটো, কাঁচা মরিচ, ফুলকপি, বাঁধাকপি, কাঁকরোল, করল্লা	1 = হ্যাঁ 2 = না	|____|____| দিন	|____|____| বার
210	VWD_210 A/B/C	অন্যান্য শাকসবজি	শিম, লাউ, ঢেড়স, পটল, ঝিঙ্গা, বরবটি, বিট, কচি বাঁশ, শসা, বেগুন, লেটুস, মাশরুম, মূলা, জুকিনি, পিঁয়াজ, সেলারি	1 = হ্যাঁ 2 = না	|____|____| দিন	|____|____| বার
211	VWD_211 A/B/C	অন্যান্য ফলমূল	আপেল, চেরী, পাকা কাঁঠাল, তরমুজ, বাঙ্গি, পাকা কলা, আঙ্গুর, নাশপাতি, খেজুর, ডুমুর, নারকেলের শাঁস	1 = হ্যাঁ 2 = না	|____|____| দিন	|____|____| বার
212	VWD_212 A/B/C	ডিম	হাঁস-মুরগি, অন্যান্য পাখির ডিম	1 = হ্যাঁ 2 = না	|____|____| দিন	|____|____| বার
213	VWD_213 A/B/C	অঙ্গ জাতীয় মাংস	গিলা, কলিজা, পাকস্থলী, হৃদপিন্ড, কিডনি	1 = হ্যাঁ 2 = না	|____|____| দিন	|____|____| বার
214	VWD_214 A/B/C	মাংস	হাঁস, মুরগি, গরু, ছাগল, ভেড়া, মহিষ, শুকর, বা যেকোনো প্রাণীর মাংস	1 = হ্যাঁ 2 = না	|____|____| দিন	|____|____| বার
215	VWD_215 A/B/C	প্রক্রিয়াজাত মাংস	সসেজ, হট ডগ, হ্যাম, বেকন, সালামি, কৌটাজাত মাংস	1 = হ্যাঁ 2 = না	|____|____| দিন	|____|____| বার
216	VWD_216 A/B/C	ছোট মাছ	ছোট মাছের কাঁটা বা হাড়সহ (কাচঁকি, মলা, ঢেলা, চাপিলা, বাতাসি, গুঁড়া চিংড়ি, ছোট মাছের শুঁটকি)	1 = হ্যাঁ 2 = না	|____|____| দিন	|____|____| বার
217	VWD_217 A/B/C	বড় মাছ/সামুদ্রিক মাছ/ সামুদ্রিক খাবার	বড় মাছ, ঝিনুক, কাঁকড়া, অক্টোপাস, স্কুইড, হাঙর, বড় চিংড়ি, নোনা ইলিশ, বড় মাছের শুঁটকি, টিনজাত বড় মাছ	1 = হ্যাঁ 2 = না	|____|____| দিন	|____|____| বার
218	VWD_218	দুধ ও দুগ্ধ জাতীয় খাবার	দুধ, পনির, দই, পায়েস অথবা অন্যান্য দুগ্ধজাতীয় খাদ্য	1 = হ্যাঁ 2 = না	|____|____| দিন	|____|____| বার
	VWD_218 AA/AB/AC	পোকা ও অন্যান্য আমিষ জাতীয় খাবার	মাছের ডিম, পোকা, শামুক ইত্যাদি	1 = হ্যাঁ 2 = না	|____|____| দিন	|____|____| বার
219	VWD_219 A/B/C	তৈল ও চর্বি জাতীয় খাবার	ঘি, মাখন, ক্রিম, সর, চর্বি, মার্জারিন, ম্যায়োনেজ, পাম অয়েল, উদ্ভিজ্জ তেল	1 = হ্যাঁ 2 = না	|____|____| দিন	|____|____| বার
220	VWD_ 220 A/B/C	স্যাভরি এবং ভাজা খাবার/স্ন্যাক্স	খাস্তা, চিপস ও অন্যান্য ভাজা খাবার, সিংগারা, সমোচা, ফ্রেঞ্চ ফ্রাই, পপ কর্ন, চানাচুর, নিমকি	1 = হ্যাঁ 2 = না	|____|____| দিন	|____|____| বার

নম্বর	সূচক	প্রশ্ন		গতকাল দিনে বা রাতে আপনি নিম্নবর্ণিত খাবারগুলো খেয়েছেন কি? (A)	গত 7 দিনে আপনি নিম্নবর্ণিত খাবারগুলো কতদিন খেয়েছেন? (B)	গত 7 দিনে আপনি নিম্নবর্ণিত খাবারগুলো কতবার খেয়েছেন? (C)
221	VWD_221 A/B/C	মিষ্টি জাতীয় খাবার	চিনিযুক্ত খাবার যেমন চকলেটস, ক্যান্ডিস, কুকিজ/মিষ্টি বিস্কিট এবং কেক, মিষ্টি পেস্ট্রি বা আইসক্রিম, যেকোনো মিষ্টি যেমন রসগোল্লা, চমচম, লালমোহন, কালোজাম ইত্যাদি, মধু, হালুয়া, কনডেন্সড দুধ, তিলের খাজা	1 = হ্যাঁ 2 = না	|____|____| দিন	|____|____| বার
222	VWD_222 A/B/C	মিষ্টি পানীয় (চা/কফি ব্যতীত)	সফট ড্রিংক্স, সরবত, চিনিযুক্ত ফলের জুস, ফলের সুগন্ধযুক্ত পানীয়, এনার্জি ড্রিংক্স, ইয়োগার্ট ড্রিংক্স, চকোলেট ড্রিংক্স, হরলিক্স, মল্টোভা	1 = হ্যাঁ 2 = না	|____|____| দিন	|____|____| বার
222	VWD_222 AA/AB/AC	মিষ্টি পানীয় (চা/কফি)	চিনিযুক্ত চা বা কফি	1 = হ্যাঁ 2 = না	|____|____| দিন	|____|____| বার
223	VWD_223 A/B/C	অন্যান্য পানীয় এবং খাবার	চিনি ছাড়া চা অথবা কফি, স্যুপ, ক্লিয়ার ব্রথ, অ্যালকোহল, আচার, উপরের তালিকাভুক্ত নয় এমন যেকোন পানীয় এবং খাবার	1 = হ্যাঁ 2 = না	|____|____| দিন	|____|____| বার
224	VWD_224 A/B/C	খাবার সুস্বাদু করার দ্রব্যাদি/ মশলা	মশলা, সস, রসুন, কেচাপ, লেবুর রস, পুদিনা পাতা, ধনেপাতা, পান, সুপারি, তামাক পাতা, জর্দা, কালোজিরা, সরিষা, মিষ্টি জিরা, জিরা, আদা, হলুদ	1 = হ্যাঁ 2 = না	|____|____| দিন	|____|____| বার

পরিচ্ছেদ 6: নিউট্রিশন সেনসিটিভ তথ্য ও সেবার প্রাপ্যতা সম্পর্কিত তথ্য

কেয়ার (CARE)-ইউনিসেফ **বা অন্য কোথাও থেকে প্রাপ্ত আয় বৃদ্ধিমূলক সহায়তা বা কর্মকাণ্ড (IGA)**

নম্বর	সূচক	প্রশ্ন	উত্তর	কোড	নির্দেশনা
603	VNSN_603	যদি হ্যাঁ হয়, আপনি অথবা আপনার খানার সদস্য কোন ধরণের সহায়তা বা প্রশিক্ষণ পেয়েছেন?	1= বাড়ির আঙ্গিনায় মুরগি লালনপালনের জন্য সহায়তা বা প্রশিক্ষণ<br>2= সেলাই কাজের জন্য সহায়তা বা প্রশিক্ষণ<br>99= অন্যান্য IGA সহায়তা বা প্রশিক্ষণ<br>______________		
