# Supplementary material for: Integrated nutrition-sensitive interventions are associated with improvements in household food security and dietary diversity among vulnerable women in the host communities of Cox’s Bazar, Bangladesh: Findings from a pre-post comparison study
Source: PLoS One. 2026 Nov 5;21(11):e0362124. doi: 10.1371/journal.pone.0362124 (PMC13739155; doi:10.1371/journal.pone.0362124)
Supplement: Supplementary file 3 [file pone.0362124.s001.docx]

**Supplementary Table S1:** Summary of the intervention

| Key component | Description | Implementation Strategy | Expected Outcome |
| --- | --- | --- | --- |
| Target Population | - 2,000 vulnerable women-headed households in host communities | - Selection through participatory rural appraisal (PRA) method | - Improved food security, dietary diversity, and financial stability |
| Training on Income-Generating Activities (IGA) | - Backyard poultry rearing training (1600 beneficiaries) - Tailoring training (400 beneficiaries) | - Training of the trainers (who provided training at the beneficiary level) on FAO-supported backyard poultry training module - Tailoring training by expert trainers from ACLAB | - Skill development of trainers and beneficiaries |
| Support for tailoring beneficiaries | - One-month training - Tailoring (sewing) machines and other starter kits | - Business setup assistance and ongoing support | - Small-scale tailoring businesses established - Income generation |
| Support for backyard poultry-rearing beneficiaries | - Training on backyard poultry rearing - Distribution of *Sonali* chicken/poultry and supplies - Poultry vaccination campaign - Egg-hatching support from external vendors | - Involvement of local livestock offices for technical support - Business setup assistance and ongoing support | - Improved household food security and nutritional status - Income generation |
| Regular group session, nutrition education, and follow-up visit | - Field Facilitators (FF) of IGA projects were responsible for providing nutrition education or counseling to beneficiaries about nutrition, such as which foods contain nutrition, the importance of daily diversified food consumption, the importance of IFA intake for adolescents, complementary feeding practices for children, and hygiene practices. - Progress on IGA activities (seasonal diseases, feed management, hatching eggs, establishing small businesses, and how to attract tailoring customers). - Promotion of nutritional behaviors (importance of nutritious diet, Ante-natal Care (ANC), Post-natal Care (PNC), growth monitoring and promotion (GMP) and Infant and Young Child Feeding (IYCF) practices, etc.) - Promotion of water sanitation and hygiene (WASH) practices | - Mandatory monthly group meeting - Door-to-door follow-up visits | - Improved knowledge and practices of IGA activities, nutrition education, and WASH |
| Health facility linkages | - Provide information on how to access health services at community clinics and *upazila* health complexes - Send beneficiaries and their family members to access nutrition services such as GMP, ANC, PNC, IYCF counseling, IFA tablets, etc. | - Monthly group meeting - Liaising with health sector personnel | - Improved health and nutrition service intake |
| Cash assistance | - BDT 30,000 (USD 244.17) per beneficiary in six-month installments | - Disbursed via mobile bank account payment system | - Increased purchasing power and financial resilience |
| Market linkages | - Support in selling poultry and tailoring products | - Field teams facilitate linkage to local markets | - Enhanced economic sustainability |
| Quality Assurance | - Regular follow-ups - Group sessions | - Supervision by trained personnel and UNICFE and partner staff | - Effective intervention implementation |
| Monitoring & Evaluation | - Tracking food security & livelihood progress | - Periodic reporting by implementing partners and partially through process documentation by the research firm | - Evidence-based intervention improvements |
| Local government involvement | - DLOs, ULOs, SALOs, Department of Women Affairs involvement during implementation | - UNCC and DNCC meeting | - Implementation sustainability |
