# Supplementary material for: Integrated nutrition-sensitive interventions are associated with improvements in household food security and dietary diversity among vulnerable women in the host communities of Cox’s Bazar, Bangladesh: Findings from a pre-post comparison study
Source: PLoS One. 2026 Nov 5;21(11):e0362124. doi: 10.1371/journal.pone.0362124 (PMC13739155; doi:10.1371/journal.pone.0362124)
Supplement: Supplementary file 5 [file pone.0362124.s008.docx]

**Manuscript ID: PONE-D-26-04366**

**Manuscript title:** Integrated nutrition-sensitive interventions are associated with improvements in household food security and dietary diversity among vulnerable women in the host communities of Cox’s Bazar, Bangladesh: Findings from a pre-post comparison study

**Reviewers 1**

**Reviewer #1: Overall assessment**
This manuscript responds to an important applied question for humanitarian and fragile contexts by asking whether a multisectoral package of nutrition-sensitive income-generating activities (either backyard poultry rearing or tailoring), cash transfers, and nutrition education was associated with improved household food security and women’s dietary diversity in vulnerable women-headed households in Cox’s Bazar. The subject matter is within the scope of PLOS ONE because it is policy-relevant and likely generalizable to similar contexts. The manuscript is overall well-motivated, intervention description is concrete, and the use of multiple analytical approaches (standard regression, inverse probability weighting, and propensity score regression) represents a strength.
But the way this is currently designed and framed raises huge concerns about causal interpretation. The analysis compares 2 repeated cross-sectional (2022 vs 2024) samples of program beneficiaries and uses survey year as a proxy for exposure to the intervention. With no contemporaneous comparison group or clearly specified baseline pre-exposure to the program, results should be viewed as changes in time amongst those exposed to the program rather than representing an ‘impact’ estimate. Another domain requiring correction to ensure interpretability and reproducibility is several reporting and coding issues (the direction of binary outcome coding, terminology around matching vs weighting). Finally, addressing the main points below would substantially improve your manuscript.

**Response:** Thank you for your thoughtful and constructive assessment. We appreciate your recognition of the study’s policy relevance, the clear intervention description, and the use of multiple analytical approaches. We agree with your concerns regarding causal interpretation and reporting clarity, and we have revised the manuscript to frame the findings more cautiously as changes over time among exposed households, clarified the analytic terminology and coding, and addressed the specific issues raised.

**Major comments**
1) Specify the evaluation design, estimand and causal language
The paper repeatedly refers to an ‘impact’ evaluation, but the design is closer to a pre–post (or early–late) comparison among beneficiaries. Both samples were selected from the same list of 2,000 beneficiaries and initiation of the intervention in 2021 makes it difficult to say that the sample for 2022 is a clear baseline pre-intervention. Estimate: (a) expose clearly the exposure variable (e.g., months since receiving assets/cash/training); (b) specify the estimand (average treatment effect among beneficiaries, change in prevalence over time, and so on); and (c) amend claims of causality accordingly; should not use ‘causes,’ say that is ‘associated with’ or ‘turns over time ± unless stronger identification were made. If authors want to make a stronger causal inference, then I would recommend introducing an analytic cohort for comparison (non-beneficiaries from similar unions or sub-districts) or consider using quasi-experimental designs—such as difference-in-differences if pre-intervention baseline data is available or interrupted time series with multiple pre/post points).

**Response:** Thank you for your valuable comment. We agree that the original wording overstated the strength of our study design. We have revised the manuscript to describe the study as a pre-post comparison study among beneficiaries rather than an impact evaluation. Please see the title in page 1. We have also removed causal language throughout the manuscript and replaced terms such as “causes” and “impact” with more appropriate language, such as “associated with,” “changes observed over time,” and “differences between survey rounds.” It has been modified in the overall results section. We agree that the exposure variables should be stated more clearly. In our study, all beneficiaries received the same type of program support, including cash/assets/training; however, the support was provided in different months and years according to Upazila, which means that in the same Upazila, all beneficiaries received support on the same day. The sub-district/study area variable has already been added in the propensity score analysis; therefore, the duration of exposure is already adjusted. Regarding the estimand, we now state more explicitly that our analysis estimates changes in outcome prevalence and associated factors between the two survey rounds among programme beneficiaries, rather than an average treatment effect or a causal programme effect. Please see the method section page 9, lines 183-186; page 10, lines 213-220 and page 15, lines 295-307.

2) Discrepancies between interpretations and outcome definitions/coding
The text construes binary outcomes in a reversed way when it comes to the interpretation of odds ratios. For HFIAS, the manuscript says households were recategorized into 1 = moderately/severely food insecure and 0 = food secure/mildly food insecure but then interprets OR>1 meaning more likely to be food secure. Similarly, here, dietary diversity is defined as 1 = insufficient DD (<5 food groups), however OR>1 interpreted that there is better DD. Thank you for being sensitive to the labels, which should match the modeled outcome. And since the ORs are very central to what the conclusions are, this is a major correctness issue.

**Response:** Thank you for pointing this out. We sincerely apologize for this mistake. We have corrected the outcome variables. For outcome variables, we explored household food security and women’s dietary diversity (DD). For the HFIAS, we categorized it into two groups: 0 = moderately/severely food insecure as food insecure and 1 = food secure, which includes food secure/mildly food insecure. This binary categorization has been followed by other studies and supports other valid documents; references have been added to the manuscript. Please see the revised lines with the correct references on page 12, line 256-262. In addition, we defined dietary diversity as 0 = inadequate DD (<5 food groups) and 1 = adequate DD (≥5 food groups). See page 13, lines 270-273.

3) Lack of methodological reporting of propensity scores and inconsistent use of terminology
Abstract and Methods refer to propensity score matching, but Tables and text report weighted data (after assigning weight’) and later also report PS regression. Exactly what was done: (i) PS model specification (which variables, functional forms, interactions, etc.), (ii) matching if used (algorithm/caliper/replacement/matching ratio), or weighting if used (stabilized weights/trimming), diagnosis for pre/post-matching imbalance between groups/standardized mean differences for all covariates with cutoff threshold/plots, and how uncertainty was computed under weighting-robust/HC standard errors. Whenever matching is not done, change ‘matching’ to ‘weighting’ everywhere. Also, whether the estimand is marginal (ATE/ATT) and what has to do with the three models that are reported.

**Response:** Thank you for your valuable comment. We have revised the Abstract, Methods, Tables, and Results sections to ensure consistent terminology and clearer methodological reporting. Specifically, we stated that our analysis used inverse probability weighting (IPW) based on propensity scores rather than propensity score matching. No matching procedures were performed.

We clarified the propensity score model specification in the methods section by identifying the covariates included in the treatment model: age, marital status, education level, occupation, household income, type of IGA assistance, wealth quintile, and sub-district/study area. We also specify how these variables were entered in the model, for example, categorical/continuous.

We further clarify that the target estimand is the marginal average treatment effect (ATE), and that this was estimated on the risk ratio scale using a modified Poisson regression model with stabilized inverse probability weights. Please see page 2, lines 33-39; page 15-17, lines 291-347.

4) Temporal confounding, secular shifts and seasonality are significant threats to validity
Seasonality in food access and diet could account for some of the observed differences, because timing of survey differed (first round Aug 2022–Mar 2023; second round Mar–Jun 2024) and outcomes depend on recent recall periods (HFIAS past 4 weeks; MDD-W past 24h). And From 2022 to 2024, macroeconomic shocks and price changes cause shifts in food security regardless of the program. Please take care to include month/season controls (or a sensitivity restricting to comparable months), contextual data (e.g., local food price index if you have it, as relevant for your data in context), and discussion that sets explicit boundaries around what the study can or cannot attribute to the intervention.

**Response:** Thank you for your valuable comment. We agree that temporal confounding, seasonal variations in food access and diet, and broader macroeconomic changes between survey rounds may influence the observed differences. Unfortunately, our dataset does not contain direct measures of month-specific seasonality, local food price indices or other contextual market indicators. To address this limitation, we have revised the manuscript in two ways as follows. First, we softened the causal language and reframed the findings as associations observed across two survey rounds rather than effects attributable solely to the intervention. Second, we have added a clear limitation in the Methods and Discussion sections, acknowledging that differences in timing, recall windows, seasonal food access, prices, and economic conditions may have contributed to changes in the HFIAS and MDD-W outcomes. Please see the revised and newly added lines on page 15, lines 299-302, and page 27-28, lines 528-538.

5) Consider the exposure and ‘dose’ of the intervention rather than using survey year as a proxy
The intervention package includes multiple components (training, assets, cash transfers in six installments, supervision, nutrition education). If the program rollout was not uniform, beneficiaries in 2022 may have had different intensity/duration of exposure than those in 2024. If data exist, incorporate direct measures: time since training/asset receipt, number of follow-up visits attended, cash installments received, participation in nutrition education, and program fidelity indicators. Even a simple stratified analysis by exposure duration would strengthen interpretation and align better with the ‘process evaluation’s framing

**Response:** Thank you for this important comment. In our study, all beneficiaries received the same type of program support, including cash/assets/training; however, the support was provided in different months and years according to Upazila, which means that in the same Upazila, all beneficiaries received support on the same day. The sub-district/study area variable is already added in the propensity score analysis, so here, the duration of exposure is already adjusted. However, we did not assess participation intensity in nutrition education sessions and, therefore, could not examine variations in engagement with that component. Consequently, we were unable to conduct dose-response or stratified analyses based on exposure duration. We have revised the manuscript to frame the analysis in terms of the intervention effect rather than the survey year, and we have added this as a limitation in the discussion section.

6) Consider effect measures more interpretable than odds ratios for common outcomes
Dietary diversity adequacy appears to reach ~86% in 2024; with common outcomes, odds ratios can exaggerate relative differences. Consider reporting risk ratios or prevalence ratios using log-binomial or modified Poisson models with robust SEs, alongside absolute differences in prevalence. PLOS ONE readers often value absolute changes (percentage-point differences) for policy translation.

**Response:** Thank you for your suggestion. Accordingly, we revised the analysis. The intervention effect was estimated using modified Poisson regression and inverse probability of sampling weighting (IPSW). Table 4 has been revised to reflect these updated analyses (page 23-24). We have also revised the corresponding results section; please see pages 22 to 23, lines 421-441.

**Minor comments and technical edits**
• Figures seem mislabeled: the figure that is labeled Fig 1 in the PDF appendix appears to be food-group consumption, while the figure labeled Fig 2 seems to show food security categories. Their figure files and captions should match the in-text citations, with clear panel labels if Fig 1 is meant to reflect both food security and dietary diversity.

**Response:** Thank you for pointing this out. The labels in Figures 1 and 2 have been mistakenly interchanged. We have corrected this error in the revised manuscript. We apologize for this oversight. In the new version, we have three figures. The previous 1 no figure will be figure 3, and 2 no figure will be figure 2.

• Clarify coding for marital status: Table 2 reports very low percentages ~9–13% for ‘married’. It may be so due to women-headed household eligibility, but the readers will challenge it. Explain briefly in Methods or Results.

**Response:** Thank you for your valuable comment. The low proportion of married participants reflects the intervention eligibility criteria, which primarily targeted economically vulnerable women from households with no stable source of income or those relying on irregular or casual labor employment. Consequently, the study population was drawn from a highly specific vulnerable group, which helps explain the marital status distribution shown in Table 2. We have added a brief clarification in the methods and results section to assist readers in interpreting these findings. We have mentioned the eligibility criteria in the methods section (please see page 7, lines 143-149).

• In Table 1 and main text, streamline terminology: ‘food security’ or at least use ‘food insecurity’; ‘adequate’ vs ‘inadequate’ dietary diversity. Table 1. Coding should follow the example manner of hitting the narrative.

**Response:** Thank you for your helpful suggestion. We have revised the terminology in Table 1 and throughout the main text to ensure consistency with the text. For outcome variables, we explored household food security and women’s dietary diversity (DD). For the HFIAS, we categorized it into two groups: 0 = moderately/severely food insecure as food insecure and 1 = food secure, which includes food secure/mildly food insecure. This binary categorization has been followed by other studies and supports other valid documents; references have been added to the manuscript. Please see the revised lines with the correct references on page 12, line 256-262. In addition, we defined dietary diversity as 0 = inadequate DD (<5 food groups) and 1 = adequate DD (≥5 food groups). See page 13, lines 270-273.

• Some Discussion statements are too strong for the design (e.g., ‘strong empirical evidence’ and ‘it can be claimed’). Detone provides to even emphasize a basic association and talk about residual confounding.

**Response:** Thank you for this helpful observation. We agree that some statements in the Discussion section were stronger than those warranted by the study design. We have revised the manuscript to use more cautious language throughout and avoid overstating the findings. Specifically, terms such as ‘strong empirical evidence’ and ‘it can be claimed’ have been replaced with wording that emphasizes observed associations only. Please see page 26, lines 506-509, and page 29, line 561.

• Reword the sentence saying that propensity score methods are ‘better than’ logistic regression. Under certain conditions PS methods may reduce model dependence, but they are not panacea.

**Response:** Thank you for this important comment. We agree that the earlier wording was too strong and could have been misleading. In the revised Discussion section, we removed the statement suggesting that propensity score methods are better than logistic regression. Instead, we now describe propensity score weighting more cautiously as an alternative analytic approach that can help improve the balance in observed covariates and reduce model dependence under certain assumptions, but not as a universally superior method. This revision is consistent with the updated Methods section, where we clarified that our analysis used inverse propensity score weighting with modified Poisson regression for outcome estimation. Please see page-26, lines 519-523.

• If possible, report exact p-values; but goals guide readers to effect sizes and CIs. Do not overemphasize statistical significance alone.

**Response:** Thank you for your helpful suggestion. We agree that effect sizes and confidence intervals should be emphasized over statistical significance. In the revised manuscript, we added the standard errors for the estimated risk ratios to improve the transparency of the results. We have also revised the presentation of the findings to focus more clearly on the magnitude and precision of the estimated associations rather than relying only on statistical significance.

• More intervention implementation details relevant to generalizability: who delivered nutrition education, frequency, curriculum and whether participation was mandatory/optional.

**Response:** Thank you for your helpful suggestion. We agree that additional details on intervention implementation are important for assessing generalizability. We have now clarified the key implementation characteristics of the nutrition education component, including who delivered the sessions, the frequency of delivery, the curriculum content, and the nature of participation. A detailed description of these elements is provided in Supplementary Table S1, which expands on the intervention description presented in the main text. We have also added a brief summary in the Methods (Intervention description) section and referred readers to Supplementary Table S1 for complete details.

• Include STROBE checklist (observational designs) and TIDieR checklist [29] elements for intervention reporting.

**Response:** Thank you for suggesting. We have included this checklist in supplementary files (S3 and S4 files).

• Minor language/typo checking: ‘background poultry’ must be ‘backyard poultry’; consistent spelling of Cox’s Bazar; unit/exchange rate verification (using an August 2025 converter for a 2021–2024 programme may baffle the audience).

**Response:** Thank you for your suggestion. We have corrected the typo “background poultry”; please see page 25, line 475 of the revised manuscript. We have also made the spelling of Cox’s Bazar consistent throughout the manuscript, including the correction in page 7, line 136. Finally, we retained the USD conversion rate as of January 1, 2021. Please see the revisions on page 7, lines 145-147, and page 9, line 183.

• Confirm whether individuals could be sampled in each round. If yes, adjust for possible repeated measures or clustering; if not, describe how duplicates were prevented

**Response:** Thank you for this important comment. The two survey rounds were based on independent samples, and the study was not designed to follow the same individual over time. Participants in each round were selected separately according to the sampling framework. Therefore, repeated measurements at the individual level were not expected, and adjustments for within-person correlations were not applicable. We have clarified this in the methods section of the revised manuscript. Please see page-11, lines 229-230.

**Reviewers 2**

Reviewer #2: The manuscript addresses an important issue and presents encouraging findings from a highly relevant setting. It has clear practical significance, and the intervention package appears meaningful for vulnerable women in Cox’s Bazar. However, the manuscript would benefit from several improvements, as outlined in the following specific comments:
1. The background would benefit from more context from studies conducted in similar settings as Cox’s Bazar or comparable humanitarian or low-resource settings. In particular, it would strengthen the introduction to discuss what is already known about nutrition-sensitive interventions such as tailoring, poultry rearing, or other income-generating activities, including their sustainability and their relevance to this setting.

**Response:** Thank you for your suggestion. We have already included a discussion in the introduction on nutrition-sensitive interventions in similar humanitarian and low-resource settings, including income-generating activities such as poultry rearing and tailoring, as well as their relevance to Cox’s Bazar. We have carefully checked this section and clarified the wording to make the context more explicit. Please see page: 4-6 line: 71-85, 92-95, 103-112.

2. The interpretation of the findings should be more cautious and avoid strong causal wording. The study compares two independently sampled cross-sectional groups from 2022 and 2024, and the time variable was used as a proxy for intervention exposure. This design supports an association over time, but not strong causal attribution. Therefore, statements such as “The substantial improvements were likely driven by structured IGA implementation, with asset transfers, direct cash support, and nutrition education” in the abstract may need to be softened and replaced with wording that stays closer to the observed findings. This applied to the discussion and conclusion as well.

**Response:** Thank you for this important comment. We agree that the interpretation of our findings should be more cautious and should not imply strong causal attribution, given the repeated cross-sectional design and the use of time as a proxy for intervention exposure. In the revised manuscript, we have softened the language in the abstract, discussion, and conclusion sections and removed statements that could be interpreted as causal.

Specifically, we now state: “Our study provides evidence of an association between nutrition-sensitive interventions coupled with nutrition education and improved household food security and dietary diversity among vulnerable women in low-income Bangladesh, although residual confounding cannot be excluded.” This revised wording more accurately reflects the observational nature of the study and the limitations of causal inference. Please see page 29, lines- 561-564.

3. Please elaborate more clearly on how the propensity score approach was used. The manuscript uses terms such as propensity score matching, weighting, IPW, and PS regression, but these are not presented consistently across sections. It would be helpful to use consistent terminology throughout and explain more clearly why this approach is appropriate when comparing survey rounds rather than exposed versus unexposed groups.

**Response:** Thank you for this important comment. We have revised the Abstract, Methods, Tables, and Results to ensure consistent terminology and clearer methodological reporting. Specifically, we now state throughout that our analysis used inverse probability weighting (IPW) based on propensity scores, rather than propensity score matching. No matching procedure was performed.

We clarified the propensity score model specification in the methods section by identifying the covariates included in the treatment model: age, marital status, education level, occupation, household income, type of IGA assistance, wealth quintile, and sub-district/study area. We also specify how these variables were entered in the model, for example, categorical/continuous. Please see page 15-17, lines 308-347.

4. Since all participants were beneficiaries and there is no non-beneficiary control group, it is difficult to separate intervention-related change from secular trends, recovery over time, or other contextual influences. This should be discussed more explicitly as a major limitation of the study design.

**Response:** Thank you for your suggestion. We have added this to the limitations in the discussion section (page 27, lines 528-538.

5. Table 2 shows the covariate distribution before and after weighting. The presentation would be stronger if the authors reported standardized mean differences more explicitly for all variables.

**Response:** Thank you for this valuable suggestion. We agree that standardized mean differences for all covariates would strengthen the assessment of balance before and after the weighting. However, these diagnostics were not included in the revised manuscript. We acknowledge this as a limitation and note that Table 2 presents the covariate distributions before and after weighting as a descriptive indication of sample balance.

6. The first round of the survey was conducted from August 2022 to March 2023, while the second round was conducted from March to June 2024. Please discuss the different timing of the two survey rounds and whether seasonal variation may have influenced food security and dietary diversity outcomes.

**Response:** Thank you for this important comment. We agree that the different timing of the two survey rounds may have influenced household food security and dietary diversity because of possible seasonal variations in food availability, income, expenditure patterns, and consumption practices. In the revised manuscript, we have clarified the timing of both survey rounds in the Methods section and added a note in the discussion/limitations section to acknowledge that the surveys were not conducted in the same calendar period. This difference occurred because of operational constraints, including administrative permission delays and a fixed project timeline, which required the completion of data collection within the implementation period. We now explicitly acknowledge that seasonal differences may have affected the observed outcomes and that this should be considered when interpreting the changes between the two survey rounds. Please see page-15, lines 299-302 and page- 26-27, lines 528-538.

7. Some parts of the discussion go beyond what was directly measured. For example, the discussion refers to nutrition knowledge (line 420) or empowerment (line 428-428), but it is not clear whether these variables were included in the final analysis. If such variables were captured in the questionnaire, the authors may consider incorporating them into the analysis or at least clarifying this point. Including findings on knowledge-related measures, if available, could make the discussion more convincing.

**Response:** Thank you for pointing this out. In the Discussion section, we noted that alongside improvements in food security and dietary diversity, women’s empowerment and nutrition knowledge may also have improved. In the second survey round, 93.35% of women reported improved decision-making power regarding the purchase of food and daily household essentials. However, we did not formally examine these variables in the present study. In addition, decision-making power could not be included in the model because of its highly uneven distribution, which affected model stability and the final estimates. To avoid confusion and overinterpretation, we have removed this paragraph from the revised manuscript. Please see the revised discussion section, page-25, lines 491-502.

8. It may also be useful to consider whether women’s living arrangement or household decision-making context could influence food selection and dietary diversity. If such information was collected, the authors may want to explore whether it should be included among the explanatory variables.

**Response:** Thank you for your helpful suggestion. We considered the household economic position in the analysis through the wealth quintile variable, which partly reflects differences in household living conditions. Therefore, living arrangement-related variation was addressed indirectly in the model and was not presented as a separate explanatory variable.

Information was collected regarding the household decision-making context. However, in the 2024 survey round, a very high proportion of women (93.35%) reported that they made decisions regarding food purchases and daily household essentials, spending earned money, etc. Owing to this highly uneven distribution, the variable provided very limited variability and was not suitable for inclusion in the adjusted model.

9. The title is somewhat long and could be made more concise.
**Response:** Thank you for this suggestion. We agree that the original title was somewhat lengthy. The title has been revised for clarity. Please see the revised title on page 1.

10. Since the manuscript includes comparative outcome analysis, the term “process evaluation study” in the title may not fully reflect the analysis presented. The authors may consider terms such as “repeated cross-sectional evaluation” or “program evaluation” instead.

**Response:** Thank you for your helpful suggestion. We agree that the term “process evaluation study” does not fully reflect the comparative outcome analysis presented in the manuscript. In line with the reviewer’s suggestion and to better represent the study design, we have revised the title to: “Integrated nutrition-sensitive interventions are associated with improvements in household food security and dietary diversity among vulnerable women in the host communities of Cox’s Bazar, Bangladesh: Findings from a pre-post comparison study.” We believe that this revised title more accurately reflects the analytical approach and scope of the study. Please see the revised title on page 1.

11. Please elaborate more clearly on the rationale for the dietary diversity classification, including why the chosen cut-off was used and how it was validated or supported by other literature.

**Response:** Thank you for your helpful comments. The rationale for the dietary diversity classification was already described in the Methods section using the Minimum Dietary Diversity for Women of Reproductive Age (MDD-W) framework with supporting references. In the revised manuscript, we have further clarified that the cutoff of ≥5 out of 10 food groups is the standard MDD-W threshold used to indicate adequate dietary diversity and better micronutrient adequacy among women of reproductive age, while consumption from <5 food groups was classified as inadequate dietary diversity. Please see page-12-13, lines 263-273.

12. Similarly, please clarify if the Household Food Insecurity Access Scale (HFIAS) has been validated in Bangladesh context.

**Response:** HFIAS is a standard scale used in the Food Security and Nutrition Surveillance Project in Bangladesh. Although this binary classification has not been specifically validated in the Bangladeshi context, it is widely used internationally to categorize household food insecurity. We have now included the relevant references in the manuscript; please see page 12, lines 256-262.

13. Current figures and tables would benefit from clearer presentation. Please indicate whether the percentages shown are weighted or unweighted, and ensure that each figure and table can be understood without requiring extensive reference back to the main text.

**Response:** Thank you for your helpful suggestion. We confirm that all percentages presented in the tables and figures are not weighted. We have now revised the table and figure legends to explicitly state this. In addition, we have improved the clarity of all tables and figures by adding clearer titles, labels, and footnotes so that each can be interpreted independently, without requiring reference to the main text.

**Reviewers 3**

**Reviewer #3: General Comment**
This manuscript addresses an important topic, assessing the impact of integrated nutrition-sensitive interventions on household food security and dietary diversity among vulnerable women in Cox’s Bazar, Bangladesh. This study has the potential to provide valuable insights. However, there are several methodological and reporting issues that require clarification to enhance transparency, reproducibility, and consistency throughout the manuscript.

**Specific Comments**

**1. Abstract (Lines 19–20):**
The phrase “underlying causes of nutrition” appears to be incorrect. It would be more accurate to state “underlying causes of malnutrition” to convey the intended meaning.

**Response:** Thank you for your suggestion; it has been revised. please see page-2, line-21.

**2.** **Sample Size Calculation (Line 192):**
The statement “The sample size was calculated using the standard formula” is vague. The authors should specify the formula used and clarify what is meant by the “prevalence of 0.5.” Is this referring to the prevalence of food insecurity or inadequate dietary diversity or something else? A reference supporting this prevalence estimate should also be provided. Moreover, there are two outcome variables with two different prevalences, which one was used and why?

**Response:** Thank you for this important comment. We agree that the previous description of the sample size calculation was unclear. We have now revised the Methods section to specify that the sample size was calculated using the single-population proportion formula, n = Z^2^ p(1−p)/d^2^. The assumed prevalence of 50% (p = 0.5) was not intended to represent the observed prevalence of a specific study outcome; rather, it was used as a conservative assumption because no reliable prior estimate was available for the study population. This approach was chosen because a prevalence of 50% yielded the maximum sample size. We have clarified this in the manuscript and added relevant references. Please see page 10, lines 200-206. Thank you for your suggestion. We included two outcome variables because they reflect two related but distinct dimensions of nutritional well-being. Household food insecurity captures household-level access to food, whereas women’s dietary diversity reflects the quality and variety of food consumed at the individual level. Both outcomes are important for understanding the broader nutrition situation among vulnerable women and assessing the potential association of the intervention with household food access and women’s diet quality.

**3. Sampling Description (Lines 207–208):**
The phrase “randomization was conducted independently for each round” is misleading, as this is not a randomized trial. It is likely that the authors meant that participants were randomly selected independently for each round. Clarification is needed.
**Response:** Thank you for observing this important point. We have revised the line to indicate that the participants were randomly selected (page-10, line 218-220).

**4. Sampling Procedure:**
The manuscript states that participants were “randomly selected,” but the procedure is not described in detail. The authors should clarify:
• Whether simple random sampling or stratified sampling was used.
• Whether sampling was stratified by intervention type (backyard poultry vs. tailoring).

**Response:** Thank you for your comment. Participants were selected using simple random sampling method. The sampling was not stratified by intervention type (e.g., backyard poultry rearing or tailoring). We have clarified this in the Methods section to provide a more detailed description of the sampling procedures. Please see page 10, lines 214-215.

5. Re-interviewed Participants (Second Round, 2024):
The authors should report the proportion of participants in the second round who were re-interviewed from the first round. This information is important for clarifying the independence of the two survey rounds in this repeated cross-sectional study.

**Response:** Thank you for this important comment. The surveys were designed as independent cross-sectional rounds, and participants were selected separately in each round according to the sampling framework. Unique identifiers were not collected to link individuals across rounds; therefore, it was not possible to determine whether any participants in the second round also participated in the first round. Consequently, the analysis treated the two samples as independent samples. We have clarified this in the Methods section. Please see page-10, lines 218-220.

**6. Outcome Variable Coding and Consistency:**
There is inconsistency in reporting and coding of the outcome variable:
• In Line 220, the primary outcome is described as “household food insecurity.”
• In Table 1, it is presented as “household food security,” coded as 1 = moderate to severe food insecurity and 0 = food secure to mildly food insecure.
• Line 263 indicates that the “food insecurity category” was treated as the reference category.
For example, in binary logistic regression, the reference category is typically coded as 0, while the event category is coded as 1. Clarification is needed on which category was treated as the reference in the regression analysis, and whether the interpretation of odds ratios aligns with the coding. The authors are encouraged to harmonize the terminology, coding scheme, and regression interpretation to ensure methodological clarity and consistency.

**Response:** Thank you for pointing this issue, we apologies for the mistake. We have corrected the outcome variables. For outcome variables, we explored household food security and women’s dietary diversity (DD). For the HFIAS, we categorized it into two groups: 0 = moderately/severely food insecure as food insecure and 1 = food secure, which includes food secure/mildly food insecure. This binary categorization has been followed by other studies and supports other valid documents; references have been added to the manuscript. Please see the revised lines with the correct references on page 12, line 256-262.

**7. Propensity Scores:**
The manuscript would benefit from a brief explanation of propensity scores in the Methods section, including what they are and how they should be interpreted in the context of the analysis. This will help readers better understand their role in the study.

**Response:** Thank you for your comments. The method section is revised, please see page 15-17.

**8. Figures 1 & 2:**
It appears that Figures 1 and 2 may have been inadvertently interchanged. Figure 1 does not show the changes in household food security as indicated in the footnote (Line 307), suggesting a possible mix-up. The authors should review the figures and ensure that each figure and its legend accurately correspond to the data presented.

**Response:** Thank you for pointing this out. The labels in Figures 1 and 2 have been mistakenly interchanged. We have corrected this error in the revised manuscript. We apologize for the oversight.

**9. Accuracy of Data Presentation:**
The authors should ensure that all statements referring to figures or tables accurately reflect the data presented. For example, Lines 301–303 state:
“A significant improvement in food security status was observed among vulnerable women between 2022 and 2024 (Fig 1). The prevalence of household food security among vulnerable women improved substantially, from 9% in 2022 to 34% (p<0.001) in 2024.”
However, Figure 1 does not display p-values, which suggests that these may have been derived from a table that is not referenced here. The authors should clarify the source of the statistical results and ensure that the text accurately points to the corresponding figure or table throughout the Results section.

**Response:** Thank you for pointing this out. The labels in Figures 1 and 2 have been mistakenly interchanged. We have now corrected this error in the revised manuscript. We apologize for the oversight.

**10. Conclusion:** Statements in the conclusion that are not directly supported by the study findings should be avoided. For example, the statement: “Sustained investment, strong political commitment, and intersectoral collaboration are essential for scaling and institutionalizing such approaches within national development frameworks” does not appear to be directly derived from the results presented in this study. Conclusions should be grounded in the study’s empirical findings. In scientific writing, conclusions that extend beyond the data may be considered speculative and can weaken the overall rigor of the manuscript. The authors are encouraged to revise the conclusion to ensure it aligns closely with the evidence generated by the study.

**Response:** Thank you for this important comment. We agree that the statement on sustained investment, political commitment, and intersectoral collaboration extends beyond the empirical findings of the present study. In response, we have revised the conclusion to remove this broader policy-oriented statement and ensure that the concluding remarks are more closely aligned with the evidence generated by the study. Please see page-29, lines 569-572.

**Reviewers 4**

**Reviewer #4:** Thank you for giving me the opportunity to review an interesting paper. The study provides valuable evidence on an important humanitarian issue. With a clear revision to address the confusion between "process evaluation" and "impact evaluation," as well as a clarification of the limitations regarding time-varying confounders, this manuscript would be a strong contribution to literature. I recommend Minor Revision.
**Comment 1.** Consistency with the title and studied outcomes: The manuscript describes an impact evaluation (measuring food security and dietary diversity outcomes using propensity score matching) while its title states "process evaluation study”. A process evaluation typically focuses on implementation fidelity, reach, participant experience, and barriers to delivery. The current manuscript provides little on the process of the intervention (e.g., implementation challenges, participant attendance, and fidelity to training modules). I recommend either updating the title and scope to "Impact Evaluation" or significantly expanding the manuscript to include actual process evaluation metrics.

**Response:** Thank you for this important comment. We agree that the term “process evaluation study” did not accurately reflect the primary focus of our analysis, which is a comparative assessment of outcomes. In response, we have revised the title to better align with the study design and analytical approach: “Integrated nutrition-sensitive interventions are associated with improvements in household food security and dietary diversity among vulnerable women in the host communities of Cox’s Bazar, Bangladesh: Findings from a pre-post comparison study.”

We also reviewed the manuscript to ensure consistency between the title, objectives, and reported outcomes. The revised title removes the implication of a process evaluation and more appropriately reflects the outcome-focused analysis conducted using propensity score methods. Please see the updated title on page 1.

**Comment 2.** Logic model presentation: Given that the authors claim this is a process evaluation, the lack of a visual logic model is a significant gap. A logic model would clarify the pathway from intervention (backyard poultry/tailoring/cash) to intermediate outcomes (household income) to final outcomes (food security/dietary diversity). I strongly suggest the authors include a conceptual framework or logic model diagram to illustrate how the integrated interventions were hypothesized to impact outcomes.

**Response:** Thank you for this valuable suggestion. We agree that a logic model helps clarify the hypothesized pathways linking interventions to outcomes. We have now included a conceptual framework (Fig 1) in the Methods section to illustrate the pathway from the integrated interventions (backyard poultry, tailoring, and cash support) to intermediate outcomes (e.g., household income and knowledge) and final outcomes (food security and dietary diversity). This addition improves the clarity of the process-evaluation framework.

**3. Intervention groups:** The authors acknowledge they could not independently examine the effects of backyard poultry rearing vs. tailoring due to sample size, so they combined them. This aggregation assumes that both interventions operate through similar mechanisms to produce similar magnitudes of effect. The authors should provide brief justification for why these are considered sufficiently similar to combine or discuss this as a key limitation.

**Response:** Thank you for this important comment. We agree that combining backyard poultry rearing and tailoring into a single intervention category requires further justification. These two intervention arms were combined because both were implemented within the same overall program framework and designed to support women’s livelihoods through small, home-based income-generating activities. In both groups, participants received resources to initiate or strengthen home-based livelihoods, financial support for their activities, and education on nutrition and hygiene practices. The main difference between the two groups was the specific type of income-generating activity: poultry rearing or tailoring. Given these shared program components and the limited sample size within the individual categories, we considered it reasonable to combine them for the analysis. We have now clarified this rationale in the manuscript’s method section and noted the inability to assess their independent effects as a study limitation.

**4. Confounding:** The study uses two cross-sectional rounds (2022 and 2024). While propensity score matching addresses baseline differences between the populations sampled, it does not fully control for external, time-varying confounders (e.g., changes in the local economy, food prices, or other NGO activities in the area between 2022 and 2024). The authors should acknowledge this in the discussion as a potential threat to the attribution of causality to the specific intervention.

**Response:** Thank you for this important comment. We agree that although propensity score matching improves comparability between the intervention and comparison groups based on observed covariates, it cannot fully account for unmeasured or time-varying contextual factors operating between the 2022 and 2024 survey rounds. Changes in the local economy, food prices, weather conditions, government programs, or the activities of other NGOs may have also influenced household food security during the study period. We have now acknowledged this more explicitly in the discussion (page 27-28, lines 528-538).

**1. Terminology (Page 11, line 228):** In the definition of food insecurity, you state "1 for moderately to severely food insecure, and 0 for food secure to mildly food insecure." Please clarify the rationale for grouping "mildly food insecure" with "food secure".

**Response:** Thank you for your comment. We agree that the rationale for grouping mildly food-insecure households with food-secure households must be clarified. Following prior applications of this multidimensional classification approach, we dichotomized the outcome into food secure/mildly food insecure versus moderately/severely food insecure to create a simpler, policy-relevant contrast for estimating treatment effects. This recategorization was intended to distinguish households experiencing more substantial deprivation from those with no or relatively limited food insecurity. We have revised the manuscript accordingly. Please see page 12, lines 255-262.

2. Authors note that 5% of interviews were re-interviewed. It would be helpful to briefly state the results of these quality checks—were there high discrepancies.

**Response:** Thank you for your helpful comments. As part of the data quality assurance process, re-interviews were conducted only when discrepancies exceeded 25%. In our quality checks, the level of discrepancy was below this threshold and did not exceed 12%. These discrepancies were reviewed and corrected through discussions with the research assistants and respondents within 48 hours of data collection. We have now clarified this in the manuscript; please see page 11-12, lines 237-243.

3. Table 1. Type of IGA assistance" is listed as an explanatory variable, yet the study mentions combining them due to sample size. Please clarify if this was used in the propensity score calculation or the final model.

**Response:** Thank you for your comment. Due to the small sample size in the individual IGA categories, the type of IGA assistance variable was combined into broader categories before the analysis. The combined variable was then included in the models. We have clarified this in the methods section. Moreover, we have clarified this in the Methods section (page-13, lines 278-283.
