# Supplementary material for: Integrated nutrition-sensitive interventions are associated with improvements in household food security and dietary diversity among vulnerable women in the host communities of Cox’s Bazar, Bangladesh: Findings from a pre-post comparison study
Source: PLoS One. 2026 Nov 5;21(11):e0362124. doi: 10.1371/journal.pone.0362124 (PMC13739155; doi:10.1371/journal.pone.0362124)
Supplement: Supplementary file 6 [file pone.0362124.s009.docx]

**Manuscript ID: PONE-D-26-04366R1**

**Manuscript title:** Integrated nutrition-sensitive interventions are associated with improvements in household food security and dietary diversity among vulnerable women in the host communities of Cox’s Bazar, Bangladesh: Findings from a pre-post comparison study

**Reviewers 1**

**Reviewer #1: Overall Assessment**

This revised manuscript evaluates the association between integrated nutrition-sensitive interventions and improvements in household food security and dietary diversity among vulnerable women in Cox’s Bazar, Bangladesh using repeated cross-sectional survey data and propensity score-based weighting methods.

The study addresses a highly relevant humanitarian and public health issue. The intervention is policy-relevant, and the dataset is valuable in a fragile and resource-constrained setting. The authors have made substantial improvements in response to prior comments, particularly in clarifying the study design, correcting methodological inconsistencies, and improving reporting transparency.

However, important methodological and inferential limitations remain that affect the strength of interpretation, particularly regarding causal claims, confounding control, and design limitations.

Major Strengths

1. Policy-relevant topic with strong humanitarian importance.

2. Improved methodological clarity (PS weighting, IPW, corrected terminology).

3. Appropriate use of modified Poisson regression for risk ratios.

4. Large sample size and real-world program data improving external validity.

Major Concerns

1. Causal inference remains overstated despite revisions. The design is repeated cross-sectional with no control group.

2. Residual confounding remains due to unmeasured and time-varying factors (seasonality, macroeconomic changes, NGO activities).

3. Exposure definition using survey year remains conceptually weak.

4. Lack of full standardized mean difference (SMD) balance diagnostics reduces transparency.

5. Effect size interpretation may be optimistic; absolute differences should be emphasized more.

Minor Concerns

1. Some sections still contain overly generalized interpretations.

2. Figures and tables improved but could be more self-explanatory.

3. Intervention fidelity details remain limited.

Overall Interpretation

The authors have significantly improved the manuscript. However, the study design limits causal inference, and findings should be interpreted as associations rather than effects.

The manuscript is suitable for publication after minor revision, provided interpretation is further moderated.

Recommendation

Minor Revision

**Key requirements:**

- Remove residual causal language

**Response:** Thank you for this valuable comment. We have carefully revised the entire manuscript to remove residual causal language and to present the findings more appropriately as associations rather than causal effects. The wording has been revised in several sections, including page 17, lines 337-340; page 19, line 368; the title of Table 3; page 22, line 414; and page 23, line 425. These revisions were made to ensure that the interpretation of the results is consistent with the study design.

- Strengthen discussion of limitations

**Response:** Thank you for your helpful suggestion. We have strengthened the limitations section in the discussion to more clearly acknowledge the methodological limitations of the study, including issues related to residual confounding and interpretation of the findings. Please see page 27, lines 503-505.

- Add balance diagnostics clarity if possible

**Response:** Thank you for this comment. We have now added a new figure 4 (love plot) showing the standardized mean differences (SMDs) for comparing covariate balance between two treatment groups. The figure demonstrates how weighting improved the balance of observed covariates between the comparison groups. We have also added a description of this figure in the results section. Please see pages 20-21, lines 385-391.

- Emphasize absolute effects alongside risk ratios

**Response:** Thank you for this useful suggestion. We have revised the analysis to include absolute effect estimates alongside the risk ratios. Specifically, we added two additional binomial models to present the estimated absolute differences in food security and dietary diversity. These results are now presented in the revised Table 4 and described in the results section on page 22, lines 413-416 and 425-427.

**Reviewers 2**

**Reviewer #2:** I thank the authors for the detailed response to the previous comments. Many points have been addressed satisfactorily. However, several of the original concerns remain unresolved, and I have identified a few additional issues that require attention before the manuscript is suitable for publication.

1. The authors stated they had softened causal language throughout the manuscript. However, in the submitted revision this language does not appear consistently, e.g + The Results section (lines 404 and 411) attributes findings to the intervention with the phrases "due to the intervention." Such passages must be revised to reflect the observational, associational nature of the study.

**Response:** Thank you for your comment. We have deleted the lines that used this type of causal language. Please see (page 19, lines 368-370), (page 22, lines 414), and (page 23, lines 420-424).

2. Similarly, line 338 states: "The prevalence of household food security among vulnerable women got relatively better, from 9% in 2022 to 34% in 2024." The phrase "got relatively better" is casual and does not meet academic writing standards. Please revise.

**Response:** Thank you for this observation. We agree that the phrase "got relatively better" was informal and not suitable for academic writing. Accordingly, we revised the sentence to use more precise and formal language. The revised text now reads:

” The prevalence of household food security among vulnerable women showed a substantial increase, rising from 9% in 2022 to 34% (p<0.001) in 2024.”

This change has been incorporated into the manuscript (page 17, line 336-344).

3. The response stated that the paragraph mentioning women's empowerment had been removed from the Discussion. However, lines 491 to 499 about the intervention remains: "…contributed to enhanced women's empowerment" and "likely facilitated their greater involvement in household decision-making processes". As noted previously, this goes beyond the empirical findings.
**Response:** Thank you for your comment. The statements referring to women's empowerment and involvement in household decision-making have been removed from the revised manuscript in the first revision, as they were not directly supported by the empirical findings. These changes are reflected in the clean version of the manuscript in both the first and current versions. Please see the removed part in the relevant discussion section of the revised manuscript (track changes version of the previous (R1) version), where we deleted it. Please see page-26, 493-499.

4. The abstract (line 41–42) reports RR=3.29 (95% CI: 2.88–3.77) for dietary diversity. However, RR=3.29 is the unweighted modified Poisson regression estimate. The primary result, and the one correctly reported for food security in the same abstract is the IPSW (weighted) estimate. For dietary diversity, the IPSW result is RR=2.78. Reporting the unweighted estimate in the abstract as the headline finding is inconsistent with the study's stated analytic approach.

**Response:** Extremely sorry for the inconvenience, and thanks a lot for pointing that out. We agree with your comment and have revised the relevant portion of the abstract. Please see page 3, lines 39-42.

5. References 16 and 30 cite URLs beginning with "chrome-extension://...". Readers and editors cannot follow these links. The authors must replace these with the actual publicly available URLs, DOIs, or complete archival source information.

**Response:** Thank you for your comment, and extremely sorry for overlooking it. We have revised the links. Please see page-31, line- 603-604 and page-32, line- 647-649.

**Reviewers 3**

**Reviewer #3:** Thank you for the opportunity to review the revised manuscript. The authors have addressed most of my comments satisfactorily. However, I believe that Comment 9 regarding the accuracy of data presentation has not been fully addressed.
In this particular comment, my main concern was not related to the numbering or labeling of Figures 1 and 2, but rather to the inconsistency between the narrative text and the data presented in the figures. The manuscript reports a statistically significant improvement in household food security (p<0.001) and refers readers to a figure 2 as supporting evidence. However, the figure itself does not present p-values or statistical test results. As a result, it remains unclear where the reported p-value originates and whether it is derived from a table or a separate statistical analysis.
While the authors indicate that the figure labels were mistakenly interchanged and have now been corrected, this does not resolve the underlying issue. I recommend that the authors explicitly identify the source of the statistical results and ensure that all references to figures and tables accurately correspond to the data being presented throughout the Results section.
Therefore, I consider this comment to be only partially addressed and would appreciate further clarification from the authors before a final decision is made.

**Response:** Thank you for this important clarification. We agree that the concern was related not only to figure numbering but also to the consistency between the narrative text and the statistical information presented in relation to the figures. We have now revised the Results section to clearly indicate that the figure presents unweighted percentage/proportion changes in food security and dietary diversity, whereas the reported p-values were obtained from the corresponding statistical comparison. To avoid ambiguity, we have added a note below the figure stating that percentages are unweighted sample proportions and that the between-group differences for food security status and dietary diversity status were statistically significant (p < 0.001). We have also checked the figure and table references throughout the results section to ensure that each statement corresponds accurately to the data being presented. Please see page 17, line 343-345.
