# Supplementary material for: Integrated nutrition-sensitive interventions are associated with improvements in household food security and dietary diversity among vulnerable women in the host communities of Cox’s Bazar, Bangladesh: Findings from a pre-post comparison study
Source: PLoS One. 2026 Nov 5;21(11):e0362124. doi: 10.1371/journal.pone.0362124 (PMC13739155; doi:10.1371/journal.pone.0362124)
Supplement: Supplementary file 7 [file pone.0362124.s010.docx]

**Manuscript ID: PONE-D-26-04366R2**

**Manuscript title:** Integrated nutrition-sensitive interventions are associated with improvements in household food security and dietary diversity among vulnerable women in the host communities of Cox’s Bazar, Bangladesh: Findings from a pre-post comparison study

**Reviewers 1**

**Reviewer #1: Overall Assessment of Reviewer #1**

My concerns have been substantially addressed, particularly regarding the study limitations, residual confounding, covariate balance, and presentation of absolute effects. However, I do not consider the response fully satisfactory yet. The main remaining problem is that the authors state that causal language has been removed, while several causal or intervention-effect expressions remain in the revised manuscript. Because the study uses repeated cross-sectional data without randomization or a concurrent control group, these expressions should be corrected before acceptance.

**Response:** Thank you very much for your careful review and constructive comment. We agree that some casual or informal language has been used in the manuscript. Therefore, we carefully reviewed the entire manuscript and revised the language to ensure a more appropriate academic and scientific tone. Details of these revisions are provided in our responses to the specific comments below.

**Comment 1:** Causal Interpretation and Residual Confounding

Assessment: Partially addressed. The authors have made a strong effort to acknowledge the limitations of the study design. The revised Discussion now recognizes residual confounding from unmeasured time-varying factors and explicitly notes that seasonal variation, food prices, dietary patterns, climatic shocks, local economic conditions, government support, and other development activities may have influenced the observed differences. The manuscript also states that the observed differences cannot be attributed solely to the intervention. This part of the response is appropriate.

However, the response is not completely consistent with the revised manuscript. Reviewer #1 specifically requested that the findings be interpreted as associations rather than causal effects. Despite the authors’ statement that they have removed causal language, causal expressions remain in the Abstract, Results, and Conclusion.

Example still present in the manuscript: “The substantial improvements were likely driven by structured IGA implementation, with asset transfers, direct cash support, and nutrition education.”
Recommended replacement: “The observed improvements coincided with implementation of the structured IGA program, although the contribution of other temporal and contextual factors cannot be excluded.”

Another example is the Results wording: “The results showed a significant association over the time period due to the intervention.” This still attributes the observed association to the intervention.
Recommended replacement: “The results showed a significant difference in outcome prevalence between the 2022 and 2024 survey rounds after propensity score weighting.”

**Response:** Thank you for your careful assessment and for highlighting the remaining inconsistencies between our responses and the revised manuscript. We agree with the highlighted points. In response to your comment, we carefully reviewed the Abstract, Results, Discussion, and Conclusion sections again and removed and sometimes revised the remaining causal language. Specifically, we replaced causal terms (e.g., “effect,” “impact,” “led to,” “resulted in,” “improved,” and similar wording where applicable) with more appropriate observational terms such as “association,” “relationship,” “difference,” “correlation,” and “was observed to be associated with.” We have also revised the interpretation in the Discussion and Conclusion sections to ensure that the findings are presented as associations, rather than causal estimates.

Your suggestion was to change the first line of the conclusion; however, we revised the entire conclusion to better align with our findings. Please see page 3, lines 48-53.

Similarly, the Results wording: “The results showed a significant association over the time period due to the intervention” has been revised to: “The results showed a significant difference in outcome prevalence between the 2022 and 2024 survey rounds after propensity score weighting.” Please see page-21, line-396-397.

Also please see page-2, line-27; page-2, line-35; page-3, line-48-53; page-6-7, line-125-126; page-7, line-127; page-13, line-279; page-15, line-288,296; page-20, line-381; page-21, line 401-405, page-22, line-418-419; page-27, line-505.

**Comment 2: Study Design and Limitations**

Assessment: Adequately addressed. The revised limitations section is substantially stronger than the previous version. It acknowledges that the analysis was based on two cross-sectional survey rounds and that propensity score weighting cannot eliminate bias from unmeasured factors. It also discusses differences in survey timing, short recall periods, seasonal variation, food prices, dietary patterns, climatic shocks, economic conditions, government support, and other development factors.

The manuscript appropriately states that future research should establish causality and acknowledges that the study did not fully adhere to a rigorous impact evaluation design. This directly responds to Reviewer #1’s concern.

Recommended wording to retain or strengthen: “Because the study was based on two repeated cross-sectional survey rounds without random assignment or a concurrent control group, residual confounding and other time-varying contextual factors cannot be excluded. Therefore, the observed changes should be interpreted as associations rather than definitive causal effects of the intervention.”

**Response:** Thank you so much for your feedback. As per your suggestion, we have added the recommended wording to strengthen the discussion section. Please see page 26, lines 487-490.

**Comment 3: Propensity Score Weighting and Covariate Balance**

Assessment: Mostly addressed, but additional reporting would improve the response. The authors added a standardized mean difference/Love plot showing covariate balance before and after inverse probability weighting. This is an appropriate response to Reviewer #1’s request.

However, the manuscript currently states that “most covariates” moved closer to zero and remained within acceptable limits. If the intended criterion is an absolute standardized mean difference below 0.10, the manuscript should explicitly state this and report whether all covariates met this threshold after weighting.

Recommended wording, if supported by the actual data: “After inverse probability weighting, all absolute standardized mean differences were below 0.10, indicating adequate balance across the measured covariates.”

If one or more covariates remain above 0.10, the authors should report this transparently rather than stating that all covariates were adequately balanced.

**Response:** Thank you for this suggestion. We have updated the text for Fig 4, which shows that SMDs are within the limit of 0.10 for all covariates. Please see page-20, lines 376-379.

**Comment 4: Propensity Score Overlap and Positivity**

Assessment: Partially addressed or insufficiently documented. The manuscript presents propensity-score distributions and describes them as showing good overlap. However, the reported distributions differ substantially across some propensity-score categories. This does not necessarily invalidate the analysis, but the authors should provide a clearer assessment of common support and weighting diagnostics.

The authors should consider reporting whether extreme propensity scores or extreme weights were present, whether the weights were stabilized or truncated, and whether the effective sample size changed substantially after weighting. These details would make the inverse probability weighting analysis more transparent and reproducible.

Recommended wording: “The propensity-score distributions showed overlap across the observed range, although their distributions differed between survey rounds. Weight diagnostics were examined to assess the influence of extreme propensity scores and weights.”

**Response:** Thank you. We have updated the text accordingly and mentioned the positivity assumption; please see page-20, lines 383-387.

**Comment 5: Absolute Effects Alongside Relative Effects**

Assessment: Adequately addressed statistically, but terminology should be corrected. The authors added absolute effect estimates using the weighted binomial model. For household food security, the weighted estimate corresponds to a 0.16 increase in probability, or a 16-percentage-point difference. For dietary diversity, the corresponding estimate is 0.53, or a 53-percentage-point difference. This appropriately complements the relative risk estimates.
The remaining problem is terminology. The manuscript labels these estimates as “average intervention effects.” Given the observational repeated cross-sectional design, this terminology can imply a causal treatment effect.

Recommended replacement for the Results/Table heading: “Weighted differences in outcome prevalence between 2022 and 2024.”

Recommended wording: “The weighted binomial model estimated a 0.16 higher probability of household food security in 2024 compared with 2022 (95% CI: 0.09–0.22), corresponding to a 16-percentage-point difference.”

**Response:** We have updated the title of the table and text accordingly; please see page 19, line 373.

**Comment 6: Overall Consistency Between the Response Letter and Manuscript**

Assessment: This requires correction before acceptance. The response to Reviewer #1 states that the authors carefully revised the manuscript to remove residual causal language. However, the revised manuscript still contains terms such as “impact,” “intervention effects,” “average intervention effects,” “due to the intervention,” “likely driven by,” and “effective in improving.”

This creates a discrepancy between the response letter and the actual manuscript. The reviewer may reasonably identify this discrepancy in another review round. The authors should therefore perform a final search of the entire manuscript and response letter for causal terminology and revise every occurrence consistently.

Recommended Manuscript-Wide Replacements

• “impact of the intervention” → “changes associated with program implementation”

• “intervention effects” → “weighted associations” or “differences between survey rounds”
• “average intervention effect” → “weighted difference in outcome prevalence”

• “due to the intervention” → “coinciding with program implementation”

• “likely driven by the intervention” → “may have coincided with program implementation”

• “interventions are effective in improving” → “intervention implementation was positively associated with”

• “caused by” → “associated with” or “observed alongside”

**Suggested Revised Conclusion**

“In this repeated cross-sectional study, household food security and dietary diversity were substantially higher in 2024 than in 2022 among vulnerable women in the study areas. These differences remained after propensity score weighting, indicating a positive association between program implementation and the study outcomes. However, because the study was non-randomized and based on two cross-sectional survey rounds, residual confounding and other time-varying contextual factors cannot be excluded. Therefore, the observed differences should not be interpreted as definitive causal effects of the intervention. Future controlled or longitudinal evaluations are needed to establish the causal effectiveness and long-term sustainability of integrated nutrition-sensitive interventions.”

**Response:** Thank you for your valuable suggestion. We have revised the entire manuscript and deleted or revised the terms. Please see the revised terms in page-2, line-27; page-2, line-35; page-3, line-48-53; page-6-7, line-125-126; page-7, line-127; page-13, line-279; page-15, line-288,296; page-20, line-381; page-21, line 401-405, page-22, line-418-419; page-27, line-505. We have also revised the conclusion as per your suggestion; please see page-3, lines- 48-53, page-27-28, lines-522-530.

**Reviewers 2**

Reviewer #2: (No Response)

**Reviewers 3**

**Reviewer #3:** Thank you for the opportunity to review the revised manuscript and for the authors’ efforts to address my previous comments. However, I believe that the concern regarding the statistical interpretation of Figure 2 remains unresolved.

Figure 2 is a descriptive chart presenting changes in household food security status and dietary diversity between 2022 and 2024. The figure itself does not present the results of a statistical analysis, yet the Results section (Page 17, lines 335–340) reports statistically significant changes with corresponding p-values. The authors should therefore distinguish between the descriptive percentages presented in Figure 2 and the inferential analysis used to obtain the reported p-values. Simply stating in the figure note that these differences are statistically significant does not address the underlying issue of how and from which statistical analysis the reported p-values were obtained. [1.1]

I recommend that the authors explicitly state which statistical method or model generated these p-values and clarify whether they were derived from the propensity-score-weighted analysis or from a separate statistical test. The figure can remain as a presentation of unweighted sample proportions, but its note should clearly indicate that the reported p-values are derived from the specified statistical analysis. This would somehow resolve the ambiguity regarding the source and interpretation of the reported statistical significance.

The authors may also consider presenting these results in a table rather than a figure, as a table would allow the 2022 and 2024 proportions, magnitude of change, and corresponding statistical results to be presented together. However, regardless of whether a figure or table is retained, the statistical method used to generate the reported p-values should be explicitly stated.

**Response:** We have excluded the text that includes unadjusted p-values. We have shown the adjusted p-values in the later tables. We believe that these are no longer required. Please see page-17, lines-337-340.
