# Supplementary material for: Integrated nutrition-sensitive interventions are associated with improvements in household food security and dietary diversity among vulnerable women in the host communities of Cox’s Bazar, Bangladesh: Findings from a pre-post comparison study
Source: PLoS One. 2026 Nov 5;21(11):e0362124. doi: 10.1371/journal.pone.0362124 (PMC13739155; doi:10.1371/journal.pone.0362124)
Supplement: Peer review history

# Peer review: Decision Letter 0

**DOI:** 10.1371/journal.pone.0362124.r001

**Authors:** Keiko Nakamura

11 Mar 2026

Dear Dr. Mridha,

Thank you for submitting your manuscript to PLOS ONE. After careful consideration, we feel that it has merit but does not fully meet PLOS ONE’s publication criteria as it currently stands. Therefore, we invite you to submit a revised version of the manuscript that addresses the points raised during the review process.

Please read the comments from the reviewers carefully and amend the manuscript accordingly.

Please submit your revised manuscript by Apr 25 2026 11:59PM. If you will need more time than this to complete your revisions, please reply to this message or contact the journal office at plosone@plos.org. When you're ready to submit your revision, log on to https://www.editorialmanager.com/pone/ and select the 'Submissions Needing Revision' folder to locate your manuscript file.

- A letter that responds to each point raised by the academic editor and reviewer(s). You should upload this letter as a separate file labeled 'Response to Reviewers'.
- A marked-up copy of your manuscript that highlights changes made to the original version. You should upload this as a separate file labeled 'Revised Manuscript with Track Changes'.
- An unmarked version of your revised paper without tracked changes. You should upload this as a separate file labeled 'Manuscript'.

If you would like to make changes to your financial disclosure, please include your updated statement in your cover letter. Guidelines for resubmitting your figure files are available below the reviewer comments at the end of this letter.

If applicable, we recommend that you deposit your laboratory protocols in protocols.io to enhance the reproducibility of your results. Protocols.io assigns your protocol its own identifier (DOI) so that it can be cited independently in the future. For instructions see: https://journals.plos.org/plosone/s/submission-guidelines#loc-laboratory-protocols. Additionally, PLOS ONE offers an option for publishing peer-reviewed Lab Protocol articles, which describe protocols hosted on protocols.io. Read more information on sharing protocols at https://plos.org/protocols?utm_medium=editorial-email&utm_source=authorletters&utm_campaign=protocols.

We look forward to receiving your revised manuscript.

Kind regards,

Keiko Nakamura

Academic Editor

PLOS One

Journal Requirements:

When submitting your revision, we need you to address these additional requirements.

1. Please ensure that your manuscript meets PLOS ONE's style requirements, including those for file naming. The PLOS ONE style templates can be found at

https://journals.plos.org/plosone/s/file?id=wjVg/PLOSOne_formatting_sample_main_body.pdf and

https://journals.plos.org/plosone/s/file?id=ba62/PLOSOne_formatting_sample_title_authors_affiliations.pdf

2. Thank you for stating the following financial disclosure:

"MKM received the fund. This study was funded by UNICEF (Contract No: 43349187; Date: 2 May 2022. The funders were involved with

the conceptualization of the study design, data supervision, and reviewing the

manuscript."

Please state what role the funders took in the study.  If the funders had no role, please state: "The funders had no role in study design, data collection and analysis, decision to publish, or preparation of the manuscript."

If this statement is not correct you must amend it as needed.

Please include this amended Role of Funder statement in your cover letter; we will change the online submission form on your behalf.

3.Your ethics statement should only appear in the Methods section of your manuscript. If your ethics statement is written in any section besides the Methods, please move it to the Methods section and delete it from any other section. Please ensure that your ethics statement is included in your manuscript, as the ethics statement entered into the online submission form will not be published alongside your manuscript.

4. If the reviewer comments include a recommendation to cite specific previously published works, please review and evaluate these publications to determine whether they are relevant and should be cited. There is no requirement to cite these works unless the editor has indicated otherwise.

[Note: HTML markup is below. Please do not edit.]

Reviewers' comments:

Reviewer's Responses to Questions

**Comments to the Author**

1. Is the manuscript technically sound, and do the data support the conclusions?

Reviewer #1: Partly

Reviewer #2: Partly

Reviewer #3: Partly

Reviewer #4: Partly

**********

2. Has the statistical analysis been performed appropriately and rigorously?

Reviewer #1: Yes

Reviewer #2: N/A

Reviewer #3: Yes

Reviewer #4: Yes

**********

3. Have the authors made all data underlying the findings in their manuscript fully available?

The PLOS Data policy

Reviewer #1: No

Reviewer #2: Yes

Reviewer #3: Yes

Reviewer #4: Yes

**********

4. Is the manuscript presented in an intelligible fashion and written in standard English?

Reviewer #1: Yes

Reviewer #2: Yes

Reviewer #3: Yes

Reviewer #4: Yes

**********

Reviewer #1: Overall assessment

This manuscript responds to an important applied question for humanitarian and fragile contexts by asking whether a multisectoral package of nutrition-sensitive income-generating activities (either backyard poultry rearing or tailoring), cash transfers, and nutrition education was associated with improved household food security and women’s dietary diversity in vulnerable women-headed households in Cox’s Bazar. The subject matter is within the scope of PLOS ONE because it is policy-relevant and likely generalizable to similar contexts. The manuscript is overall well-motivated, intervention description is concrete, and the use of multiple analytical approaches (standard regression, inverse probability weighting, and propensity score regression) represents a strength.

But the way this is currently designed and framed raises huge concerns about causal interpretation. The analysis compares 2 repeated cross-sectional (2022 vs 2024) samples of program beneficiaries and uses survey year as a proxy for exposure to the intervention. With no contemporaneous comparison group or clearly specified baseline pre-exposure to the program, results should be viewed as changes in time amongst those exposed to the program rather than representing an ‘impact’ estimate. Another domain requiring correction to ensure interpretability and reproducibility is several reporting and coding issues (the direction of binary outcome coding, terminology around matching vs weighting). Finally, addressing the main points below would substantially improve your manuscript.

Major comments

1) Specify the evaluation design, estimand and causal language

The paper repeatedly refers to an ‘impact’ evaluation, but the design is closer to a pre–post (or early–late) comparison among beneficiaries. Both samples were selected from the same list of 2,000 beneficiaries and initiation of the intervention in 2021 makes it difficult to say that the sample for 2022 is a clear baseline pre-intervention. Estimate: (a) expose clearly the exposure variable (e.g., months since receiving assets/cash/training); (b) specify the estimand (average treatment effect among beneficiaries, change in prevalence over time, and so on); and (c) amend claims of causality accordingly; should not use ‘causes,’ say that is ‘associated with’ or ‘turns over time ± unless stronger identification were made. If authors want to make a stronger causal inference, then I would recommend introducing an analytic cohort for comparison (non-beneficiaries from similar unions or sub-districts) or consider using quasi-experimental designs—such as difference-in-differences if pre-intervention baseline data is available or interrupted time series with multiple pre/post points).

2) Discrepancies between interpretations and outcome definitions/coding

The text construes binary outcomes in a reversed way when it comes to the interpretation of odds ratios. For HFIAS, the manuscript says households were recategorized into 1 = moderately/severely food insecure and 0 = food secure/mildly food insecure but then interprets OR>1 meaning more likely to be food secure. Similarly, here, dietary diversity is defined as 1 = insufficient DD (<5 food groups), however OR>1 interpreted that there is better DD. Thank you for being sensitive to the labels, which should match the modeled outcome. And since the ORs are very central to what the conclusions are, this is a major correctness issue.

3) Lack of methodological reporting of propensity scores and inconsistent use of terminology

Abstract and Methods refer to propensity score matching, but Tables and text report weighted data (after assigning weight’) and later also report PS regression. Exactly what was done: (i) PS model specification (which variables, functional forms, interactions, etc.), (ii) matching if used (algorithm/caliper/replacement/matching ratio), or weighting if used (stabilized weights/trimming), diagnosis for pre/post-matching imbalance between groups/standardized mean differences for all covariates with cutoff threshold/plots, and how uncertainty was computed under weighting-robust/HC standard errors. Whenever matching is not done, change ‘matching’ to ‘weighting’ everywhere. Also, whether the estimand is marginal (ATE/ATT) and what has to do with the three models that are reported.

4) Temporal confounding, secular shifts and seasonality are significant threats to validity

Seasonality in food access and diet could account for some of the observed differences, because timing of survey differed (first round Aug 2022–Mar 2023; second round Mar–Jun 2024) and outcomes depend on recent recall periods (HFIAS past 4 weeks; MDD-W past 24h). And, of 2022–2024, macroeconomic shocks and price changes cause shifts in food security regardless of the program. Please take care to include month/season controls (or a sensitivity restricting to comparable months), contextual data (e.g., local food price index if you have it, as relevant for your data in context), and discussion that sets explicit boundaries around what the study can/not attribute to the intervention.

5) Consider the exposure and ‘dose’ of the intervention rather than using survey year as a proxy

The intervention package includes multiple components (training, assets, cash transfers in six installments, supervision, nutrition education). If the program rollout was not uniform, beneficiaries in 2022 may have had different intensity/duration of exposure than those in 2024. If data exist, incorporate direct measures: time since training/asset receipt, number of follow-up visits attended, cash installments received, participation in nutrition education, and program fidelity indicators. Even a simple stratified analysis by exposure duration would strengthen interpretation and align better with the ‘process evaluation’s framing

6) Consider effect measures more interpretable than odds ratios for common outcomes

Dietary diversity adequacy appears to reach ~86% in 2024; with common outcomes, odds ratios can exaggerate relative differences. Consider reporting risk ratios or prevalence ratios using log-binomial or modified Poisson models with robust SEs, alongside absolute differences in prevalence. PLOS ONE readers often value absolute changes (percentage-point differences) for policy translation.

Minor comments and technical edits

• Figures seem mislabeled: the figure that is labeled Fig 1 in the PDF appendix appears to be food-group consumption, while the figure labeled Fig 2 seems to show food security categories. Their figure files and captions should match the in-text citations, with clear panel labels if Fig 1 is meant to reflect both food security and dietary diversity.

• Clarify coding for marital status: Table 2 reports very low percentages ~9–13% for ‘married’. It may be so due to women-headed household eligibility, but the readers will challenge it. Explain briefly in Methods or Results.

• In Table 1 and main text, streamline terminology: ‘food security’ or at least use ‘food insecurity’; ‘adequate’ vs ‘inadequate’ dietary diversity. Table 1. Coding should follow the example manner of hitting the narrative.

• Some Discussion statements are too strong for the design (e.g., ‘strong empirical evidence’ and ‘it can be claimed’). Detone provides to even emphasize a basic association and talk about residual confounding.

• Reword the sentence saying that propensity score methods are ‘better than’ logistic regression. Under certain conditions PS methods may reduce model dependence, but they are not panacea.

• If possible, report exact p-values; but goals guide readers to effect sizes and CIs. Do not overemphasize statistical significance alone.

• More intervention implementation details relevant to generalizability: who delivered nutrition education, frequency, curriculum and whether participation was mandatory/optional.

• Include STROBE checklist (observational designs) and TIDieR checklist [29] elements for intervention reporting.

• Minor language/typo checking: ‘background poultry’ must be ‘backyard poultry’; consistent spelling of Cox’s Bazar; unit/exchange rate verification (using an August 2025 converter for a 2021–2024 programme may baffle the audience).

• Confirm whether individuals could be sampled in each round. If yes, adjust for possible repeated measures or clustering; if not, describe how duplicates were prevented

Reviewer #2: The manuscript addresses an important issue and presents encouraging findings from a highly relevant setting. It has clear practical significance, and the intervention package appears meaningful for vulnerable women in Cox’s Bazar. However, the manuscript would benefit from several improvements, as outlined in the following specific comments:

1. The background would benefit from more context from studies conducted in similar settings as Cox’s Bazar or comparable humanitarian or low-resource settings. In particular, it would strengthen the introduction to discuss what is already known about nutrition-sensitive interventions such as tailoring, poultry rearing, or other income-generating activities, including their sustainability and their relevance to this setting.

2. The interpretation of findings should be more cautious and avoid strong causal wording. The study compares two independently sampled cross-sectional groups from 2022 and 2024, and the time variable was used as a proxy for intervention exposure. This design supports an association over time, but not strong causal attribution. Therefore, statements such as “The substantial improvements were likely driven by structured IGA implementation, with asset transfers, direct cash support, and nutrition education” in the abstract may need to be softened and replaced with wording that stays closer to the observed findings. This applied to the discussion and conclusion as well.

3. Please elaborate more clearly on how the propensity score approach was used. The manuscript uses terms such as propensity score matching, weighting, IPW, and PS regression, but these are not presented consistently across sections. It would be helpful to use consistent terminology throughout and explain more clearly why this approach is appropriate when comparing survey rounds rather than exposed versus unexposed groups.

4. Since all participants were beneficiaries and there is no non-beneficiary control group, it is difficult to separate intervention-related change from secular trends, recovery over time, or other contextual influences. This should be discussed more explicitly as a major limitation of the study design.

5. Table 2 shows the covariate distribution before and after weighting. The presentation would be stronger if the authors reported standardized mean differences more explicitly for all variables.

6. The first round of the survey was conducted from August 2022 to March 2023, while the second round was conducted from March to June 2024. Please discuss the different timing of the two survey rounds and whether seasonal variation may have influenced food security and dietary diversity outcomes.

7. Some parts of the discussion go beyond what was directly measured. For example, the discussion refers to nutrition knowledge (line 420) or empowerment (line 428-428), but it is not clear whether these variables were included in the final analysis. If such variables were captured in the questionnaire, the authors may consider incorporating them into the analysis or at least clarifying this point. Including findings on knowledge-related measures, if available, could make the discussion more convincing.

8. It may also be useful to consider whether women’s living arrangement or household decision-making context could influence food selection and dietary diversity. If such information was collected, the authors may want to explore whether it should be included among the explanatory variables.

9. The title is somewhat long and could be made more concise.

10. Since the manuscript includes comparative outcome analysis, the term “process evaluation study” in the title may not fully reflect the analysis presented. The authors may consider terms such as “repeated cross-sectional evaluation” or “program evaluation” instead.

11. Please elaborate more clearly on the rationale for the dietary diversity classification, including why the chosen cut-off was used and how it was validated or supported by other literature.

12. Similarly, please clarify if the Household Food Insecurity Access Scale (HFIAS) has been validated in Bangladesh context.

13. Current figures and tables would benefit from clearer presentation. Please indicate whether the percentages shown are weighted or unweighted, and ensure that each figure and table can be understood without requiring extensive reference back to the main text.

Reviewer #3: General Comment

This manuscript addresses an important topic, assessing the impact of integrated nutrition-sensitive interventions on household food security and dietary diversity among vulnerable women in Cox’s Bazar, Bangladesh. The study has the potential to provide valuable insights. However, there are several methodological and reporting issues that require clarification to enhance transparency, reproducibility, and consistency throughout the manuscript.

Specific Comments

1. Abstract (Lines 19–20):

The phrase “underlying causes of nutrition” appears to be incorrect. It would be more accurate to state “underlying causes of malnutrition” to convey the intended meaning.

2. Sample Size Calculation (Line 192):

The statement “The sample size was calculated using the standard formula” is vague. The authors should specify the formula used and clarify what is meant by the “prevalence of 0.5.” Is this referring to the prevalence of food insecurity or inadequate dietary diversity or something else? A reference supporting this prevalence estimate should also be provided. Moreover, there are two outcome variables with two different prevalences, which one was used and why?

3. Sampling Description (Lines 207–208):

The phrase “randomization was conducted independently for each round” is misleading, as this is not a randomized trial. It is likely that the authors meant that participants were randomly selected independently for each round. Clarification is needed.

4. Sampling Procedure:

The manuscript states that participants were “randomly selected,” but the procedure is not described in detail. The authors should clarify:

• Whether simple random sampling or stratified sampling was used.

• Whether sampling was stratified by intervention type (backyard poultry vs. tailoring).

5. Re-interviewed Participants (Second Round, 2024):

The authors should report the proportion of participants in the second round who were re-interviewed from the first round. This information is important to clarify the independence of the two survey rounds in this repeated cross-sectional study.

6. Outcome Variable Coding and Consistency:

There is inconsistency in reporting and coding of the outcome variable:

• In Line 220, the primary outcome is described as “household food insecurity.”

• In Table 1, it is presented as “household food security,” coded as 1 = moderate to severe food insecurity and 0 = food secure to mildly food insecure.

• Line 263 indicates that the “food insecurity category” was treated as the reference category.

For example, in binary logistic regression, the reference category is typically coded as 0, while the event category is coded as 1. Clarification is needed on which category was treated as the reference in the regression analysis, and whether the interpretation of odds ratios aligns with the coding. The authors are encouraged to harmonize the terminology, coding scheme, and regression interpretation to ensure methodological clarity and consistency.

7. Propensity Scores:

The manuscript would benefit from a brief explanation of propensity scores in the Methods section, including what they are and how they should be interpreted in the context of the analysis. This will help readers better understand their role in the study.

8. Figures 1 & 2:

It appears that Figures 1 and 2 may have been inadvertently interchanged. Figure 1 does not show the changes in household food security as indicated in the footnote (Line 307), suggesting a possible mix-up. The authors should review the figures and ensure that each figure and its legend accurately correspond to the data presented.

9. Accuracy of Data Presentation:

The authors should ensure that all statements referring to figures or tables accurately reflect the data presented. For example, Lines 301–303 state:

“A significant improvement in food security status was observed among vulnerable women between 2022 and 2024 (Fig 1). The prevalence of household food security among vulnerable women improved substantially, from 9% in 2022 to 34% (p<0.001) in 2024.”

However, Figure 1 does not display p-values, which suggests that these may have been derived from a table that is not referenced here. The authors should clarify the source of the statistical results and ensure that the text accurately points to the corresponding figure or table throughout the Results section.

10. Conclusion: Statements in the conclusion that are not directly supported by the study findings should be avoided. For example, the statement: “Sustained investment, strong political commitment, and intersectoral collaboration are essential for scaling and institutionalizing such approaches within national development frameworks” does not appear to be directly derived from the results presented in this study. Conclusions should be grounded in the study’s empirical findings. In scientific writing, conclusions that extend beyond the data may be considered speculative and can weaken the overall rigor of the manuscript. The authors are encouraged to revise the conclusion to ensure it aligns closely with the evidence generated by the study.

Reviewer #4: Thank you for giving me the opportunity to review an interesting paper. The study provides valuable evidence on an important humanitarian issue. With a clear revision to address the confusion between "process evaluation" and "impact evaluation," as well as a clarification of the limitations regarding time-varying confounders, this manuscript would be a strong contribution to literature. I recommend Minor Revision.

1. Consistency with the title and studied outcomes: The manuscript describes an impact evaluation (measuring food security and dietary diversity outcomes using propensity score matching) while its title states "process evaluation study”. A process evaluation typically focuses on implementation fidelity, reach, participant experience, and barriers to delivery. The current manuscript provides little on the process of the intervention (e.g., implementation challenges, participant attendance, and fidelity to training modules). I recommend either updating the title and scope to "Impact Evaluation" or significantly expanding the manuscript to include actual process evaluation metrics.

2. Logic model presentation: Given that the authors claim this is a process evaluation, the lack of a visual logic model is a significant gap. A logic model would clarify the pathway from intervention (backyard poultry/tailoring/cash) to intermediate outcomes (household income) to final outcomes (food security/dietary diversity). I strongly suggest the authors include a conceptual framework or logic model diagram to illustrate how the integrated interventions were hypothesized to impact outcomes.

3. Intervention groups: The authors acknowledge they could not independently examine the effects of backyard poultry rearing vs. tailoring due to sample size, so they combined them. This aggregation assumes that both interventions operate through similar mechanisms to produce similar magnitudes of effect. The authors should provide brief justification for why these are considered sufficiently similar to combine or discuss this as a key limitation.

4. Confounding: The study uses two cross-sectional rounds (2022 and 2024). While propensity score matching addresses baseline differences between the populations sampled, it does not fully control for external, time-varying confounders (e.g., changes in the local economy, food prices, or other NGO activities in the area between 2022 and 2024). The authors should acknowledge this in the discussion as a potential threat to the attribution of causality to the specific intervention.

<minor comments="">

1. Terminology (Page 11, line 228) : In the definition of food insecurity, you state "1 for moderately to severely food insecure, and 0 for food secure to mildly food insecure." Please clarify the rationale for grouping "mildly food insecure" with "food secure".

2. Authors note that 5% of interviews were re-interviewed. It would be helpful to briefly state the results of these quality checks—were there high discrepancies.

3. Table 1. Type of IGA assistance" is listed as an explanatory variable, yet the study mentions combining them due to sample size. Please clarify if this was used in the propensity score calculation or the final model.

1. Consistency with the title and studied outcomes: The manuscript describes an impact evaluation (measuring food security and dietary diversity outcomes using propensity score matching) while its title states "process evaluation study”. A process evaluation typically focuses on implementation fidelity, reach, participant experience, and barriers to delivery. The current manuscript provides little on the process of the intervention (e.g., implementation challenges, participant attendance, and fidelity to training modules). I recommend either updating the title and scope to "Impact Evaluation" or significantly expanding the manuscript to include actual process evaluation metrics.

2. Logic model presentation: Given that the authors claim this is a process evaluation, the lack of a visual logic model is a significant gap. A logic model would clarify the pathway from intervention (backyard poultry/tailoring/cash) to intermediate outcomes (household income) to final outcomes (food security/dietary diversity). I strongly suggest the authors include a conceptual framework or logic model diagram to illustrate how the integrated interventions were hypothesized to impact outcomes.

3. Intervention groups: The authors acknowledge they could not independently examine the effects of backyard poultry rearing vs. tailoring due to sample size, so they combined them. This aggregation assumes that both interventions operate through similar mechanisms to produce similar magnitudes of effect. The authors should provide brief justification for why these are considered sufficiently similar to combine or discuss this as a key limitation.

4. Confounding: The study uses two cross-sectional rounds (2022 and 2024). While propensity score matching addresses baseline differences between the populations sampled, it does not fully control for external, time-varying confounders (e.g., changes in the local economy, food prices, or other NGO activities in the area between 2022 and 2024). The authors should acknowledge this in the discussion as a potential threat to the attribution of causality to the specific intervention.

<minor comments="">

1. Terminology (Page 11, line 228) : In the definition of food insecurity, you state "1 for moderately to severely food insecure, and 0 for food secure to mildly food insecure." Please clarify the rationale for grouping "mildly food insecure" with "food secure".

2. Authors note that 5% of interviews were re-interviewed. It would be helpful to briefly state the results of these quality checks—were there high discrepancies.

3. Table 1. Type of IGA assistance" is listed as an explanatory variable, yet the study mentions combining them due to sample size. Please clarify if this was used in the propensity score calculation or the final model.</minor></minor>

**********

what does this mean?). If published, this will include your full peer review and any attached files.

If you choose “no”, your identity will remain anonymous but your review may still be made public.

**Do you want your identity to be public for this peer review?**  For information about this choice, including consent withdrawal, please see our Privacy Policy

Reviewer #1: No

Reviewer #2: No

Reviewer #3: **Yes:**  Dr. Anderson Bendera

Reviewer #4: No

**********

[NOTE: If reviewer comments were submitted as an attachment file, they will be attached to this email and accessible via the submission site. Please log into your account, locate the manuscript record, and check for the action link "View Attachments". If this link does not appear, there are no attachment files.]

To ensure your figures meet our technical requirements, please review our figure guidelines: https://journals.plos.org/plosone/s/figures

You may also use PLOS’s free figure tool, NAAS, to help you prepare publication quality figures: https://journals.plos.org/plosone/s/figures#loc-tools-for-figure-preparation.

NAAS will assess whether your figures meet our technical requirements by comparing each figure against our figure specifications.

# Author comment: Author response to Decision Letter 1

**DOI:** 10.1371/journal.pone.0362124.r002

23 May 2026

Manuscript ID: PONE-D-26-04366

Manuscript title: Integrated nutrition-sensitive interventions are associated with improvements in household food security and dietary diversity among vulnerable women in the host communities of Cox’s Bazar, Bangladesh: Findings from a pre-post comparison study

Reviewers 1

Reviewer #1: Overall assessment

This manuscript responds to an important applied question for humanitarian and fragile contexts by asking whether a multisectoral package of nutrition-sensitive income-generating activities (either backyard poultry rearing or tailoring), cash transfers, and nutrition education was associated with improved household food security and women’s dietary diversity in vulnerable women-headed households in Cox’s Bazar. The subject matter is within the scope of PLOS ONE because it is policy-relevant and likely generalizable to similar contexts. The manuscript is overall well-motivated, intervention description is concrete, and the use of multiple analytical approaches (standard regression, inverse probability weighting, and propensity score regression) represents a strength.

But the way this is currently designed and framed raises huge concerns about causal interpretation. The analysis compares 2 repeated cross-sectional (2022 vs 2024) samples of program beneficiaries and uses survey year as a proxy for exposure to the intervention. With no contemporaneous comparison group or clearly specified baseline pre-exposure to the program, results should be viewed as changes in time amongst those exposed to the program rather than representing an ‘impact’ estimate. Another domain requiring correction to ensure interpretability and reproducibility is several reporting and coding issues (the direction of binary outcome coding, terminology around matching vs weighting). Finally, addressing the main points below would substantially improve your manuscript.

Response: Thank you for your thoughtful and constructive assessment. We appreciate your recognition of the study’s policy relevance, the clear intervention description, and the use of multiple analytical approaches. We agree with your concerns regarding causal interpretation and reporting clarity, and we have revised the manuscript to frame the findings more cautiously as changes over time among exposed households, clarified the analytic terminology and coding, and addressed the specific issues raised.

Major comments

1) Specify the evaluation design, estimand and causal language

The paper repeatedly refers to an ‘impact’ evaluation, but the design is closer to a pre–post (or early–late) comparison among beneficiaries. Both samples were selected from the same list of 2,000 beneficiaries and initiation of the intervention in 2021 makes it difficult to say that the sample for 2022 is a clear baseline pre-intervention. Estimate: (a) expose clearly the exposure variable (e.g., months since receiving assets/cash/training); (b) specify the estimand (average treatment effect among beneficiaries, change in prevalence over time, and so on); and (c) amend claims of causality accordingly; should not use ‘causes,’ say that is ‘associated with’ or ‘turns over time ± unless stronger identification were made. If authors want to make a stronger causal inference, then I would recommend introducing an analytic cohort for comparison (non-beneficiaries from similar unions or sub-districts) or consider using quasi-experimental designs—such as difference-in-differences if pre-intervention baseline data is available or interrupted time series with multiple pre/post points).

Response: Thank you for your valuable comment. We agree that the original wording overstated the strength of our study design. We have revised the manuscript to describe the study as a pre-post comparison study among beneficiaries rather than an impact evaluation. Please see the title in page 1. We have also removed causal language throughout the manuscript and replaced terms such as “causes” and “impact” with more appropriate language, such as “associated with,” “changes observed over time,” and “differences between survey rounds.” It has been modified in the overall results section. We agree that the exposure variables should be stated more clearly. In our study, all beneficiaries received the same type of program support, including cash/assets/training; however, the support was provided in different months and years according to Upazila, which means that in the same Upazila, all beneficiaries received support on the same day. The sub-district/study area variable has already been added in the propensity score analysis; therefore, the duration of exposure is already adjusted. Regarding the estimand, we now state more explicitly that our analysis estimates changes in outcome prevalence and associated factors between the two survey rounds among programme beneficiaries, rather than an average treatment effect or a causal programme effect. Please see the method section page 9, lines 183-186; page 10, lines 213-220 and page 15, lines 295-307.

2) Discrepancies between interpretations and outcome definitions/coding

The text construes binary outcomes in a reversed way when it comes to the interpretation of odds ratios. For HFIAS, the manuscript says households were recategorized into 1 = moderately/severely food insecure and 0 = food secure/mildly food insecure but then interprets OR>1 meaning more likely to be food secure. Similarly, here, dietary diversity is defined as 1 = insufficient DD (<5 food groups), however OR>1 interpreted that there is better DD. Thank you for being sensitive to the labels, which should match the modeled outcome. And since the ORs are very central to what the conclusions are, this is a major correctness issue.

Response: Thank you for pointing this out. We sincerely apologize for this mistake. We have corrected the outcome variables. For outcome variables, we explored household food security and women’s dietary diversity (DD). For the HFIAS, we categorized it into two groups: 0 = moderately/severely food insecure as food insecure and 1 = food secure, which includes food secure/mildly food insecure. This binary categorization has been followed by other studies and supports other valid documents; references have been added to the manuscript. Please see the revised lines with the correct references on page 12, line 256-262. In addition, we defined dietary diversity as 0 = inadequate DD (<5 food groups) and 1 = adequate DD (≥5 food groups). See page 13, lines 270-273.

3) Lack of methodological reporting of propensity scores and inconsistent use of terminology

Abstract and Methods refer to propensity score matching, but Tables and text report weighted data (after assigning weight’) and later also report PS regression. Exactly what was done: (i) PS model specification (which variables, functional forms, interactions, etc.), (ii) matching if used (algorithm/caliper/replacement/matching ratio), or weighting if used (stabilized weights/trimming), diagnosis for pre/post-matching imbalance between groups/standardized mean differences for all covariates with cutoff threshold/plots, and how uncertainty was computed under weighting-robust/HC standard errors. Whenever matching is not done, change ‘matching’ to ‘weighting’ everywhere. Also, whether the estimand is marginal (ATE/ATT) and what has to do with the three models that are reported.

Response: Thank you for your valuable comment. We have revised the Abstract, Methods, Tables, and Results sections to ensure consistent terminology and clearer methodological reporting. Specifically, we stated that our analysis used inverse probability weighting (IPW) based on propensity scores rather than propensity score matching. No matching procedures were performed.

We clarified the propensity score model specification in the methods section by identifying the covariates included in the treatment model: age, marital status, education level, occupation, household income, type of IGA assistance, wealth quintile, and sub-district/study area. We also specify how these variables were entered in the model, for example, categorical/continuous.

We further clarify that the target estimand is the marginal average treatment effect (ATE), and that this was estimated on the risk ratio scale using a modified Poisson regression model with stabilized inverse probability weights. Please see page 2, lines 33-39; page 15-17, lines 291-347.

4) Temporal confounding, secular shifts and seasonality are significant threats to validity

Seasonality in food access and diet could account for some of the observed differences, because timing of survey differed (first round Aug 2022–Mar 2023; second round Mar–Jun 2024) and outcomes depend on recent recall periods (HFIAS past 4 weeks; MDD-W past 24h). And From 2022 to 2024, macroeconomic shocks and price changes cause shifts in food security regardless of the program. Please take care to include month/season controls (or a sensitivity restricting to comparable months), contextual data (e.g., local food price index if you have it, as relevant for your data in context), and discussion that sets explicit boundaries around what the study can or cannot attribute to the intervention.

Response: Thank you for your valuable comment. We agree that temporal confounding, seasonal variations in food access and diet, and broader macroeconomic changes between survey rounds may influence the observed differences. Unfortunately, our dataset does not contain direct measures of month-specific seasonality, local food price indices or other contextual market indicators. To address this limitation, we have revised the manuscript in two ways as follows. First, we softened the causal language and reframed the findings as associations observed across two survey rounds rather than effects attributable solely to the intervention. Second, we have added a clear limitation in the Methods and Discussion sections, acknowledging that differences in timing, recall windows, seasonal food access, prices, and economic conditions may have contributed to changes in the HFIAS and MDD-W outcomes. Please see the revised and newly added lines on page 15, lines 299-302, and page 27-28, lines 528-538.

5) Consider the exposure and ‘dose’ of the intervention rather than using survey year as a proxy

The intervention package includes multiple components (training, assets, cash transfers in six installments, supervision, nutrition education). If the program rollout was not uniform, beneficiaries in 2022 may have had different intensity/duration of exposure than those in 2024. If data exist, incorporate direct measures: time since training/asset receipt, number of follow-up visits attended, cash installments received, participation in nutrition education, and program fidelity indicators. Even a simple stratified analysis by exposure duration would strengthen interpretation and align better with the ‘process evaluation’s framing

Response: Thank you for this important comment. In our study, all beneficiaries received the same type of program support, including cash/assets/training; however, the support was provided in different months and years according to Upazila, which means that in the same Upazila, all beneficiaries received support on the same day. The sub-district/study area variable is already added in the propensity score analysis, so here, the duration of exposure is already adjusted. However, we did not assess participation intensity in nutrition education sessions and, therefore, could not examine variations in engagement with that component. Consequently, we were unable to conduct dose-response or stratified analyses based on exposure duration. We have revised the manuscript to frame the analysis in terms of the intervention effect rather than the survey year, and we have added this as a limitation in the discussion section.

6) Consider effect measures more interpretable than odds ratios for common outcomes

Dietary diversity adequacy appears to reach ~86% in 2024; with common outcomes, odds ratios can exaggerate relative differences. Consider reporting risk ratios or prevalence ratios using log-binomial or modified Poisson models with robust SEs, alongside absolute differences in prevalence. PLOS ONE readers often value absolute changes (percentage-point differences) for policy translation.

Response: Thank you for your suggestion. Accordingly, we revised the analysis. The intervention effect was estimated using modified Poisson regression and inverse probability of sampling weighting (IPSW). Table 4 has been revised to reflect these updated analyses (page 23-24). We have also revised the corresponding results section; please see pages 22 to 23, lines 421-441.

Minor comments and technical edits

• Figures seem mislabeled: the figure that is labeled Fig 1 in the PDF appendix appears to be food-group consumption, while the figure labeled Fig 2 seems to show food security categories. Their figure files and captions should match the in-text citations, with clear panel labels if Fig 1 is meant to reflect both food security and dietary diversity.

Response: Thank you for pointing this out. The labels in Figures 1 and 2 have been mistakenly interchanged. We have corrected this error in the revised manuscript. We apologize for this oversight. In the new version, we have three figures. The previous 1 no figure will be figure 3, and 2 no figure will be figure 2.

• Clarify coding for marital status: Table 2 reports very low percentages ~9–13% for ‘married’. It may be so due to women-headed household eligibility, but the readers will challenge it. Explain briefly in Methods or Results.

Response: Thank you for your valuable comment. The low proportion of married participants reflects the intervention eligibility criteria, which primarily targeted economically vulnerable women from households with no stable source of income or those relying on irregular or casual labor employment. Consequently, the study population was drawn from a highly specific vulnerable group, which helps explain the marital status distribution shown in Table 2. We have added a brief clarification in the methods and results section to assist readers in interpreting these findings. We have mentioned the eligibility criteria in the methods section (please see page 7, lines 143-149).

• In Table 1 and main text, streamline terminology: ‘food security’ or at least use ‘food insecurity’; ‘adequate’ vs ‘inadequate’ dietary diversity. Table 1. Coding should follow the example manner of hitting the narrative.

Response: Thank you for your helpful suggestion. We have revised the terminology in Table 1 and throughout the main text to ensure consistency with the text. For outcome variables, we explored household food security and women’s dietary diversity (DD). For the HFIAS, we categorized it into two groups: 0 = moderately/severely food insecure as food insecure and 1 = food secure, which includes food secure/mildly food insecure. This binary categorization has been followed by other studies and supports other valid documents; references have been added to the manuscript. Please see the revised lines with the correct references on page 12, line 256-262. In addition, we defined dietary diversity as 0 = inadequate DD (<5 food groups) and 1 = adequate DD (≥5 food groups). See page 13, lines 270-273.

• Some Discussion statements are too strong for the design (e.g., ‘strong empirical evidence’ and ‘it can be claimed’). Detone provides to even emphasize a basic association and talk about residual confounding.

Response: Thank you for this helpful observation. We agree that some statements in the Discussion section were stronger than those warranted by the study design. We have revised the manuscript to use more cautious language throughout and avoid overstating the findings. Specifically, terms such as ‘strong empirical evidence’ and ‘it can be claimed’ have been replaced with wording that emphasizes observed associations only. Please see page 26, lines 506-509, and page 29, line 561.

• Reword the sentence saying that propensity score methods are ‘better than’ logistic regression. Under certain conditions PS methods may reduce model dependence, but they are not panacea.

Response: Thank you for this important comment. We agree that the earlier wording was too strong and could have been misleading. In the revised Discussion section, we removed the statement suggesting that propensity score methods are better than logistic regression. Instead, we now describe propensity score weighting more cautiously as an alternative analytic approach that can help improve the balance in observed covariates and reduce model dependence under certain assumptions, but not as a universally superior method. This revision is consistent with the updated Methods section, where we clarified that our analysis used inverse propensity score weighting with modified Poisson regression for outcome estimation. Please see page-26, lines 519-523.

• If possible, report exact p-values; but goals guide readers to effect sizes and CIs. Do not overemphasize statistical significance alone.

Response: Thank you for your helpful suggestion. We agree that effect sizes and confidence intervals should be emphasized over statistical significance. In the revised manuscript, we added the standard errors for the estimated risk ratios to improve the transparency of the results. We have also revised the presentation of the findings to focus more clearly on the magnitude and precision of the estimated associations rather than relying only on statistical significance.

• More intervention implementation details relevant to generalizability: who delivered nutrition education, frequency, curriculum and whether participation was mandatory/optional.

Response: Thank you for your helpful suggestion. We agree that additional details on intervention implementation are important for assessing generalizability. We have now clarified the key implementation characteristics of the nutrition education component, including who delivered the sessions, the frequency of delivery, the curriculum content, and the nature of participation. A detailed description of these elements is provided in Supplementary Table S1, which expands on the intervention description presented in the main text. We have also added a brief summary in the Methods (Intervention description) section and referred readers to Supplementary Table S1 for complete details.

• Include STROBE checklist (observational designs) and TIDieR checklist [29] elements for intervention reporting.

Response: Thank you for suggesting. We have included this checklist in supplementary files (S3 and S4 files).

• Minor language/typo checking: ‘background poultry’ must be ‘backyard poultry’; consistent spelling of Cox’s Bazar; unit/exchange rate verification (using an August 2025 converter for a 2021–2024 programme may baffle the audience).

Response: Thank you for your suggestion. We have corrected the typo “background poultry”; please see page 25, line 475 of the revised manuscript. We have also made the spelling of Cox’s Bazar consistent throughout the manuscript, including the correction in page 7, line 136. Finally, we retained the USD conversion rate as of January 1, 2021. Please see the revisions on page 7, lines 145-147, and page 9, line 183.

• Confirm whether individuals could be sampled in each round. If yes, adjust for possible repeated measures or clustering; if not, describe how duplicates were prevented

Response: Thank you for this important comment. The two survey rounds were based on independent samples, and the study was not designed to follow the same individual over time. Participants in each round were selected separately according to the sampling framework. Therefore, repeated measurements at the individual level were not expected, and adjustments for within-person correlations were not applicable. We have clarified this in the methods section of the revised manuscript. Please see page-11, lines 229-230.

Reviewers 2

Reviewer #2: The manuscript addresses an important issue and presents encouraging findings from a highly relevant setting. It has clear practical significance, and the intervention package appears meaningful for vulnerable women in Cox’s Bazar. However, the manuscript would benefit from several improvements, as outlined in the following specific comments:

1. The background would benefit from more context from studies conducted in similar settings as Cox’s Bazar or comparable humanitarian or low-resource settings. In particular, it would strengthen the introduction to discuss what is already known about nutrition-sensitive interventions such as tailoring, poultry rearing, or other income-generating activities, including their sustainability and their relevance to this setting.

Response: Thank you for your suggestion. We have already included a discussion in the introduction on nutrition-sensitive interventions in similar humanitarian and low-resource settings, including income-generating activities such as poultry rearing and tailoring, as well as their relevance to Cox’s Bazar. We have carefully checked this section and clarified the wording to make the context more explicit. Please see page: 4-6 line: 71-85, 92-95, 103-112.

2. The interpretation of the findings should be more cautious and avoid strong causal wording. The study compares two independently sampled cross-sectional groups from 2022 and 2024, and the time variable was used as a proxy for intervention exposure. This design supports an association over time, but not strong causal attribution. Therefore, statements such as “The substantial improvements were likely driven by structured IGA implementation, with asset transfers, direct cash support, and nutrition education” in the abstract may need to be softened and replaced with wording that stays closer to the observed findings. This applied to the discussion and conclusion as well.

Response: Thank you for this important comment. We agree that the interpretation of our findings should be more cautious and should not imply strong causal attribution, given the repeated cross-sectional design and the use of time as a proxy for intervention exposure. In the revised manuscript, we have softened the language in the abstract, discussion, and conclusion sections and removed statements that could be interpreted as causal.

Specifically, we now state: “Our study provides evidence of an association between nutrition-sensitive interventions coupled with nutrition education and improved household food security and dietary diversity among vulnerable women in low-income Bangladesh, although residual confounding cannot be excluded.” This revised wording more accurately reflects the observational nature of the study and the limitations of causal inference. Please see page 29, lines- 561-564.

3. Please elaborate more clearly on how the propensity score approach was used. The manuscript uses terms such as propensity score matching, weighting, IPW, and PS regression, but these are not presented consistently across sections. It would be helpful to use consistent terminology throughout and explain more clearly why this approach is appropriate when comparing survey rounds rather than exposed versus unexposed groups.

Response: Thank you for this important comment. We have revised the Abstract, Methods, Tables, and Results to ensure consistent terminology and clearer methodological reporting. Specifically, we now state throughout that our analysis used inverse probability weighting (IPW) based on propensity scores, rather than propensity score matching. No matching procedure was performed.

We clarified the propensity score model specification in the methods section by identifying the covariates included in the treatment model: age, marital status, education level, occupation, household income, type of IGA assistance, wealth quintile, and sub-district/study area. We also specify how these variables were entered in the model, for example, categorical/continuous. Please see page 15-17, lines 308-347.

4. Since all participants were beneficiaries and there is no non-beneficiary control group, it is difficult to separate intervention-related change from secular trends, recovery over time, or other contextual influences. This should be discussed more explicitly as a major limitation of the study design.

Response: Thank you for your suggestion. We have added this to the limitations in the discussion section (page 27, lines 528-538.

5. Table 2 shows the covariate distribution before and after weighting. The presentation would be stronger if the authors reported standardized mean differences more explicitly for all variables.

Response: Thank you for this valuable suggestion. We agree that standardized mean differences for all covariates would strengthen the assessment of balance before and after the weighting. However, these diagnostics were not included in the revised manuscript. We acknowledge this as a limitation and note that Table 2 presents the covariate distributions before and after weighting as a descriptive indication of sample balance.

6. The first round of the survey was conducted from August 2022 to March 2023, while the second round was conducted from March to June 2024. Please discuss the different timing of the two survey rounds and whether seasonal variation may have influenced food security and dietary diversity outcomes.

Response: Thank you for this important comment. We agree that the different timing of the two survey rounds may have influenced household food security and dietary diversity because of possible seasonal variations in food availability, income, expenditure patterns, and consumption practices. In the revised manuscript, we have clarified the timing of both survey rounds in the Methods section and added a note in the discussion/limitations section to acknowledge that the surveys were not conducted in the same calendar period. This difference occurred because of operational constraints, including administrative permission delays and a fixed project timeline, which required the completion of data collection within the implementation period. We now explicitly acknowledge that seasonal differences may have affected the observed outcomes and that this should be considered when interpreting the changes between the two survey rounds. Please see page-15, lines 299-302 and page- 26-27, lines 528-538.

7. Some parts of the discussion go beyond what was directly measured. For example, the discussion refers to nutrition knowledge (line 420) or empowerment (line 428-428), but it is not clear whether these variables were included in the final analysis. If such variables were captured in the questionnaire, the authors may consider incorporating them into the analysis or at least clarifying this point. Including findings on knowledge-related measures, if available, could make the discussion more convincing.

Response: Thank you for pointing this out. In the Discussion section, we noted that alongside improvements in food security and dietary diversity, women’s empowerment and nutrition knowledge may also have improved. In the second survey round, 93.35% of women reported improved decision-making power regarding the purchase of food and daily household essentials. However, we did not formally examine these variables in the present study. In addition, decision-making power could not be included in the model because of its highly uneven distribution, which affected model stability and the final estimates. To avoid confusion and overinterpretation, we have removed this paragraph from the revised manuscript. Please see the revised discussion section, page-25, lines 491-502.

8. It may also be useful to consider whether women’s living arrangement or household decision-making context could influence food selection and dietary diversity. If such information was collected, the authors may want to explore whether it should be included among the explanatory variables.

Response: Thank you for your helpful suggestion. We considered the household economic position in the analysis through the wealth quintile variable, which partly reflects differences in household living conditions. Therefore, living arrangement-related variation was addressed indirectly in the model and was not presented as a separate explanatory variable.

Information was collected regarding the household decision-making context. However, in the 2024 survey round, a very high proportion of women (93.35%) reported that they made decisions regarding food purchases and daily household essentials, spending earned money, etc. Owing to this highly uneven distribution, the variable provided very limited variability and was not suitable for inclusion in the adjusted model.

9. The title is somewhat long and could be made more concise.

Response: Thank you for this suggestion. We agree that the original title was somewhat lengthy. The title has been revised for clarity. Please see the revised title on page 1.

10. Since the manuscript includes comparative outcome analysis, the term “process evaluation study” in the title may not fully reflect the analysis presented. The authors may consider terms such as “repeated cross-sectional evaluation” or “program evaluation” instead.

Response: Thank you for your helpful suggestion. We agree that the term “process evaluation study” does not fully reflect the comparative outcome analysis presented in the manuscript. In line with the reviewer’s suggestion and to better represent the study design, we have revised the title to: “Integrated nutrition-sensitive interventions are associated with improvements in household food security and dietary diversity among vulnerable women in the host communities of Cox’s Bazar, Bangladesh: Findings from a pre-post comparison study.” We believe that this revised title more accurately reflects the analytical approach and scope of the study. Please see the revised title on page 1.

11. Please elaborate more clearly on the rationale for the dietary diversity classification, including why the chosen cut-off was used and how it was validated or supported by other literature.

Response: Thank you for your helpful comments. The rationale for the dietary diversity classification was already described in the Methods section using the Minimum Dietary Diversity for Women of Reproductive Age (MDD-W) framework with supporting references. In the revised manuscript, we have further clarified that the cutoff of ≥5 out of 10 food groups is the standard MDD-W threshold used to indicate adequate dietary diversity and better micronutrient adequacy among women of reproductive age, while consumption from <5 food groups was classified as inadequate dietary diversity. Please see page-12-13, lines 263-273.

12. Similarly, please clarify if the Household Food Insecurity Access Scale (HFIAS) has been validated in Bangladesh context.

Response: HFIAS is a standard scale used in the Food Security and Nutrition Surveillance Project in Bangladesh. Although this binary classification has not been specifically validated in the Bangladeshi context, it is widely used internationally to categorize household food insecurity. We have now included the relevant references in the manuscript; please see page 12, lines 256-262.

13. Current figures and tables would benefit from clearer presentation. Please indicate whether the percentages shown are weighted or unweighted, and ensure that each figure and table can be understood without requiring extensive reference back to the main text.

Response: Thank you for your helpful suggestion. We confirm that all percentages presented in the tables and figures are not weighted. We have now revised the table and figure legends to explicitly state this. In addition, we have improved the clarity of all tables and figures by adding clearer titles, labels, and footnotes so that each can be interpreted independently, without requiring reference to the main text.

Reviewers 3

Reviewer #3: General Comment

This manuscript addresses an important topic, assessing the impact of integrated nutrition-sensitive interventions on household food security and dietary diversity among vulnerable women in Cox’s Bazar, Bangladesh. This study has the potential to provide valuable insights. However, there are several methodological and reporting issues that require clarification to enhance transparency, reproducibility, and consistency throughout the manuscript.

Specific Comments

1. Abstract (Lines 19–20):

The phrase “underlying causes of nutrition” appears to be incorrect. It would be more accurate to state “underlying causes of malnutrition” to convey the intended meaning.

Response: Thank you for your suggestion; it has been revised. please see page-2, line-21.

2. Sample Size Calculation (Line 192):

The statement “The sample size was calculated using the standard formula” is vague. The authors should specify the formula used and clarify what is meant by the “prevalence of 0.5.” Is this referring to the prevalence of food insecurity or inadequate dietary diversity or something else? A reference supporting this prevalence estimate should also be provided. Moreover, there are two outcome variables with two different prevalences, which one was used and why?

Response: Thank you for this important comment. We agree that the previous description of the sample size calculation was unclear. We have now revised the Methods section to specify that the sample size was calculated using the single-population proportion formula, n = Z2 p(1−p)/d2. The assumed prevalence of 50% (p = 0.5) was not intended to represent the observed prevalence of a specific study outcome; rather, it was used as a conservative assumption because no reliable prior estimate was available for the study population. This approach was chosen because a prevalence of 50% yielded the maximum sample size. We have clarified this in the manuscript and added relevant references. Please see page 10, lines 200-206. Thank you for your suggestion. We included two outcome variables because they reflect two related but distinct dimensions of nutritional well-being. Household food insecurity captures household-level access to food, whereas women’s dietary diversity reflects the quality and variety of food consumed at the individual level. Both outcomes are important for understanding the broader nutrition situation among vulnerable women and assessing the potential association of the intervention with household food access and women’s diet quality.

3. Sampling Description (Lines 207–208):

The phrase “randomization was conducted independently for each round” is misleading, as this is not a randomized trial. It is likely that the authors meant that participants were randomly selected independently for each round. Clarification is needed.

Response: Thank you for observing this important point. We have revised the line to indicate that the participants were randomly selected (page-10, line 218-220).

4. Sampling Procedure:

The manuscript states that participants were “randomly selected,” but the procedure is not described in detail. The authors should clarify:

• Whether simple random sampling or stratified sampling was used.

• Whether sampling was stratified by intervention type (backyard poultry vs. tailoring).

Response: Thank you for your comment. Participants were selected using simple random sampling method. The sampling was not stratified by intervention type (e.g., backyard poultry rearing or tailoring). We have clarified this in the Methods section to provide a more detailed description of the sampling procedures. Please see page 10, lines 214-215.

5. Re-interviewed Participants (Second Round, 2024):

The authors should report the proportion of participants in the second round who were re-interviewed from the first round. This information is important for clarifying the independence of the two survey rounds in this repeated cross-sectional study.

Response: Thank you for this important comment. The surveys were designed as independent cross-sectional rounds, and participants were selected separately in each round according to the sampling framework. Unique identifiers were not collected to link individuals across rounds; therefore, it was not possible to determine whether any participants in the second round also participated in the first round. Consequently, the analysis treated the two samples as independent samples. We have clarified this in the Methods section. Please see page-10, lines 218-220.

6. Outcome Variable Coding and Consistency:

There is inconsistency in reporting and coding of the outcome variable:

• In Line 220, the primary outcome is described as “household food insecurity.”

• In Table 1, it is presented as “household food security,” coded as 1 = moderate to severe food insecurity and 0 = food secure to mildly food insecure.

• Line 263 indicates that the “food insecurity category” was treated as the reference category.

For example, in binary logistic regression, the reference category is typically coded as 0, while the event category is coded as 1. Clarification is needed on which category was treated as the reference in the regression analysis, and whether the interpretation of odds ratios aligns with the coding. The authors are encouraged to harmonize the terminology, coding scheme, and regression interpretation to ensure methodological clarity and consistency.

Response: Thank you for pointing this issue, we apologies for the mistake. We have corrected the outcome variables. For outcome variables, we explored household food security and women’s dietary diversity (DD). For the HFIAS, we categorized it into two groups: 0 = moderately/severely food insecure as food insecure and 1 = food secure, which includes food secure/mildly food insecure. This binary categorization has been followed by other studies and supports other valid documents; references have been added to the manuscript. Please see the revised lines with the correct references on page 12, line 256-262.

7. Propensity Scores:

The manuscript would benefit from a brief explanation of propensity scores in the Methods section, including what they are and how they should be interpreted in the context of the analysis. This will help readers better understand their role in the study.

Response: Thank you for your comments. The method section is revised, please see page 15-17.

8. Figures 1 & 2:

It appears that Figures 1 and 2 may have been inadvertently interchanged. Figure 1 does not show the changes in household food security as indicated in the footnote (Line 307), suggesting a possible mix-up. The authors should review the figures and ensure that each figure and its legend accurately correspond to the data presented.

Response: Thank you for pointing this out. The labels in Figures 1 and 2 have been mistakenly interchanged. We have corrected this error in the revised manuscript. We apologize for the oversight.

9. Accuracy of Data Presentation:

The authors should ensure that all statements referring to figures or tables accurately reflect the data presented. For example, Lines 301–303 state:

“A significant improvement in food security status was observed among vulnerable women between 2022 and 2024 (Fig 1). The prevalence of household food security among vulnerable women improved substantially, from 9% in 2022 to 34% (p<0.001) in 2024.”

However, Figure 1 does not display p-values, which suggests that these may have been derived from a table that is not referenced here. The authors should clarify the source of the statistical results and ensure that the text accurately points to the corresponding figure or table throughout the Results section.

Response: Thank you for pointing this out. The labels in Figures 1 and 2 have been mistakenly interchanged. We have now corrected this error in the revised manuscript. We apologize for the oversight.

10. Conclusion: Statements in the conclusion that are not directly supported by the study findings should be avoided. For example, the statement: “Sustained investment, strong political commitment, and intersectoral collaboration are essential for scaling and institutionalizing such approaches within national development frameworks” does not appear to be directly derived from the results presented in this study. Conclusions should be grounded in the study’s empirical findings. In scientific writing, conclusions that extend beyond the data may be considered speculative and can weaken the overall rigor of the manuscript. The authors are encouraged to revise the conclusion to ensure it aligns closely with the evidence generated by the study.

Response: Thank you for this important comment. We agree that the statement on sustained investment, political commitment, and intersectoral collaboration extends beyond the empirical findings of the present study. In response, we have revised the conclusion to remove this broader policy-oriented statement and ensure that the concluding remarks are more closely aligned with the evidence generated by the study. Please see page-29, lines 569-572.

Reviewers 4

Reviewer #4: Thank you for giving me the opportunity to review an interesting paper. The study provides valuable evidence on an important humanitarian issue. With a clear revision to address the confusion between "process evaluation" and "impact evaluation," as well as a clarification of the limitations regarding time-varying confounders, this manuscript would be a strong contribution to literature. I recommend Minor Revision.

Comment 1. Consistency with the title and studied outcomes: The manuscript describes an impact evaluation (measuring food security and dietary diversity outcomes using propensity score matching) while its title states "process evaluation study”. A process evaluation typically focuses on implementation fidelity, reach, participant experience, and barriers to delivery. The current manuscript provides little on the process of the intervention (e.g., implementation challenges, participant attendance, and fidelity to training modules). I recommend either updating the title and scope to "Impact Evaluation" or significantly expanding the manuscript to include actual process evaluation metrics.

Response: Thank you for this important comment. We agree that the term “process evaluation study” did not accurately reflect the primary focus of our analysis, which is a comparative assessment of outcomes. In response, we have revised the title to better align with the study design and analytical approach: “Integrated nutrition-sensitive interventions are associated with improvements in household food security and dietary diversity among vulnerable women in the host communities of Cox’s Bazar, Bangladesh: Findings from a pre-post comparison study.”

We also reviewed the manuscript to ensure consistency between the title, objectives, and reported outcomes. The revised title removes the implication of a process evaluation and more appropriately reflects the outcome-focused analysis conducted using propensity score methods. Please see the updated title on page 1.

Comment 2. Logic model presentation: Given that the authors claim this is a process evaluation, the lack of a visual logic model is a significant gap. A logic model would clarify the pathway from intervention (backyard poultry/tailoring/cash) to intermediate outcomes (household income) to final outcomes (food security/dietary diversity). I strongly suggest the authors include a conceptual framework or logic model diagram to illustrate how the integrated interventions were hypothesized to impact outcomes.

Response: Thank you for this valuable suggestion. We agree that a logic model helps clarify the hypothesized pathways linking interventions to outcomes. We have now included a conceptual framework (Fig 1) in the Methods section to illustrate the pathway from the integrated interventions (backyard poultry, tailoring, and cash support) to intermediate outcomes (e.g., household income and knowledge) and final outcomes (food security and dietary diversity). This addition improves the clarity of the process-evaluation framework.

3. Intervention groups: The authors acknowledge they could not independently examine the effects of backyard poultry rearing vs. tailoring due to sample size, so they combined them. This aggregation assumes that both interventions operate through similar mechanisms to produce similar magnitudes of effect. The authors should provide brief justification for why these are considered sufficiently similar to combine or discuss this as a key limitation.

Response: Thank you for this important comment. We agree that combining backyard poultry rearing and tailoring into a single intervention category requires further justification. These two intervention arms were combined because both were implemented within the same overall program framework and designed to support women’s livelihoods through small, home-based income-generating activities. In both groups, participants received resources to initiate or strengthen home-based livelihoods, financial support for their activities, and education on nutrition and hygiene practices. The main difference between the two groups was the specific type of income-generating activity: poultry rearing or tailoring. Given these shared program components and the limited sample size within the individual categories, we considered it reasonable to combine them for the analysis. We have now clarified this rationale in the manuscript’s method section and noted the inability to assess their independent effects as a study limitation.

4. Confounding: The study uses two cross-sectional rounds (2022 and 2024). While propensity score matching addresses baseline differences between the populations sampled, it does not fully control for external, time-varying confounders (e.g., changes in the local economy, food prices, or other NGO activities in the area between 2022 and 2024). The authors should acknowledge this in the discussion as a potential threat to the attribution of causality to the specific intervention.

Response: Thank you for this important comment. We agree that although propensity score matching improves comparability between the intervention and comparison groups based on observed covariates, it cannot fully account for unmeasured or time-varying contextual factors operating between the 2022 and 2024 survey rounds. Changes in the local economy, food prices, weather conditions, government programs, or the activities of other NGOs may have also influenced household food security during the study period. We have now acknowledged this more explicitly in the discussion (page 27-28, lines 528-538).

1. Terminology (Page 11, line 228): In the definition of food insecurity, you state "1 for moderately to severely food insecure, and 0 for food secure to mildly food insecure." Please clarify the rationale for grouping "mildly food insecure" with "food secure".

Response: Thank you for your comment. We agree that the rationale for grouping mildly food-insecure households with food-secure households must be clarified. Following prior applications of this multidimensional classification approach, we dichotomized the outcome into food secure/mildly food insecure versus moderately/severely food insecure to create a simpler, policy-relevant contrast for estimating treatment effects. This recategorization was intended to distinguish households experiencing more substantial deprivation from those with no or relatively limited food insecurity. We have revised the manuscript accordingly. Please see page 12, lines 255-262.

2. Authors note that 5% of interviews were re-interviewed. It would be helpful to briefly state the results of these quality checks—were there high discrepancies.

Response: Thank you for your helpful comments. As part of the data quality assurance process, re-interviews were conducted only when discrepancies exceeded 25%. In our quality checks, the level of discrepancy was below this threshold and did not exceed 12%. These discrepancies were reviewed and corrected through discussions with the research assistants and respondents within 48 hours of data collection. We have now clarified this in the manuscript; please see page 11-12, lines 237-243.

3. Table 1. Type of IGA assistance" is listed as an explanatory variable, yet the study mentions combining them due to sample size. Please clarify if this was used in the propensity score calculation or the final model.

Response: Thank you for your comment. Due to the small sample size in the individual IGA categories, the type of IGA assistance variable was combined into broader categories before the analysis. The combined variable was then included in the models. We have clarified this in the methods section. Moreover, we have clarified this in the Methods section (page-13, lines 278-283).

Attachment Submitted filename: Response to Reviewers.docx

# Peer review: Decision Letter 1

**DOI:** 10.1371/journal.pone.0362124.r003

**Authors:** Keiko Nakamura

21 Jun 2026

Dear Dr. Mridha,

==============================

publication criteriaplosone@plos.org. When you're ready to submit your revision, log on to https://www.editorialmanager.com/pone/ and select the 'Submissions Needing Revision' folder to locate your manuscript file.

- A letter that responds to each point raised by the academic editor and reviewer(s). You should upload this letter as a separate file labeled 'Response to Reviewers'.
- A marked-up copy of your manuscript that highlights changes made to the original version. You should upload this as a separate file labeled 'Revised Manuscript with Track Changes'.
- An unmarked version of your revised paper without tracked changes. You should upload this as a separate file labeled 'Manuscript'.

If you would like to make changes to your financial disclosure, please include your updated statement in your cover letter. Guidelines for resubmitting your figure files are available below the reviewer comments at the end of this letter.

If applicable, we recommend that you deposit your laboratory protocols in protocols.io to enhance the reproducibility of your results. Protocols.io assigns your protocol its own identifier (DOI) so that it can be cited independently in the future. For instructions see: https://journals.plos.org/plosone/s/submission-guidelines#loc-laboratory-protocols. Additionally, PLOS ONE offers an option for publishing peer-reviewed Lab Protocol articles, which describe protocols hosted on protocols.io. Read more information on sharing protocols at https://plos.org/protocols?utm_medium=editorial-email&utm_source=authorletters&utm_campaign=protocols.

As the corresponding author, your ORCID iD is verified in the submission system and will appear in the published article. PLOS supports the use of ORCID, and we encourage all coauthors to register for an ORCID iD and use it as well. Please encourage your coauthors to verify their ORCID iD within the submission system before final acceptance, as unverified ORCID iDs will not appear in the published article. *Only*  the individual author can complete the verification step; PLOS staff *cannot*  verify ORCID iDs on behalf of authors.

We look forward to receiving your revised manuscript.

Kind regards,

Keiko Nakamura

Academic Editor

PLOS One

**Journal Requirements:**

1. If the reviewer comments include a recommendation to cite specific previously published works, please review and evaluate these publications to determine whether they are relevant and should be cited. There is no requirement to cite these works unless the editor has indicated otherwise.

2. Please review your reference list to ensure that it is complete and correct. If you have cited papers that have been retracted, please include the rationale for doing so in the manuscript text, or remove these references and replace them with relevant current references. Any changes to the reference list should be mentioned in the rebuttal letter that accompanies your revised manuscript. If you need to cite a retracted article, indicate the article’s retracted status in the References list and also include a citation and full reference for the retraction notice.

[Note: HTML markup is below. Please do not edit.]

Reviewers' comments:

Reviewer's Responses to Questions

**Comments to the Author**

Reviewer #1: (No Response)

Reviewer #2: (No Response)

Reviewer #3: (No Response)

**********

2. Is the manuscript technically sound, and do the data support the conclusions?

Reviewer #1: (No Response)

Reviewer #2: Yes

Reviewer #3: Yes

**********

3. Has the statistical analysis been performed appropriately and rigorously?

Reviewer #1: (No Response)

Reviewer #2: Yes

Reviewer #3: Yes

**********

4. Have the authors made all data underlying the findings in their manuscript fully available?

The PLOS Data policy

Reviewer #1: (No Response)

Reviewer #2: Yes

Reviewer #3: Yes

**********

5. Is the manuscript presented in an intelligible fashion and written in standard English?

Reviewer #1: (No Response)

Reviewer #2: No

Reviewer #3: Yes

**********

**Reviewer #1:**  Overall Assessment

This revised manuscript evaluates the association between integrated nutrition-sensitive interventions and improvements in household food security and dietary diversity among vulnerable women in Cox’s Bazar, Bangladesh using repeated cross-sectional survey data and propensity score-based weighting methods.

The study addresses a highly relevant humanitarian and public health issue. The intervention is policy-relevant, and the dataset is valuable in a fragile and resource-constrained setting. The authors have made substantial improvements in response to prior comments, particularly in clarifying the study design, correcting methodological inconsistencies, and improving reporting transparency.

However, important methodological and inferential limitations remain that affect the strength of interpretation, particularly regarding causal claims, confounding control, and design limitations.

Major Strengths

1. Policy-relevant topic with strong humanitarian importance.

2. Improved methodological clarity (PS weighting, IPW, corrected terminology).

3. Appropriate use of modified Poisson regression for risk ratios.

4. Large sample size and real-world program data improving external validity.

Major Concerns

1. Causal inference remains overstated despite revisions. The design is repeated cross-sectional with no control group.

2. Residual confounding remains due to unmeasured and time-varying factors (seasonality, macroeconomic changes, NGO activities).

3. Exposure definition using survey year remains conceptually weak.

4. Lack of full standardized mean difference (SMD) balance diagnostics reduces transparency.

5. Effect size interpretation may be optimistic; absolute differences should be emphasized more.

Minor Concerns

1. Some sections still contain overly generalized interpretations.

2. Figures and tables improved but could be more self-explanatory.

3. Intervention fidelity details remain limited.

Overall Interpretation

The authors have significantly improved the manuscript. However, the study design limits causal inference, and findings should be interpreted as associations rather than effects.

The manuscript is suitable for publication after minor revision, provided interpretation is further moderated.

Recommendation

Minor Revision

Key requirements:

- Remove residual causal language

- Strengthen discussion of limitations

- Add balance diagnostics clarity if possible

- Emphasize absolute effects alongside risk ratios

**Reviewer #2:**  I thank the authors for the detailed response to the previous comments. Many points have been addressed satisfactorily. However, several of the original concerns remain unresolved, and I have identified a few additional issues that require attention before the manuscript is suitable for publication.

1. The authors stated they had softened causal language throughout the manuscript. However, in the submitted revision this language does not appear consistently, e.g

+ The Results section (lines 404 and 411) attributes findings to the intervention with the phrases "due to the intervention."

Such passages must be revised to reflect the observational, associational nature of the study.

2. Similarly, line 338 states: "The prevalence of household food security among vulnerable women got relatively better, from 9% in 2022 to 34% in 2024." The phrase "got relatively better" is casual and does not meet academic writing standards. Please revise.

3. The response stated that the paragraph mentioning women's empowerment had been removed from the Discussion. However, lines 491 to 499 about the intervention remains: "…contributed to enhanced women's empowerment" and "likely facilitated their greater involvement in household decision-making processes". As noted previously, this goes beyond the empirical findings.

4. The abstract (line 41–42) reports RR=3.29 (95% CI: 2.88–3.77) for dietary diversity. However, RR=3.29 is the unweighted modified Poisson regression estimate. The primary result, and the one correctly reported for food security in the same abstract is the IPSW (weighted) estimate. For dietary diversity, the IPSW result is RR=2.78. Reporting the unweighted estimate in the abstract as the headline finding is inconsistent with the study's stated analytic approach.

5. References 16 and 30 cite URLs beginning with "chrome-extension://...". Readers and editors cannot follow these links. The authors must replace these with the actual publicly available URLs, DOIs, or complete archival source information.

**Reviewer #3:**  Thank you for the opportunity to review the revised manuscript. The authors have addressed most of my comments satisfactorily. However, I believe that Comment 9 regarding the accuracy of data presentation has not been fully addressed.

In this particular comment, my main concern was not related to the numbering or labeling of Figures 1 and 2, but rather to the inconsistency between the narrative text and the data presented in the figures. The manuscript reports a statistically significant improvement in household food security (p<0.001) and refers readers to a figure 2 as supporting evidence. However, the figure itself does not present p-values or statistical test results. As a result, it remains unclear where the reported p-value originates and whether it is derived from a table or a separate statistical analysis.

While the authors indicate that the figure labels were mistakenly interchanged and have now been corrected, this does not resolve the underlying issue. I recommend that the authors explicitly identify the source of the statistical results and ensure that all references to figures and tables accurately correspond to the data being presented throughout the Results section.

Therefore, I consider this comment to be only partially addressed and would appreciate further clarification from the authors before a final decision is made.

**********

what does this mean?). If published, this will include your full peer review and any attached files.

If you choose “no”, your identity will remain anonymous but your review may still be made public.

**Do you want your identity to be public for this peer review?**  For information about this choice, including consent withdrawal, please see our Privacy Policy

Reviewer #1: No

Reviewer #2: No

Reviewer #3: No

**********

[NOTE: If reviewer comments were submitted as an attachment file, they will be attached to this email and accessible via the submission site. Please log into your account, locate the manuscript record, and check for the action link "View Attachments". If this link does not appear, there are no attachment files.]

To ensure your figures meet our technical requirements, please review our figure guidelines: https://journals.plos.org/plosone/s/figures

You may also use PLOS’s free figure tool, NAAS, to help you prepare publication quality figures: https://journals.plos.org/plosone/s/figures#loc-tools-for-figure-preparation.

NAAS will assess whether your figures meet our technical requirements by comparing each figure against our figure specifications.

# Author comment: Author response to Decision Letter 2

**DOI:** 10.1371/journal.pone.0362124.r004

30 Jun 2026

Manuscript ID: PONE-D-26-04366R1

Manuscript title: Integrated nutrition-sensitive interventions are associated with improvements in household food security and dietary diversity among vulnerable women in the host communities of Cox’s Bazar, Bangladesh: Findings from a pre-post comparison study

Reviewers 1

Reviewer #1: Overall Assessment

This revised manuscript evaluates the association between integrated nutrition-sensitive interventions and improvements in household food security and dietary diversity among vulnerable women in Cox’s Bazar, Bangladesh using repeated cross-sectional survey data and propensity score-based weighting methods.

The study addresses a highly relevant humanitarian and public health issue. The intervention is policy-relevant, and the dataset is valuable in a fragile and resource-constrained setting. The authors have made substantial improvements in response to prior comments, particularly in clarifying the study design, correcting methodological inconsistencies, and improving reporting transparency.

However, important methodological and inferential limitations remain that affect the strength of interpretation, particularly regarding causal claims, confounding control, and design limitations.

Major Strengths

1. Policy-relevant topic with strong humanitarian importance.

2. Improved methodological clarity (PS weighting, IPW, corrected terminology).

3. Appropriate use of modified Poisson regression for risk ratios.

4. Large sample size and real-world program data improving external validity.

Major Concerns

1. Causal inference remains overstated despite revisions. The design is repeated cross-sectional with no control group.

2. Residual confounding remains due to unmeasured and time-varying factors (seasonality, macroeconomic changes, NGO activities).

3. Exposure definition using survey year remains conceptually weak.

4. Lack of full standardized mean difference (SMD) balance diagnostics reduces transparency.

5. Effect size interpretation may be optimistic; absolute differences should be emphasized more.

Minor Concerns

1. Some sections still contain overly generalized interpretations.

2. Figures and tables improved but could be more self-explanatory.

3. Intervention fidelity details remain limited.

Overall Interpretation

The authors have significantly improved the manuscript. However, the study design limits causal inference, and findings should be interpreted as associations rather than effects.

The manuscript is suitable for publication after minor revision, provided interpretation is further moderated.

Recommendation

Minor Revision

Key requirements:

- Remove residual causal language

Response: Thank you for this valuable comment. We have carefully revised the entire manuscript to remove residual causal language and to present the findings more appropriately as associations rather than causal effects. The wording has been revised in several sections, including page 17, lines 337-340; page 19, line 368; the title of Table 3; page 22, line 414; and page 23, line 425. These revisions were made to ensure that the interpretation of the results is consistent with the study design.

- Strengthen discussion of limitations

Response: Thank you for your helpful suggestion. We have strengthened the limitations section in the discussion to more clearly acknowledge the methodological limitations of the study, including issues related to residual confounding and interpretation of the findings. Please see page 27, lines 503-505.

- Add balance diagnostics clarity if possible

Response: Thank you for this comment. We have now added a new figure 4 (love plot) showing the standardized mean differences (SMDs) for comparing covariate balance between two treatment groups. The figure demonstrates how weighting improved the balance of observed covariates between the comparison groups. We have also added a description of this figure in the results section. Please see pages 20-21, lines 385-391.

- Emphasize absolute effects alongside risk ratios

Response: Thank you for this useful suggestion. We have revised the analysis to include absolute effect estimates alongside the risk ratios. Specifically, we added two additional binomial models to present the estimated absolute differences in food security and dietary diversity. These results are now presented in the revised Table 4 and described in the results section on page 22, lines 413-416 and 425-427.

Reviewers 2

Reviewer #2: I thank the authors for the detailed response to the previous comments. Many points have been addressed satisfactorily. However, several of the original concerns remain unresolved, and I have identified a few additional issues that require attention before the manuscript is suitable for publication.

1. The authors stated they had softened causal language throughout the manuscript. However, in the submitted revision this language does not appear consistently, e.g + The Results section (lines 404 and 411) attributes findings to the intervention with the phrases "due to the intervention." Such passages must be revised to reflect the observational, associational nature of the study.

Response: Thank you for your comment. We have deleted the lines that used this type of causal language. Please see (page 19, lines 368-370), (page 22, lines 414), and (page 23, lines 420-424).

2. Similarly, line 338 states: "The prevalence of household food security among vulnerable women got relatively better, from 9% in 2022 to 34% in 2024." The phrase "got relatively better" is casual and does not meet academic writing standards. Please revise.

Response: Thank you for this observation. We agree that the phrase "got relatively better" was informal and not suitable for academic writing. Accordingly, we revised the sentence to use more precise and formal language. The revised text now reads:

” The prevalence of household food security among vulnerable women showed a substantial increase, rising from 9% in 2022 to 34% (p<0.001) in 2024.”

This change has been incorporated into the manuscript (page 17, line 336-344).

3. The response stated that the paragraph mentioning women's empowerment had been removed from the Discussion. However, lines 491 to 499 about the intervention remains: "…contributed to enhanced women's empowerment" and "likely facilitated their greater involvement in household decision-making processes". As noted previously, this goes beyond the empirical findings.

Response: Thank you for your comment. The statements referring to women's empowerment and involvement in household decision-making have been removed from the revised manuscript in the first revision, as they were not directly supported by the empirical findings. These changes are reflected in the clean version of the manuscript in both the first and current versions. Please see the removed part in the relevant discussion section of the revised manuscript (track changes version of the previous (R1) version), where we deleted it. Please see page-26, 493-499.

4. The abstract (line 41–42) reports RR=3.29 (95% CI: 2.88–3.77) for dietary diversity. However, RR=3.29 is the unweighted modified Poisson regression estimate. The primary result, and the one correctly reported for food security in the same abstract is the IPSW (weighted) estimate. For dietary diversity, the IPSW result is RR=2.78. Reporting the unweighted estimate in the abstract as the headline finding is inconsistent with the study's stated analytic approach.

Response: Extremely sorry for the inconvenience, and thanks a lot for pointing that out. We agree with your comment and have revised the relevant portion of the abstract. Please see page 3, lines 39-42.

5. References 16 and 30 cite URLs beginning with "chrome-extension://...". Readers and editors cannot follow these links. The authors must replace these with the actual publicly available URLs, DOIs, or complete archival source information.

Response: Thank you for your comment, and extremely sorry for overlooking it. We have revised the links. Please see page-31, line- 603-604 and page-32, line- 647-649.

Reviewers 3

Reviewer #3: Thank you for the opportunity to review the revised manuscript. The authors have addressed most of my comments satisfactorily. However, I believe that Comment 9 regarding the accuracy of data presentation has not been fully addressed.

In this particular comment, my main concern was not related to the numbering or labeling of Figures 1 and 2, but rather to the inconsistency between the narrative text and the data presented in the figures. The manuscript reports a statistically significant improvement in household food security (p<0.001) and refers readers to a figure 2 as supporting evidence. However, the figure itself does not present p-values or statistical test results. As a result, it remains unclear where the reported p-value originates and whether it is derived from a table or a separate statistical analysis.

While the authors indicate that the figure labels were mistakenly interchanged and have now been corrected, this does not resolve the underlying issue. I recommend that the authors explicitly identify the source of the statistical results and ensure that all references to figures and tables accurately correspond to the data being presented throughout the Results section.

Therefore, I consider this comment to be only partially addressed and would appreciate further clarification from the authors before a final decision is made.

Response: Thank you for this important clarification. We agree that the concern was related not only to figure numbering but also to the consistency between the narrative text and the statistical information presented in relation to the figures. We have now revised the Results section to clearly indicate that the figure presents unweighted percentage/proportion changes in food security and dietary diversity, whereas the reported p-values were obtained from the corresponding statistical comparison. To avoid ambiguity, we have added a note below the figure stating that percentages are unweighted sample proportions and that the between-group differences for food security status and dietary diversity status were statistically significant (p < 0.001). We have also checked the figure and table references throughout the results section to ensure that each statement corresponds accurately to the data being presented. Please see page 17, line 343-345.

Attachment Submitted filename: Response_to_Reviewers_auresp_2.docx

# Peer review: Decision Letter 2

**DOI:** 10.1371/journal.pone.0362124.r005

**Authors:** Md. Feroz Kabir

26 Aug 2026

Please submit your revised manuscript by Oct 10 2026 11:59PM. If you will need more time than this to complete your revisions, please reply to this message or contact the journal office at plosone@plos.org. When you're ready to submit your revision, log on to https://www.editorialmanager.com/pone/ and select the 'Submissions Needing Revision' folder to locate your manuscript file.

- A letter that responds to each point raised by the academic editor and reviewer(s). You should upload this letter as a separate file labeled 'Response to Reviewers'.
- A marked-up copy of your manuscript that highlights changes made to the original version. You should upload this as a separate file labeled 'Revised Manuscript with Track Changes'.
- An unmarked version of your revised paper without tracked changes. You should upload this as a separate file labeled 'Manuscript'.

If you would like to make changes to your financial disclosure, please include your updated statement in your cover letter. Guidelines for resubmitting your figure files are available below the reviewer comments at the end of this letter.

If applicable, we recommend that you deposit your laboratory protocols in protocols.io to enhance the reproducibility of your results. Protocols.io assigns your protocol its own identifier (DOI) so that it can be cited independently in the future. For instructions see: https://journals.plos.org/plosone/s/submission-guidelines#loc-laboratory-protocols. Additionally, PLOS One offers an option for publishing peer-reviewed Lab Protocol articles, which describe protocols hosted on protocols.io. Read more information on sharing protocols at https://plos.org/protocols?utm_medium=editorial-email&utm_source=authorletters&utm_campaign=protocols.

As the corresponding author, your ORCID iD is verified in the submission system and will appear in the published article. PLOS supports the use of ORCID, and we encourage all coauthors to register for an ORCID iD and use it as well. Please encourage your coauthors to verify their ORCID iD within the submission system before final acceptance, as unverified ORCID iDs will not appear in the published article. *Only*  the individual author can complete the verification step; PLOS staff *cannot*  verify ORCID iDs on behalf of authors.

We look forward to receiving your revised manuscript.

Kind regards,

Md. Feroz Kabir, PhD, BPT, MPT, MPH, BPED, MPED

Academic Editor

PLOS One

Journal Requirements:

1. If the reviewer comments include a recommendation to cite specific previously published works, please review and evaluate these publications to determine whether they are relevant and should be cited. There is no requirement to cite these works unless the editor has indicated otherwise.

2. Please review your reference list to ensure that it is complete and correct. If you have cited papers that have been retracted, please include the rationale for doing so in the manuscript text, or remove these references and replace them with relevant current references. Any changes to the reference list should be mentioned in the rebuttal letter that accompanies your revised manuscript. If you need to cite a retracted article, indicate the article’s retracted status in the References list and also include a citation and full reference for the retraction notice.

Additional Editor Comments:

Properly format the manuscript and do appropriate grammar correction and submit the revised manuscript.

[Note: HTML markup is below. Please do not edit.]

Reviewer's Responses to Questions

**Comments to the Author**

Reviewer #1: (No Response)

Reviewer #2: All comments have been addressed

Reviewer #3: (No Response)

**********

2. Is the manuscript technically sound, and do the data support the conclusions?

Reviewer #1: (No Response)

Reviewer #2: Yes

Reviewer #3: Yes

**********

3. Has the statistical analysis been performed appropriately and rigorously?

Reviewer #1: (No Response)

Reviewer #2: Yes

Reviewer #3: Yes

**********

4. Have the authors made all data underlying the findings in their manuscript fully available?

The PLOS Data policy

Reviewer #1: (No Response)

Reviewer #2: No

Reviewer #3: Yes

**********

5. Is the manuscript presented in an intelligible fashion and written in standard English?

Reviewer #1: (No Response)

Reviewer #2: Yes

Reviewer #3: Yes

**********

Reviewer #1: Overall Assessment of Reviewer #1

My concerns have been substantially addressed, particularly regarding the study limitations, residual confounding, covariate balance, and presentation of absolute effects. However, I do not consider the response fully satisfactory yet. The main remaining problem is that the authors state that causal language has been removed, while several causal or intervention-effect expressions remain in the revised manuscript. Because the study uses repeated cross-sectional data without randomization or a concurrent control group, these expressions should be corrected before acceptance.

Comment 1: Causal Interpretation and Residual Confounding

Assessment: Partially addressed. The authors have made a strong effort to acknowledge the limitations of the study design. The revised Discussion now recognizes residual confounding from unmeasured time-varying factors and explicitly notes that seasonal variation, food prices, dietary patterns, climatic shocks, local economic conditions, government support, and other development activities may have influenced the observed differences. The manuscript also states that the observed differences cannot be attributed solely to the intervention. This part of the response is appropriate.

However, the response is not completely consistent with the revised manuscript. Reviewer #1 specifically requested that the findings be interpreted as associations rather than causal effects. Despite the authors’ statement that they have removed causal language, causal expressions remain in the Abstract, Results, and Conclusion.

Example still present in the manuscript: “The substantial improvements were likely driven by structured IGA implementation, with asset transfers, direct cash support, and nutrition education.”

Recommended replacement: “The observed improvements coincided with implementation of the structured IGA program, although the contribution of other temporal and contextual factors cannot be excluded.”

Another example is the Results wording: “The results showed a significant association over the time period due to the intervention.” This still attributes the observed association to the intervention.

Recommended replacement: “The results showed a significant difference in outcome prevalence between the 2022 and 2024 survey rounds after propensity score weighting.”

Comment 2: Study Design and Limitations

Assessment: Adequately addressed. The revised limitations section is substantially stronger than the previous version. It acknowledges that the analysis was based on two cross-sectional survey rounds and that propensity score weighting cannot eliminate bias from unmeasured factors. It also discusses differences in survey timing, short recall periods, seasonal variation, food prices, dietary patterns, climatic shocks, economic conditions, government support, and other development actors.

The manuscript appropriately states that future research should establish causality and acknowledges that the study did not fully adhere to a rigorous impact evaluation design. This directly responds to Reviewer #1’s concern.

Recommended wording to retain or strengthen: “Because the study was based on two repeated cross-sectional survey rounds without random assignment or a concurrent control group, residual confounding and other time-varying contextual factors cannot be excluded. Therefore, the observed changes should be interpreted as associations rather than definitive causal effects of the intervention.”

Comment 3: Propensity Score Weighting and Covariate Balance

Assessment: Mostly addressed, but additional reporting would improve the response. The authors added a standardized mean difference/Love plot showing covariate balance before and after inverse probability weighting. This is an appropriate response to Reviewer #1’s request.

However, the manuscript currently states that “most covariates” moved closer to zero and remained within acceptable limits. If the intended criterion is an absolute standardized mean difference below 0.10, the manuscript should explicitly state this and report whether all covariates met that threshold after weighting.

Recommended wording, if supported by the actual data: “After inverse probability weighting, all absolute standardized mean differences were below 0.10, indicating adequate balance across the measured covariates.”

If one or more covariates remain above 0.10, the authors should report this transparently rather than stating that all covariates were adequately balanced.

Comment 4: Propensity Score Overlap and Positivity

Assessment: Partially addressed or insufficiently documented. The manuscript presents propensity-score distributions and describes them as showing good overlap. However, the reported distributions differ substantially across some propensity-score categories. This does not necessarily invalidate the analysis, but the authors should provide a clearer assessment of common support and weighting diagnostics.

The authors should consider reporting whether extreme propensity scores or extreme weights were present, whether the weights were stabilized or truncated, and whether the effective sample size changed substantially after weighting. These details would make the inverse probability weighting analysis more transparent and reproducible.

Recommended wording: “The propensity-score distributions showed overlap across the observed range, although their distributions differed between survey rounds. Weight diagnostics were examined to assess the influence of extreme propensity scores and weights.”

Comment 5: Absolute Effects Alongside Relative Effects

Assessment: Adequately addressed statistically, but terminology should be corrected. The authors added absolute effect estimates using the weighted binomial model. For household food security, the weighted estimate corresponds to a 0.16 increase in probability, or a 16-percentage-point difference. For dietary diversity, the corresponding estimate is 0.53, or a 53-percentage-point difference. This appropriately complements the relative risk estimates.

The remaining problem is terminology. The manuscript labels these estimates as “average intervention effects.” Given the observational repeated cross-sectional design, this terminology can imply a causal treatment effect.

Recommended replacement for the Results/Table heading: “Weighted differences in outcome prevalence between 2022 and 2024.”

Recommended wording: “The weighted binomial model estimated a 0.16 higher probability of household food security in 2024 compared with 2022 (95% CI: 0.09–0.22), corresponding to a 16-percentage-point difference.”

Comment 6: Overall Consistency Between the Response Letter and Manuscript

Assessment: This requires correction before acceptance. The response to Reviewer #1 states that the authors carefully revised the manuscript to remove residual causal language. However, the revised manuscript still contains terms such as “impact,” “intervention effects,” “average intervention effects,” “due to the intervention,” “likely driven by,” and “effective in improving.”

This creates a discrepancy between the response letter and the actual manuscript. The reviewer may reasonably identify this discrepancy in another review round. The authors should therefore perform a final search of the entire manuscript and response letter for causal terminology and revise every occurrence consistently.

Recommended Manuscript-Wide Replacements

• “impact of the intervention” → “changes associated with program implementation”

• “intervention effects” → “weighted associations” or “differences between survey rounds”

• “average intervention effect” → “weighted difference in outcome prevalence”

• “due to the intervention” → “coinciding with program implementation”

• “likely driven by the intervention” → “may have coincided with program implementation”

• “interventions are effective in improving” → “intervention implementation was positively associated with”

• “caused by” → “associated with” or “observed alongside”

Suggested Revised Conclusion

“In this repeated cross-sectional study, household food security and dietary diversity were substantially higher in 2024 than in 2022 among vulnerable women in the study areas. These differences remained after propensity score weighting, indicating a positive association between program implementation and the study outcomes. However, because the study was non-randomized and based on two cross-sectional survey rounds, residual confounding and other time-varying contextual factors cannot be excluded. Therefore, the observed differences should not be interpreted as definitive causal effects of the intervention. Future controlled or longitudinal evaluations are needed to establish the causal effectiveness and long-term sustainability of integrated nutrition-sensitive interventions.”

Reviewer #2: (No Response)

Reviewer #3: Thank you for the opportunity to review the revised manuscript and for the authors’ efforts to address my previous comments. However, I believe that the concern regarding the statistical interpretation of Figure 2 remains unresolved.

Figure 2 is a descriptive chart presenting changes in household food security status and dietary diversity between 2022 and 2024. The figure itself does not present the results of a statistical analysis, yet the Results section (Page 17, lines 335–340) reports statistically significant changes with corresponding p-values. The authors should therefore distinguish between the descriptive percentages presented in Figure 2 and the inferential analysis used to obtain the reported p-values. Simply stating in the figure note that these differences are statistically significant does not address the underlying issue of how and from which statistical analysis the reported p-values were obtained.

I recommend that the authors explicitly state which statistical method or model generated these p-values and clarify whether they were derived from the propensity-score-weighted analysis or from a separate statistical test. The figure can remain as a presentation of unweighted sample proportions, but its note should clearly indicate that the reported p-values are derived from the specified statistical analysis. This would somehow resolve the ambiguity regarding the source and interpretation of the reported statistical significance.

The authors may also consider presenting these results in a table rather than a figure, as a table would allow the 2022 and 2024 proportions, magnitude of change, and corresponding statistical results to be presented together. However, regardless of whether a figure or table is retained, the statistical method used to generate the reported p-values should be explicitly stated.

**********

what does this mean?). If published, this will include your full peer review and any attached files.

If you choose “no”, your identity will remain anonymous but your review may still be made public.

**Do you want your identity to be public for this peer review?**  For information about this choice, including consent withdrawal, please see our Privacy Policy

Reviewer #1: No

Reviewer #2: No

Reviewer #3: No

**********

[NOTE: If reviewer comments were submitted as an attachment file, they will be attached to this email and accessible via the submission site. Please log into your account, locate the manuscript record, and check for the action link "View Attachments". If this link does not appear, there are no attachment files.]

To ensure your figures meet our technical requirements, please review our figure guidelines: https://journals.plos.org/plosone/s/figures

You may also use PLOS’s free figure tool, NAAS, to help you prepare publication quality figures: https://journals.plos.org/plosone/s/figures#loc-tools-for-figure-preparation.

NAAS will assess whether your figures meet our technical requirements by comparing each figure against our figure specifications.

# Author comment: Author response to Decision Letter 3

**DOI:** 10.1371/journal.pone.0362124.r006

11 Oct 2026

Manuscript ID: PONE-D-26-04366R2

Manuscript title: Integrated nutrition-sensitive interventions are associated with improvements in household food security and dietary diversity among vulnerable women in the host communities of Cox’s Bazar, Bangladesh: Findings from a pre-post comparison study

Reviewers 1

Reviewer #1: Overall Assessment of Reviewer #1

My concerns have been substantially addressed, particularly regarding the study limitations, residual confounding, covariate balance, and presentation of absolute effects. However, I do not consider the response fully satisfactory yet. The main remaining problem is that the authors state that causal language has been removed, while several causal or intervention-effect expressions remain in the revised manuscript. Because the study uses repeated cross-sectional data without randomization or a concurrent control group, these expressions should be corrected before acceptance.

Response: Thank you very much for your careful review and constructive comment. We agree that some casual or informal language has been used in the manuscript. Therefore, we carefully reviewed the entire manuscript and revised the language to ensure a more appropriate academic and scientific tone. Details of these revisions are provided in our responses to the specific comments below.

Comment 1: Causal Interpretation and Residual Confounding

Assessment: Partially addressed. The authors have made a strong effort to acknowledge the limitations of the study design. The revised Discussion now recognizes residual confounding from unmeasured time-varying factors and explicitly notes that seasonal variation, food prices, dietary patterns, climatic shocks, local economic conditions, government support, and other development activities may have influenced the observed differences. The manuscript also states that the observed differences cannot be attributed solely to the intervention. This part of the response is appropriate.

However, the response is not completely consistent with the revised manuscript. Reviewer #1 specifically requested that the findings be interpreted as associations rather than causal effects. Despite the authors’ statement that they have removed causal language, causal expressions remain in the Abstract, Results, and Conclusion.

Example still present in the manuscript: “The substantial improvements were likely driven by structured IGA implementation, with asset transfers, direct cash support, and nutrition education.”

Recommended replacement: “The observed improvements coincided with implementation of the structured IGA program, although the contribution of other temporal and contextual factors cannot be excluded.”

Another example is the Results wording: “The results showed a significant association over the time period due to the intervention.” This still attributes the observed association to the intervention.

Recommended replacement: “The results showed a significant difference in outcome prevalence between the 2022 and 2024 survey rounds after propensity score weighting.”

Response: Thank you for your careful assessment and for highlighting the remaining inconsistencies between our responses and the revised manuscript. We agree with the highlighted points. In response to your comment, we carefully reviewed the Abstract, Results, Discussion, and Conclusion sections again and removed and sometimes revised the remaining causal language. Specifically, we replaced causal terms (e.g., “effect,” “impact,” “led to,” “resulted in,” “improved,” and similar wording where applicable) with more appropriate observational terms such as “association,” “relationship,” “difference,” “correlation,” and “was observed to be associated with.” We have also revised the interpretation in the Discussion and Conclusion sections to ensure that the findings are presented as associations, rather than causal estimates.

Your suggestion was to change the first line of the conclusion; however, we revised the entire conclusion to better align with our findings. Please see page 3, lines 48-53.

Similarly, the Results wording: “The results showed a significant association over the time period due to the intervention” has been revised to: “The results showed a significant difference in outcome prevalence between the 2022 and 2024 survey rounds after propensity score weighting.” Please see page-21, line-396-397.

Also please see page-2, line-27; page-2, line-35; page-3, line-48-53; page-6-7, line-125-126; page-7, line-127; page-13, line-279; page-15, line-288,296; page-20, line-381; page-21, line 401-405, page-22, line-418-419; page-27, line-505.

Comment 2: Study Design and Limitations

Assessment: Adequately addressed. The revised limitations section is substantially stronger than the previous version. It acknowledges that the analysis was based on two cross-sectional survey rounds and that propensity score weighting cannot eliminate bias from unmeasured factors. It also discusses differences in survey timing, short recall periods, seasonal variation, food prices, dietary patterns, climatic shocks, economic conditions, government support, and other development factors.

The manuscript appropriately states that future research should establish causality and acknowledges that the study did not fully adhere to a rigorous impact evaluation design. This directly responds to Reviewer #1’s concern.

Recommended wording to retain or strengthen: “Because the study was based on two repeated cross-sectional survey rounds without random assignment or a concurrent control group, residual confounding and other time-varying contextual factors cannot be excluded. Therefore, the observed changes should be interpreted as associations rather than definitive causal effects of the intervention.”

Response: Thank you so much for your feedback. As per your suggestion, we have added the recommended wording to strengthen the discussion section. Please see page 26, lines 487-490.

Comment 3: Propensity Score Weighting and Covariate Balance

Assessment: Mostly addressed, but additional reporting would improve the response. The authors added a standardized mean difference/Love plot showing covariate balance before and after inverse probability weighting. This is an appropriate response to Reviewer #1’s request.

However, the manuscript currently states that “most covariates” moved closer to zero and remained within acceptable limits. If the intended criterion is an absolute standardized mean difference below 0.10, the manuscript should explicitly state this and report whether all covariates met this threshold after weighting.

Recommended wording, if supported by the actual data: “After inverse probability weighting, all absolute standardized mean differences were below 0.10, indicating adequate balance across the measured covariates.”

If one or more covariates remain above 0.10, the authors should report this transparently rather than stating that all covariates were adequately balanced.

Response: Thank you for this suggestion. We have updated the text for Fig 4, which shows that SMDs are within the limit of 0.10 for all covariates. Please see page-20, lines 376-379.

Comment 4: Propensity Score Overlap and Positivity

Assessment: Partially addressed or insufficiently documented. The manuscript presents propensity-score distributions and describes them as showing good overlap. However, the reported distributions differ substantially across some propensity-score categories. This does not necessarily invalidate the analysis, but the authors should provide a clearer assessment of common support and weighting diagnostics.

The authors should consider reporting whether extreme propensity scores or extreme weights were present, whether the weights were stabilized or truncated, and whether the effective sample size changed substantially after weighting. These details would make the inverse probability weighting analysis more transparent and reproducible.

Recommended wording: “The propensity-score distributions showed overlap across the observed range, although their distributions differed between survey rounds. Weight diagnostics were examined to assess the influence of extreme propensity scores and weights.”

Response: Thank you. We have updated the text accordingly and mentioned the positivity assumption; please see page-20, lines 383-387.

Comment 5: Absolute Effects Alongside Relative Effects

Assessment: Adequately addressed statistically, but terminology should be corrected. The authors added absolute effect estimates using the weighted binomial model. For household food security, the weighted estimate corresponds to a 0.16 increase in probability, or a 16-percentage-point difference. For dietary diversity, the corresponding estimate is 0.53, or a 53-percentage-point difference. This appropriately complements the relative risk estimates.

The remaining problem is terminology. The manuscript labels these estimates as “average intervention effects.” Given the observational repeated cross-sectional design, this terminology can imply a causal treatment effect.

Recommended replacement for the Results/Table heading: “Weighted differences in outcome prevalence between 2022 and 2024.”

Recommended wording: “The weighted binomial model estimated a 0.16 higher probability of household food security in 2024 compared with 2022 (95% CI: 0.09–0.22), corresponding to a 16-percentage-point difference.”

Response: We have updated the title of the table and text accordingly; please see page 19, line 373.

Comment 6: Overall Consistency Between the Response Letter and Manuscript

Assessment: This requires correction before acceptance. The response to Reviewer #1 states that the authors carefully revised the manuscript to remove residual causal language. However, the revised manuscript still contains terms such as “impact,” “intervention effects,” “average intervention effects,” “due to the intervention,” “likely driven by,” and “effective in improving.”

This creates a discrepancy between the response letter and the actual manuscript. The reviewer may reasonably identify this discrepancy in another review round. The authors should therefore perform a final search of the entire manuscript and response letter for causal terminology and revise every occurrence consistently.

Recommended Manuscript-Wide Replacements

• “impact of the intervention” → “changes associated with program implementation”

• “intervention effects” → “weighted associations” or “differences between survey rounds”

• “average intervention effect” → “weighted difference in outcome prevalence”

• “due to the intervention” → “coinciding with program implementation”

• “likely driven by the intervention” → “may have coincided with program implementation”

• “interventions are effective in improving” → “intervention implementation was positively associated with”

• “caused by” → “associated with” or “observed alongside”

Suggested Revised Conclusion

“In this repeated cross-sectional study, household food security and dietary diversity were substantially higher in 2024 than in 2022 among vulnerable women in the study areas. These differences remained after propensity score weighting, indicating a positive association between program implementation and the study outcomes. However, because the study was non-randomized and based on two cross-sectional survey rounds, residual confounding and other time-varying contextual factors cannot be excluded. Therefore, the observed differences should not be interpreted as definitive causal effects of the intervention. Future controlled or longitudinal evaluations are needed to establish the causal effectiveness and long-term sustainability of integrated nutrition-sensitive interventions.”

Response: Thank you for your valuable suggestion. We have revised the entire manuscript and deleted or revised the terms. Please see the revised terms in page-2, line-27; page-2, line-35; page-3, line-48-53; page-6-7, line-125-126; page-7, line-127; page-13, line-279; page-15, line-288,296; page-20, line-381; page-21, line 401-405, page-22, line-418-419; page-27, line-505. We have also revised the conclusion as per your suggestion; please see page-3, lines- 48-53, page-27-28, lines-522-530.

Reviewers 2

Reviewer #2: (No Response)

Reviewers 3

Reviewer #3: Thank you for the opportunity to review the revised manuscript and for the authors’ efforts to address my previous comments. However, I believe that the concern regarding the statistical interpretation of Figure 2 remains unresolved.

Figure 2 is a descriptive chart presenting changes in household food security status and dietary diversity between 2022 and 2024. The figure itself does not present the results of a statistical analysis, yet the Results section (Page 17, lines 335–340) reports statistically significant changes with corresponding p-values. The authors should therefore distinguish between the descriptive percentages presented in Figure 2 and the inferential analysis used to obtain the reported p-values. Simply stating in the figure note that these differences are statistically significant does not address the underlying issue of how and from which statistical analysis the reported p-values were obtained. [1.1]

I recommend that the authors explicitly state which statistical method or model generated these p-values and clarify whether they were derived from the propensity-score-weighted analysis or from a separate statistical test. The figure can remain as a presentation of unweighted sample proportions, but its note should clearly indicate that the reported p-values are derived from the specified statistical analysis. This would somehow resolve the ambiguity regarding the source and interpretation of the reported statistical significance.

The authors may also consider presenting these results in a table rather than a figure, as a table would allow the 2022 and 2024 proportions, magnitude of change, and corresponding statistical results to be presented together. However, regardless of whether a figure or table is retained, the statistical method used to generate the reported p-values should be explicitly stated.

Response: We have excluded the text that includes unadjusted p-values. We have shown the adjusted p-values in the later tables. We believe that these are no longer required. Please see page-17, lines-337-340.

Attachment Submitted filename: Response_to_Reviewers_auresp_3.docx

# Editor report: Decision Letter 3

**DOI:** 10.1371/journal.pone.0362124.r007

**Authors:** Md. Feroz Kabir

20 Oct 2026

Integrated nutrition-sensitive interventions are associated with improvements in household food security and dietary diversity among vulnerable women in the host communities of Cox’s Bazar, Bangladesh: Findings from a pre-post comparison study

PONE-D-26-04366R3

Dear Malay Kanti Mridha,

We’re pleased to inform you that your manuscript has been judged scientifically suitable for publication and will be formally accepted for publication once it meets all outstanding technical requirements.

Within one week, you’ll receive an e-mail detailing the required amendments. When these have been addressed, you’ll receive a formal acceptance letter and your manuscript will be scheduled for publication.

An invoice will be generated when your article is formally accepted. Please note, if your institution has a publishing partnership with PLOS and your article meets the relevant criteria, all or part of your publication costs will be covered. Please make sure your user information is up-to-date by logging into Editorial Manager at Editorial Manager® and clicking the ‘Update My Information' link at the top of the page. For questions related to billing, please contact billing support.

If your institution or institutions have a press office, please notify them about your upcoming paper to help maximize its impact. If they’ll be preparing press materials, please inform our press team as soon as possible -- no later than 48 hours after receiving the formal acceptance. Your manuscript will remain under strict press embargo until 2 pm Eastern Time on the date of publication. For more information, please contact onepress@plos.org.

Kind regards,

Md. Feroz Kabir, PhD, BPT, MPT, MPH, BPED, MPED

Academic Editor

PLOS One

Additional Editor Comments (optional):

Reviewers' comments:

# Editor report: Acceptance letter

**DOI:** 10.1371/journal.pone.0362124.r008

**Authors:** Md. Feroz Kabir

PONE-D-26-04366R3

PLOS One

Dear Dr. Mridha,

I'm pleased to inform you that your manuscript has been deemed suitable for publication in PLOS One. Congratulations! Your manuscript is now being handed over to our production team.

At this stage, our production department will prepare your paper for publication. This includes ensuring the following:

* All references, tables, and figures are properly cited

* All relevant supporting information is included in the manuscript submission,

* There are no issues that prevent the paper from being properly typeset

You will receive further instructions from the production team, including instructions on how to review your proof when it is ready. Please keep in mind that we are working through a large volume of accepted articles, so please give us a few days to review your paper and let you know the next and final steps.

Lastly, if your institution or institutions have a press office, please let them know about your upcoming paper now to help maximize its impact. If they'll be preparing press materials, please inform our press team within the next 48 hours. Your manuscript will remain under strict press embargo until 2 pm Eastern Time on the date of publication. For more information, please contact onepress@plos.org.

You will receive an invoice from PLOS for your publication fee after your manuscript has reached the completed accept phase. If you receive an email requesting payment before acceptance or for any other service, this may be a phishing scheme. Learn how to identify phishing emails and protect your accounts at https://explore.plos.org/phishing.

If we can help with anything else, please email us at customercare@plos.org.

Thank you for submitting your work to PLOS One and supporting open access.

Kind regards,

PLOS One Editorial Office Staff

on behalf of

Dr. Md. Feroz Kabir

Academic Editor

PLOS One
